# Users’ thoughts and opinions about a self-regulation-based eHealth intervention targeting physical activity and the intake of fruit and vegetables: A qualitative study

**DOI:** 10.1371/journal.pone.0190020

**Published:** 2017-12-21

**Authors:** Louise Poppe, Celien Van der Mispel, Ilse De Bourdeaudhuij, Maïté Verloigne, Samyah Shadid, Geert Crombez

**Affiliations:** 1 Department of Movement and Sports Sciences, Faculty of Medicine and Health Sciences, Ghent University, Ghent, Belgium; 2 Department of Experimental-Clinical and Health Psychology, Faculty of Psychology and Educational Sciences, Ghent University, Ghent, Belgium; 3 Department of Endocrinology, Ghent University Hospital, Ghent, Belgium; 4 Centre for Pain Research, University of Bath, Bath, United Kingdom; TNO, NETHERLANDS

## Abstract

**Purpose:**

EHealth interventions are effective in changing health behaviours, such as increasing physical activity and altering dietary habits, but suffer from high attrition rates. In order to create interventions that are adapted to end-users, in-depth investigations about their opinions and preferences are required. As opinions and preferences may vary for different target groups, we explored these in two groups: the general population and a clinical sample.

**Methods:**

Twenty adults from the general population (mean age = 42.65, 11 women) and twenty adults with type 2 diabetes (mean age = 64.30, 12 women) performed ‘MyPlan 1.0’, which is a self-regulation-based eHealth intervention designed to increase physical activity and the intake of fruit and vegetables in the general population. The opinions and preferences of end-users were explored using a think aloud procedure and a questionnaire. During a home visit, participants were invited to think aloud while performing ‘MyPlan 1.0’. The thoughts were transcribed verbatim and inductive thematic analysis was applied.

**Results:**

Both groups had similar opinions regarding health behaviours and ‘MyPlan 1.0’. Participants generally liked the website, but often experienced it as time-consuming. Furthermore, they regularly mentioned that a mobile application would be useful to remind them about their goals on a daily basis. Finally, users’ ideas about how to pursue health behaviours often hindered them to correctly use the website.

**Conclusions:**

Although originally created for the general population, ‘MyPlan 1.0’ can also be used in adults with type 2 diabetes. Nevertheless, more adaptations are needed to make the eHealth intervention more convenient and less time-consuming. Furthermore, users’ ideas regarding a healthy lifestyle should be taken into account when designing online interventions.

## Introduction

The prevalence of many chronic diseases, such as type 2 diabetes, cardiovascular diseases and cancer, is high and still rising [1]. A healthy lifestyle, including sufficient physical activity (PA) and fruit and vegetable intake, can prevent these diseases, or alter their negative consequences [2–6]. However, only 35% of the Western adult population follows the guidelines of 30 minutes moderate- to vigorous PA on 5 days per week (and preferably every day), whereas 24% meets the norm of eating 5 or more servings of fruit and vegetables per day [6–8]. Even people for whom having a healthy lifestyle is key in the management of their disease, such as patients with type 2 diabetes, often fail to be physically active on a regular basis and fail to conform to a disease-specific dietary regime [9–11]. Consequently, there is a strong need for effective and easy-to-implement interventions that target a healthy lifestyle.

Internet-based interventions have the potential to reach a large part of the population, while still being able to offer a personal approach. Indeed, via computer tailoring e- (electronic) health features can be adapted to the user’s specific needs. Such interventions are promising in changing health behaviours [12], such as increasing PA levels and altering dietary habits [13–18], especially when interventions are informed by solid theory and use behaviour change techniques that are evidence-based [16, 19–21]. However, even when eHealth interventions are theory- and evidence-based, attrition is often high and affects the potential effect of these programmes [22, 23]. In order to address this challenge, it has been recommended to involve users in the design and testing of eHealth tools [24]. The experience and opinion of end-users about core elements of online interventions may further guide eHealth development and understand eHealth usage [25]. As eHealth interventions are often used in various target groups [26], it is important to investigate possible differences in perceptions, opinions and preferences between user groups. Only by doing so the needs of different target populations can be taken into account.

In this paper, we investigate the perspectives of users on a web-based programme (the website ‘MyPlan 1.0’) that aims to increase PA and fruit and vegetable intake. ‘MyPlan 1.0’ is grounded in self-regulation theory [27, 28], and guides users through the processes of behaviour change via different evidence-based techniques, such as setting specific goals (goal-setting), deciding how these goals can be reached (action planning), foreseeing barriers and possible solutions (coping planning) and monitoring the behaviour change process. These behavioural targets and self-regulation techniques are relevant for the general population as well as for individuals with chronic diseases [29]. The potential use of ‘MyPlan 1.0’ by patients with type 2 diabetes was also suggested by general practitioners who reflected upon the usefulness of that intervention for their practice [30]. Indeed, disease management of patients with type 2 diabetes also includes alterations in PA and diet [31]. Developing an inclusive tool may then be non-stigmatizing and more cost-effective than creating an intervention specifically designed for adults with type 2 diabetes.

‘MyPlan 1.0’ is effective in increasing PA and the intake of fruit and vegetables (trial registration on ClinicalTrials.gov: NCT02211040) [32, 33]. Adults of the general population perceive the intervention as feasible and acceptable [32, 34]. Notwithstanding, only 24% of the users completed the entire intervention [34]. Such high attrition rates may indicate that ‘MyPlan 1.0’ was designed too much from a top-down perspective (i.e. from a theoretical point of view) and highlights the need for more in-depth research regarding the users’ perspectives. Assessing users’ experiences while performing the online programme may guide further adaptations to the programme, and may also inform other researchers about how possible end-users experience online programmes and how programmes can be adapted to better meet the users’ needs.

The aim of this paper is to understand the experience of potential users, both adults from the general population and individuals with type 2 diabetes, via a think aloud procedure and a self-report questionnaire. Because self-report questionnaires only allow general inferences and are sensitive to recall bias, we also used a think aloud procedure to assess users’ thoughts while performing each step of the programme. In so doing, users provide more immediate reactions in comparison with the opinions expressed in retrospective focus groups and interviews.

## Methods

### Participants

We wanted to include twenty participants from the general population and twenty participants with type 2 diabetes. The intended sample size was based on a similar study using a think aloud interview [35]. Furthermore, the meta-analysis of Hwang and Salvendy (2010) indicated that 10±2 participants is sufficient for usability tests such as the think aloud method [36].

The participants from the general population were recruited via an available database, consisting of individuals who had expressed their interest to participate in studies of the Ghent Health Psychology Research Group via a website (http://www.healthpsychology.ugent.be/vrijwilligers), and via the snowball sampling technique. Participants were purposively sampled to have an equal distribution of men versus women, participants with low versus high education level and younger versus older persons. Inclusion criteria were being ≥ 18 years old and Dutch speaking. Participants with type 2 diabetes were recruited via advertisements distributed by the Diabetes Association Flanders and the Ghent University Hospital and via the snowball sampling technique. For this group, we aimed to create an equal distribution in men versus women. Patients had to be ≥ 18 years old, Dutch speaking and being ≥ 1 month post-diagnosis to be eligible for participation in the study.

Six persons from the general population were not willing to take part in the study. One person with type 2 diabetes could not participate because she did not have a computer. Consequently, another person with type 2 diabetes was recruited. Some of the participants were acquaintances of the interviewers.

The study was approved by the Committee of Medical Ethics of the Ghent university hospital (B670201526613) and written informed consent was obtained. There was no reimbursement for participation in the study.

### The intervention ‘My Plan 1.0’

‘MyPlan 1.0’ is an eHealth tool designed to increase PA and the intake of fruit and vegetables in the general population [37]. The fully-automated and freely accessible website (www.mijnactieplan.be) incorporates several self-regulation techniques [27] and consists of three modules: a start module, a first follow-up module (one week after the start module) and a second follow-up module (one month after the start module).

**Fig 1:**
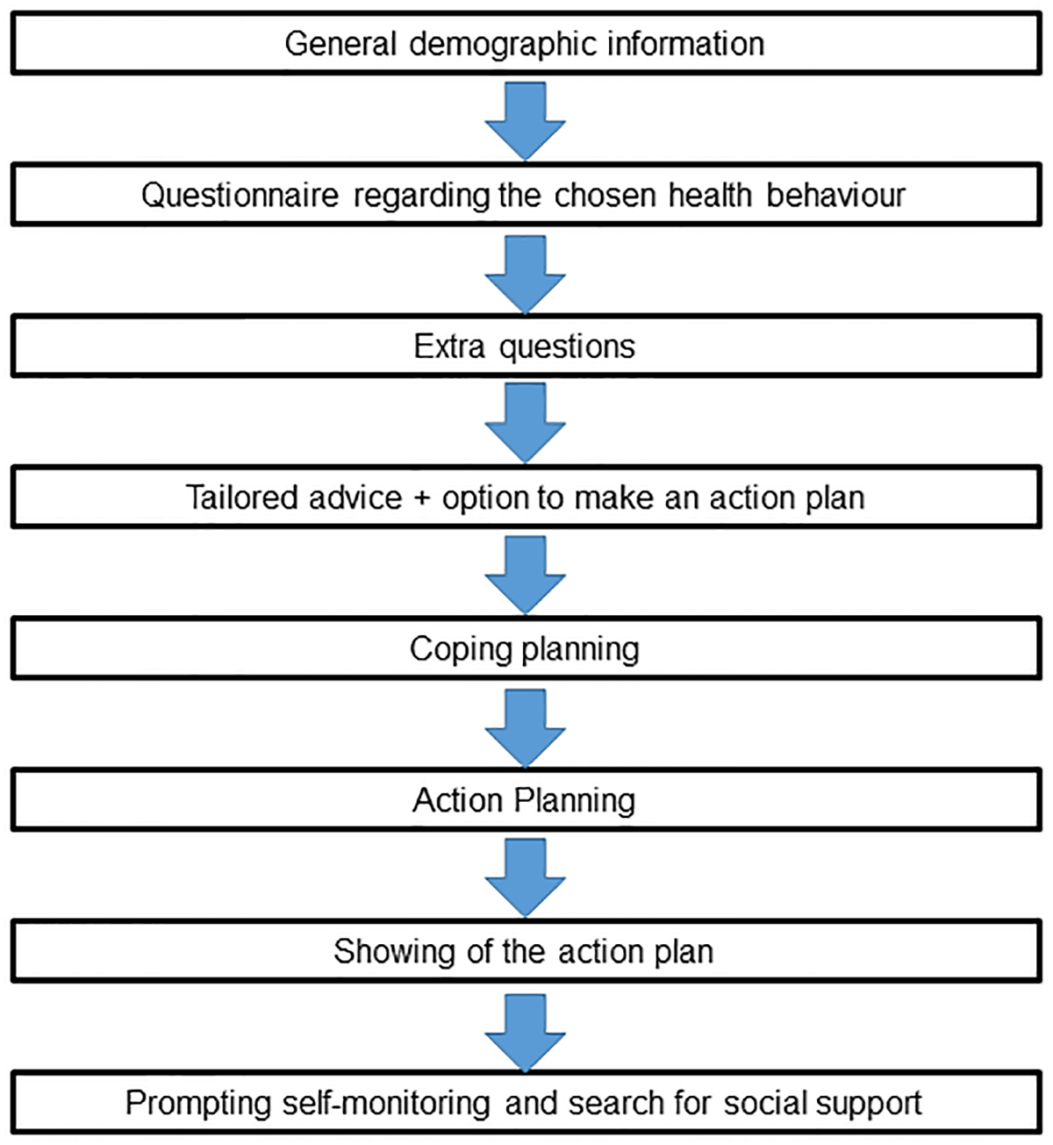
Overview of the start module.

Fig 1 displays an overview of the components of the start module and the order in which they appear. First, users choose whether they would like to increase their PA, their intake of fruit, or their intake of vegetables, and answer questions assessing demographic information. Thereafter, they fill out a questionnaire to assess the baseline level of their selected health behaviour. PA is assessed by the International Physical Activity Questionnaire (IPAQ-L) [38]. Fruit and vegetable intake is assessed by the Flemish Fruit Test and Vegetable Test [39]. Users also answer questions about personal determinants for behaviour change, such as outcome-expectancies and self-efficacy. This is done for research purposes. Next, users receive tailored feedback based on their answers on the questionnaire assessing the chosen health behaviour, and are asked whether they would like to make a plan to change this behaviour. Users can decide to make a plan or leave the website. By going through the coping and action planning components, users respectively look for solutions to tackle possible barriers (e.g. “I will put my running shoes at the door so I don’t forget about my plan”) and create their own specific plan to be more physically active, to eat more fruit or to eat more vegetables (e.g. “Every Tuesday I will run for 30 minutes”). During this process, users have the opportunity to create implementation intentions in the form of an if-then plan (e.g. “If I come home from work, I will run for 30 minutes in the neighbourhood) [40]. Their specific plans together with possible barriers and solutions are then shown in a printable format. Finally, the website invites users to monitor their behaviour change and to send their plan to friends and family in order to receive social support.

During the follow-up modules (one week and one month after the start module), users complete questions to assess whether they have been able to change the health behaviour of their choice and to receive tailored feedback on their change. Thereafter users have the possibility to adapt their plan based upon the success or failure of their previous plan.

### Protocol and procedures

The protocol of the think aloud procedure was based on a think aloud study by Yardley and colleagues (2010) [35]. Two female researchers (LP and CVDM) and two female master students in Clinical Psychology (ED and VVH) performed the think aloud procedure. LP has a Master’s degree in Experimental and Theoretical Psychology. CVDM has a Master’s degree in Clinical Psychology. The master students were trained by LP and CVDM. Participants were visited at home by one of the researchers. They first completed a questionnaire assessing demographic information. Participants with type 2 diabetes also reported the time since their diagnosis. Participants received the following instructions for the think aloud procedure:

“‘MyPlan’ helps people to live a healthier life. Via this tool you can choose whether you like to eat more vegetables, consume more fruit or increase your physical activity. ‘MyPlan’ will give you advice about your current health behaviours and help you with making a plan to change these behaviours step by step. Currently, we try to improve the programme, and you can help us with this. We will require you to perform the programme and develop your own plan. When going through the programme, please state out aloud what comes to your mind. Please do not refrain from giving critical remarks, as we can learn a lot from these comments. Also positive experiences can be stated.”

To let participants become acquainted with verbalizing their thoughts, a short exercise was provided. Participants were instructed to imagine their house and count the windows. They had to state out aloud how they imagined walking through each room and counting the windows. After the exercise, participants went through the website on their own computer at their own pace. Verbalizations were voice-recorded and the computer screen was filmed via a tablet. In contrast to the standard intervention, participants were instructed to start the first follow-up module immediately after completing the start module. Participants did not perform the second follow-up session, because it is similar to the first follow-up session.

When participants forgot to verbalize their thoughts, prompts such as “please try to say out aloud what comes to your mind” or “what comes to your mind when you see this?” were given by the researcher. When participants completed the second module of the programme, the researcher asked them how they generally perceived the website and what they liked or disliked.

Finally, participants filled out the Dutch version of the Website Evaluation Questionnaire [41]. This questionnaire consists of three subscales, each having three items. The first subscale assesses users’ perceptions regarding the personal relevance of the eHealth tool. The second subscale measures the extent to which users experience the tool enjoyable and attractive (i.e. their engagement with the tool). The third subscale assesses whether users feel like the tool helped them with self-assessment and goal-setting. All items are rated on a Likert-scale, ranging from 1 (strongly disagree) to 5 (strongly agree).

The home visits were carried out from March 2016 until May 2016, and lasted approximately 75 minutes.

### Data analysis

For the Website Evaluation Questionnaire, sum scores for each scale were calculated by adding the scores for each item of a subscale (minimum = 1; maximum = 5), resulting in a possible maximum score of 15 for each scale.

Inductive thematic analysis was conducted in order to identify recurring patterns in participants’ perceptions about ‘MyPlan 1.0’. Thematic analysis has been defined as “a method for identifying, analysing and reporting patterns (themes) within data” ([42] page 6). Inductive or “bottom-up” thematic analysis codes the data without a pre-existing framework to do so [42]. This technique was chosen for two reasons. First, by using a data-driven way of coding we maximally explore users’ perceptions instead of framing their ideas according to a specific theory. Second, we wanted to explore whether different themes would emerge as a function of group, i.e. the participants from the general population and the type 2 diabetes group. In order to code the data we followed the analysis process described by Braun and Clarke (2006).

In a first step, all the recordings were transcribed verbatim. None of the transcripts were returned to the participants for comments. Two researchers (CVDM and LP) read the transcripts to get acquainted with the data. While reading, some general findings were written down. In the second step, the data were read again, and initial codes were generated using qualitative data analysis software nVivo 11 (QSR International Pty. Ltd. Version 11, 2015). During this phase, no limit was set on the amount of generated codes. CVDM and LP each coded the data of 10 randomly selected participants from the general population and 10 participants with type 2 diabetes. In the third step, codes related to different opinions or experiences (e.g. codes expressing opinions regarding lay-out versus codes expressing experiences of awareness) identified in more than one participant’s transcript were brought together in different themes and a first differentiation between main and subthemes was established (e.g. opinions regarding lay-out and user-friendliness are both related to the design of the website, but not to the usefulness of the website). The codes were now classified under the themes according to the principles of internal homogeneity and external heterogeneity. Themes with a low number of codes were removed or integrated within other themes. This phase was carried out by CVDM for the data of the participants from the general population, whereas LP did the same for the data of the participants with type 2 diabetes. Next, all data was read again but with the identified themes in mind. This was done to check whether the data was well captured by the themes. Finally, CVDM and LP discussed and defined the final themes for both groups. If no consensus was reached, a third researcher (LD) was consulted. The participants did not provide feedback on the findings. The completed version of the consolidated criteria for reporting qualitative research (COREQ) checklist is added as S1 File.

## Results

### Participants

Twenty adults from the general population and twenty adults with type 2 diabetes participated in the study. Demographic information for both groups is shown in Table 1.

**Table 1:** Demographic information.

	General Sample (n = 20)	Diabetes Sample (n = 20)
**Mean age (SD, range)**	42.65 years (14.47, 20–60)	64.30 years (15.30, 18–83)
**Women**	11 (55%)	12 (60%)
**Level of education**		
Primary school	0 (0%)	1 (5%)
Lower secondary education	1 (5%)	3 (15%)
Higher secondary education	13 (65%)	3 (15%)
College	3 (15%)	9 (45%)
University	4 (20%)	3 (15%)
**Marital status**		
Single	11 (55%)	3 (15%)
Married	8 (40%)	15 (75%)
Cohabiting	1 (5%)	0 (0%)
Widowhood	0 (0%)	2 (10%)
**Mean reported time since diagnosis (SD, range)**	NA	145.80 months (95.21, 6–324)

### Website Evaluation Questionnaire

Table 2 shows the results from the Website Evaluation Questionnaire for both the general sample and the individuals with type 2 diabetes. A fairly good score was given for each of the subscales by both groups.

**Table 2:** Results from the Website Evaluation Questionnaire.

Questions according to subscales	Scale Range	General Sample Mean (SD, range)	Diabetes Sample Mean (SD, range)
**Personal Relevance**	3–15	10.45 (2.16, 6–14)	10.85 (1.93, 6–14)
The information and advice provided by the website were of personal relevance to me	1–5	3.70 (0.73, 2–5)	3.65 (0.75, 2–5)
The website addressed my specific problems	1–5	3.10 (0.91, 1–4)	3.45 (0.83, 2–5)
The information and advice provided by the website were appropriate for me	1–5	3.65 (0.99, 1–5)	3.75 (0.72, 2–5)
**Engagement**	3–15	10.90 (2.97, 5–14)	11.35 (2.03, 8–15)
The website kept my attention	1–5	3.85 (0.99, 2–5)	4.05 (0.83, 2–5)
The website was engaging	1–5	3.45 (1.15, 1–5)	3.80 (0.70, 3–5)
I found the website enjoyable to use	1–5	3.60 (1.23, 1–5)	3.50 (1.00, 1–5)
**Goal-setting**	3–15	11.10 (2.02, 7–13)	11.65 (1.93, 7–15)
The website helped me to plan	1–5	3.60 (0.75, 2–4)	3.80 (0.83, 2–5)
The website helped me to think about my own behaviour	1–5	4.00 (0.79, 2–5)	4.00 (0.79, 2–5)
The website helped me to set goals regarding my PA / fruit intake/ vegetable intake	1–5	3.50 (1.15, 1–5)	3.85 (0.81, 2–5)

### Think aloud procedure

The content of the remarks from the general sample and the adults with type 2 diabetes was very similar. No group-specific themes were identified. Consequently, the results of both groups are discussed together. Table 3 gives an overview of the themes and shows the importance of each subtheme in the dataset by displaying the number of participants who endorsed the subtheme.

**Table 3:** Number of participants that addressed a subtheme.

Theme	General Sample (N = 20)	Diabetes Sample (N = 20)
**Knowledge**		
Perceptions regarding a healthy lifestyle	16 (80%)	13 (65%)
Perceptions regarding behaviour change	18 (90%)	15 (75%)
**Design of the intervention**		
General appreciation of the website	14 (70%)	14 (70%)
User friendliness	19 (95%)	20 (100%)
Time-efficiency	15 (75%)	18 (90%)
Lay-out	8 (40%)	4 (20%)
**Usefulness of the website**		
Opinion on the motivational value of the website	16 (80%)	10 (20%)
Opinion on the informative value of the website	10 (50%)	9 (45%)
Feelings of awareness elicited by the website	14 (70%)	10 (50%)
Personal relevance	15 (75%)	19 (95%)
Recommendations	10 (50%)	6 (30%)

#### Knowledge

Many participants stated their opinion about a healthy lifestyle (i.e. the positive effects of sufficient PA and the intake of fruit and vegetables). Some users agreed that having a healthy lifestyle has a positive effect on their physical and mental wellbeing, whereas others disagreed. Below some quotes of participants are provided. Underlined text indicates that the participant was reading the content from the website out aloud.

“Then I will have a smaller chance on getting diseases (cf. when eating more vegetables)… I hope so, but I don’t think it has much to do with it.”

(Female, 57)

“If I eat more fruit, then I will have a smaller chance on getting diseases. I do think that eating fruit might indeed be healthy.”

(Male, 23)

“My mental wellbeing will be better (cf. when being more physically active)… yeah, since I will exercise and lose weight.”

(Male, 49)

“My mental wellbeing will be better… those things (cf. mental wellbeing and eating fruit) are poorly related, it hardly affects your mental state.”

(Male, 78, type 2 diabetes)

Users often expressed their uncertainty about which actions actually are health-related.

“I make fresh soup. Does that also count?”

(Female, 83, type 2 diabetes)

“I once read that eating fruit after a meal is not healthy because of the fermentation process… I will practically never eat it after a meal.”

(Female, 64, type 2 diabetes)

“I often eat yogurt with fruit, but I guess that does not count?”

(Female, 48)

Some users shared their opinion about behaviour change and stated that creating action plans was unnecessary. In addition, users often believed that creating specific plans is difficult due to changes in their week schedules.

“I have a specific goal: completely disagree. I don’t have a plan! I think it’s weird to plan when you will eat fruit.”

(Female, 20)

“I don’t want an action plan, if I would like to eat it (cf. fruit) then I will do it, otherwise I won’t. It doesn’t have to be planned. I just eat when I want and what I want. No plan.”

(Male, 33)

“It’s difficult because everything depends from day to day and from week to week, because not every day or week is the same…”

(Female, 21)

“On how many days would you like to do your first activity? Three days a week. You do not know in advance, right? How can I plan this? I have a busy schedule, so it is unpredictable.”

(Male, 65, type 2 diabetes)

Users often stated that they did not need or want any social support when trying to have a healthy lifestyle. Consequently, many people were reluctant to send their plans to friends or family.

“Do you think other people will support you? What has that to do with it? I think it has nothing to do with each other.”

(Male, 23)

“I’m not going to send it, I’m doing this for myself”

(Female, 73, type 2 diabetes)

Furthermore, many users stated that they did not want to monitor their behaviour change.

“I’m not going to monitor this, I have my brain. I don’t want to monitor it. I’ll remember it, I don’t need a book or diary, no. I’m not going to do this, I’m not a child.”

(Female, 48)

“In my case, it’s hard to define whether I have reached my goal and if I reached it, then I don’t have to write it down. I don’t really see the point to start counting how many times I took the stairs. I would rather not keep track of this every day.”

(Male, 58, type 2 diabetes)

#### The design of the intervention

Participants also verbalized their general appreciation of the website and often described ‘My Plan’ as a questionnaire rather than an interactive tool that may help users with behaviour change.

“I don’t like to fill in questionnaires, I think it’s a waste of time and it’s not useful for me. I don’t think I would fill it in a second time.”

(Female, 20)

Almost all users had complaints regarding user-friendliness. Generally, there were four types of remarks. First, participants reported difficulties to fill out the questionnaires to assess PA, or intake of fruit or vegetables.

“I don’t really know how much vegetables weigh. A tomato, how much does it weigh? The recommendation is 300 grams, but I don’t know how I should envision 300 grams. And one vegetable weighs more than another one…”

(Female, 57)

Second, most participants found it hard to know how to answer questions, and doubted whether they were actually doing what the website requested them to do.

“The advice gives you a recapitulation and then you think “I didn’t answer the questions well”, maybe because I didn’t understand them, I don’t know.”

(Female, 69, type 2 diabetes)

Third, participants often reported difficulties with making the ‘if-then plan’.

“I find “if-then” difficult… It’s hard to put it into words, because well… I am an economist, but you can’t say three or five or seven, “if-then” just gives you too much freedom.”

(Male, 47)

Finally, the website offered users the possibility to send their plans to friends and family. However, users often did not know the email address of the persons they wanted to send it to.

“Would you like to show your plan to someone… yeah, why not. Email address, I don’t know by heart…”

(Male, 78, type 2 diabetes)

Many participants had remarks about the time-efficiency and stated that it took too much time to go through the website. Furthermore, filling out the questionnaires was often perceived as a waste of time.

“But it does take some time and you need to read it carefully too. And now we are lucky, but if you fill it out during the evening and the phone rings or at the office and someone walks in, you could be distracted, maybe it should be formulated more concisely.”

(Male, 58, type 2 diabetes)

“I think it’s—well not for me, because I have time—I think it takes too long and beats around the bush. It’s too long, I would have forgotten about it!”

(Female, 76, type 2 diabetes)

Participants who had remarks about the lay-out. They often disliked how the website looked, but appreciated the large font of the text.

“I really don’t find it an attractive website… Look at it. It is ugly as sin, it is not attractive, it is not interactive, it’s nothing… It’s for old people.”

(Female, 45)

“It’s good that the text has a large font, it invites you to read it, if it would be smaller, it would be difficult to read.”

(Male, 58, type 2 diabetes)

#### Usefulness of the website

Opinions about the motivational value of the website were mostly positive. Participants found it stimulating to make specific plans to be more physically active or to increase their intake of fruit or vegetables.

“I think it is nice that it (cf. the action plan) is well-defined, that you see that you can actually do more. The non-compulsory nature is gone”

(Male, 47)

“I think that making a plan helps you stick to the goal. If you really have a plan, I think you believe that you… otherwise you wouldn’t make it! So, I will do it.”

(Female, 31)

Nevertheless, participants who did not like the idea of making specific plans, were not motivated by the action planning component of the intervention.

“I don’t really think it’s necessary. I don’t think people will need the website to fill-in what they have done. I don’t need it. I also don’t think it will have a large influence on whether or not I will reach my goals, to eat fruit five times a week. That’s my opinion.”

(Female, 20)

“If this would go together with a dietician or a doctor or do it in a hospital, then I can imagine that it will be followed, but people on their own, they fill it in and forget about it.”

(Female, 76, type 2 diabetes)

Participants often verbalized their opinion about the informative value of the website and stated that they had learned something. These statements were mostly evoked when participants read the guidelines regarding PA and the intake of fruit and vegetables or when the website displayed their BMI.

“Two pieces of fruit a day, interesting!”

(Male, 56)

“Eating olives also counts? It’s strange that it’s a fruit, it surprises me!”

(Male, 60)

“I find this (cf. the action plan) informative”

(Male, 78)

Participants often expressed feelings of awareness when reading the guidelines about PA and the intake of fruit and vegetables, or when filling out the questionnaire regarding these behaviours.

“Damn, that’s not much. Horrible…”

(Female, 56, type 2 diabetes)

“I think it’s good that you are getting confronted with it and they highlight it and that they really inculcate it.”

(Male, 78, type 2 diabetes)

Perceptions regarding personal relevance were also stated. Although the website was created for adults in the general population, some participants felt like they did not belong to the target group and believed that the website was poorly tailored to their situation.

“It really depends on your age. You will not work in the garden when your back is starting to hurt. And if you are young, you will do more household chores, you will paint and perform renovations, but if it is done then it’s done.”

(Male, 72, type 2 diabetes)

“Physical activity during work… Well, first you need to have work!”

(Male, 57)

Participants with type 2 diabetes often stated that the suggestions for fruit did not fit within their dietary scheme.

“I shouldn’t eat apples, it’s all sugars they say… Mangos… I also don’t eat it.”

(Male, 78, type 2 diabetes)

While going through the website, some people shared their ideas about how ‘MyPlan 1.0’ could be adapted. Most recommendations involved creating a mobile application so people would be easily reminded to their goals.

“If you really want to do it well, then you have to monitor it. But I am not good at doing this, so an app would be really ideal to do this.”

(Male, 47)

“With an app you will be able to reach much more since you can send messages”

(Male, 60)

“It would be nice if the website showed graphs and if you could create your own tables.”

(Male, 49)

“I find it non-committal. I would rather send stuff on a daily basis. I would observe it more closely and react rapidly to people following a plan. You would get an automatic email saying “Have you eaten two pieces of fruit, yes or no?” and then they have to fill in which ones.”

(Female, 41, type 2 diabetes)

## Discussion

This study investigated the perspectives of users on a self-regulation-based eHealth tool labelled ‘MyPlan 1.0’ via a self-report questionnaire and a think aloud procedure. Noteworthy, the results of the Website Evaluation Questionnaire and the think aloud procedure indicate that participants from the general population and patients with type 2 diabetes had similar perceptions about health behaviours and ‘MyPlan 1.0’, except regarding the suggestions proposed by the website for fruit intake. This difference is appropriate. The norms for the amount of fruit during the day are different for people with type 2 diabetes than for the general population. Several participants with type 2 diabetes stated to avoid some types of fruit because of the potential impact upon their sugar level. Notwithstanding, research shows that the consumption of fresh fruit, when limiting the portions based on the choice of fruit and adjusting the insulin amounts to these choices, should be encouraged rather than discouraged in this population [10, 43, 44]. Hence, eHealth tools targeting adults with type 2 diabetes may also provide users with correct and up to date information about dietary guidelines. For example, when users with type 2 diabetes create a plan to replace unhealthy snacks by a piece of fruit the programme may explain why consuming snacks marketed specifically for people with type 2 diabetes should be discouraged and why it is beneficial to consume fresh fruit [44]. The same holds for guidelines regarding PA. For example, when users with type 2 diabetes create a plan to go hiking, information regarding the importance of regular blood glucose checks could be shown [45].

Nevertheless, it is promising that similar comments and remarks were stated by the sample with type 2 diabetes and the sample from the general population. This may indicate that there is a large overlap between the needs and expectations of both groups regarding an eHealth tool targeting a healthy lifestyle. If specific adaptations (e.g. adding information about the beneficial effects of fruit intake or about the importance of foot care) are made, ‘MyPlan 1.0’ has the potential to be a suitable tool for this clinical group as well. These findings may encourage other researchers to adapt existing interventions to new target populations as it may be more cost-effective and less stigmatizing than creating new condition-specific interventions.

According to the Website Evaluation Questionnaire users experienced ‘MyPlan 1.0’ as engaging. This finding is further corroborated by the think aloud procedure. Users reported that the website provided new information and raised awareness about the selected behavioural target. The results of the questionnaire furthermore show that the participants perceived the website as being personally relevant. However, the think aloud procedure revealed that personal relevance can still be improved: some participants stated that the website was poorly tailored to their specific situation. The effectiveness of offering a personalised approach by using tailored feedback and showing information (such as for example success stories) based on the user’s characteristics is well-established in research [18, 46–48] and should therefore be included as a standard element in all behaviour change interventions.

The results of the Website Evaluation Questionnaire indicate that users valued the website as helpful for goal-setting and self-assessment. This finding was also corroborated by the think aloud results: most of the users found it useful to make a tangible and concrete plan. Literature has shown that action planning can indeed be an effective technique for behaviour change [28, 49]. Nevertheless, action planning strategies should be offered in a way that is understandable for users. For example, although implementation intentions have shown to be effective in facilitating behaviour change [40, 50–52], users experienced great difficulties in creating their own implementation intentions. Consequently, implementation intentions should be accompanied by clear instructions and examples. Important to note however is that a fair amount of users reported that they did not see the point of making a specific plan for PA, fruit or vegetable intake. Likewise, some users did not understand why they should monitor their behaviour change or why they would seek for social support. This can be due to a lack of knowledge about how behavioural change is achieved. Therefore, future interventions should include the rationale for these components and give information to users about behaviour change and the self-regulation techniques that can be of aid. Even more, one could give the users more autonomy regarding which behaviour change techniques they want to apply in a specific situation.

An important finding of our study is that the Website Evaluation Questionnaire did not reveal the frustrations of users about the design of the website. This was better captured by the think aloud procedure: users described the tool rather as a long questionnaire than an intervention. They stated that it took too much time to go through the website and answer all the questions. Furthermore, they described that some questions were difficult and confusing. Although researchers are probably well aware that users prefer short and easy interventions [53], it remains an issue to specifically address. For example, Yardley and colleagues [35] also found that pages with extensive questions from their health-care website were often perceived as excessive by the users.

In order to overcome this problem, researchers should check with users whether interventions are sufficiently short and do not contain too much text, and adapt when deemed necessary. It is clear from our findings that extensive questionnaires should be avoided in online interventions, and kept to a minimum. Evidently, research on the efficacy of interventions may require multiple questionnaires at baseline, during and after intervention, and at follow-up moments. When this is the case, we recommend that participants are explicitly informed that these questionnaires are only for research purposes and are not part of the intervention. Another possibility to reduce the length of the online intervention is to only include the necessary components of behavioural change. Therefore, more research is warranted to identify the optimal combination of self-regulation techniques. Apart from frustrations regarding time efficiency, some users stated that they disliked the lay-out of ‘MyPlan 1.0’. This problem can be tackled by involving members of the target population early on in the developing process. Several authors have provided guidelines to adapt the lay-out of websites to specific target populations (for example see [54, 55]).

Finally, many users suggested that a mobile application would be useful for monitoring their behaviour and helping them remind about their goal. Indeed, self-monitoring has proven to be one of the most effective techniques for behaviour change [56]. A mobile application targeting a healthy lifestyle (mHealth) could indeed be a more convenient tool for self-monitoring and lead to a more sustained behaviour change by providing daily reminders and support. Furthermore, mHealth has shown to be promising in altering health behaviours [57, 58].

This study has several strengths. First, the think aloud method allows us to grasp immediate thoughts and reactions of users, not compromised by recall bias or researcher suggestions. Second, the perceptions of two different groups were investigated and found to be similar. As such, ‘MyPlan 1.0’ seems to have potential in clinical samples, such as patients with type 2 diabetes. Finally, the sample was heterogeneous and there was an equal distribution regarding sex, age and education. This study has also some limitations. First, a shortcoming of the think aloud method is that performing the intervention website interferes with thinking out aloud. Some users reported difficulties to simultaneously read text and to think aloud. Furthermore, users were more likely to elaborate on the things they found difficult or superfluous than highlighting the aspects they appreciated. Second, there are no norms available for the Website Evaluation Questionnaire. Therefore, the interpretation of low or high scores is arbitrary. Moreover, one should be cautious when interpreting results of the Website Evaluation Questionnaire, because users tend to give higher scores on the questionnaire compared to their actual experience [59]. Furthermore, although the calculated means of the Website Evaluation Questionnaire show that the intervention was perceived as engaging, relevant and helpful, a large range was found on all three subscales. This suggests that a certain group of users did not evaluate the intervention positively. Third, the fact that users went through the follow-up module immediately after filling out the start module compromises the ecological validity of the comments regarding the follow-up. Fourth, there is always the potential that results are biased. The–even unintended- influence of researchers (e.g. training, profession) may result in a confirmation bias [60]. However, we used a strict protocol for the think aloud procedure (e.g. the use of predefined prompts) and for the analyses of the data (e.g. the use of double coding). Furthermore, the interviewers were not involved in the development of ‘MyPlan 1.0’. Bias may also result from social desirability, especially because the researcher was sitting next to the participant [61]. In so doing, participants may have provided a more positive view about ‘MyPlan 1.0’ by neglecting the problems of the programme. However, we explicitly asked participants not to refrain from remarks, and stressed the constructive nature of these remarks for the further optimization of the programme. Finally, we did not assess specific characteristics of the clinical sample such as their treatment options or the presence of late-complications. This information might have given us more in-depth knowledge regarding our study population.

To conclude, this study used a think aloud procedure and a questionnaire to gain insight in the perceptions and preferences of the users of a self-regulation-based eHealth intervention. The presented study showed that the intervention, providing small adaptations, can also be used in tertiary prevention of type 2 diabetes. We thus argue that ‘MyPlan 1.0’ might be able to help adults with type 2 diabetes to adopt a healthier way of living which in turn will have a positive impact on the further development of their disease (i.e. better glycemic control [45] and a reduced risk of diabetic complications [62]). Furthermore, we found that there are still strong efforts needed to make eHealth interventions more convenient and less time-consuming. Finally, users’ ideas regarding health and behaviour change can form possible hindrances and should be taken into account. This study could be a first step in the development of an engaging eHealth intervention, but more research is needed to investigate how behaviour change techniques can be more conveniently implemented in eHealth. The use of mHealth can contribute to this process. Adaptations made to ‘MyPlan 1.0’ based on this study are described in S2 File. In further developmental phases, perspectives of users should again be explored in order to make constant improvements regarding personal relevance and user-friendliness.

## Supporting information

S1 File Completed COREQ checklist. (PDF)

S2 File Adaptations made in ‘MyPlan 2.0’. This file describes how ‘MyPlan 2.0’ was adapted based on users’ remarks and frustrations discussed in this study.
(PDF)

S3 File Think aloud Interviews. This file contains the transcribed interviews.
(ZIP)

S4 File Website Evaluation Questionnaire data. This file contains the data from the Website Evaluation Questionnaire.
(SAV)
