# Supplementary material for: Users’ thoughts and opinions about a self-regulation-based eHealth intervention targeting physical activity and the intake of fruit and vegetables: A qualitative study
Source: PLoS One. 2017 Dec 21;12(12):e0190020. doi: 10.1371/journal.pone.0190020 (PMC5739439; doi:10.1371/journal.pone.0190020)
Supplement: S1 File — (PDF) [file pone.0190020.s001.pdf]

Consolidated criteria for reporting qualitative research (COREQ): a 32-item checklist for interviews and focus groups

Domain 1: Research team and reflexivity

Personal characteristics

1. Interviewer/facilitator. Which author/s conducted the interview or focus group?

   LP, CVDM and two Master students (trained by LP and CVDM) conducted the interviews.

2. Credentials. What were the researcher's credentials? E.g. PhD, MD

   LP: PhD candidate in Health Sciences and Psychology
   CVDM: PhD candidate in Psychology and Health Sciences
   IDB: PhD in Psychology
   MV: PhD in Physical Education
   SS: MD and PhD in Clinical Physiology and Metabolism.
   GC: PhD in Psychology

3. Occupation. What was their occupation at the time of the study?

   LP and CVDM are PhD students performing research; MV is a postdoctoral researcher in health promotion, IDB is full professor in health promotion. GC is full professor in Health Psychology. SS is practicing endocrinologist at the university hospital and lecturer in endocrinology, diabetology and obesity.

4. Gender. Was the researcher male or female?

   LP, CVDM, IDB, MV and SS are female researchers, whereas GC is a male researcher.

5. Experience and training. What experience or training did the researcher have?

   LP has a Master's degree in Experimental and Theoretical Psychology.
   CVDM has a Master's degree in Clinical Psychology.
   IDB has a Master's degree in Clinical Psychology and a PhD in Health Psychology
   MV has a Master's degree and PhD in Physical Education and Movement Sciences
   SS is an MD and has a PhD in Clinical Physiology and Metabolism.
   GC has a Master's degree in Clinical psychology and a PhD in Psychology. He has extensive experience with empirical research on the psychosocial aspects of somatic illnesses.

Relationship with participants

6. Relationship established. Was a relationship established prior to study commencement?

No relationship with most of the participants was established before the commencement of the study. However, some of the participants were acquaintances of the interviewers.

7. Participant knowledge of the interviewer. What did the participants know about the researcher? e.g. personal goals, reasons for doing the research

The participants knew that the interviewers did not create 'MyPlan 1.0', but were trying to improve the programme.

8. Interviewer characteristics. What characteristics were reported about the interviewer/facilitator? e.g. Bias, assumptions, reasons and interests in the research topic

We report that the specific characteristics of the researchers (e.g. training, profession) might have an influence on data collection and analysis. Nevertheless, we created strict protocols to carry-out the interviews and to analyse the data to minimize bias. Also, none of the interviewers played a role in creating 'MyPlan 1.0'. We mention these features in the manuscript.

Domain 2: study design

Theoretical framework

9. Methodological orientation and Theory
What methodological orientation was stated to underpin the study? e.g. grounded theory, discourse analysis, ethnography, phenomenology, content analysis

Inductive thematic analysis was conducted in order to identify recurring patterns in participants' perceptions about 'MyPlan 1.0'.

Participant selection

10. Sampling. How were participants selected? e.g. purposive, convenience, consecutive, snowball

The sample from the general population was recruited via an available database, consisting of individuals who had expressed their interest to participate in studies of the Ghent Health Psychology Research Group via a website (http://www.healthpsychology.ugent.be/vrijwilligers), and via the snowball sampling technique. Participants with type 2 diabetes were recruited via advertisements distributed by the Diabetes Association Flanders and the Ghent University Hospital as well as by the snowball sampling technique.

11. Method of approach. How were participants approached? e.g. face-to-face, telephone, mail, email

Participants were recruited in different ways: face-to-face, telephone and email.

12. Sample size. How many participants were in the study?

In total twenty adults from the general population and twenty adults with type 2 diabetes participated in the study.

13. Non-participation. How many people refused to participate or dropped out? Reasons?

Six persons from the general population were not willing to participate. One person with type 2 diabetes could not participate because she did not have a computer. Consequently, another person with type 2 diabetes was recruited for the study.

Setting

14. Setting of data collection. Where was the data collected? e.g. home, clinic, workplace

The data were collected during home visits.

15. Presence of non-participants Was anyone else present besides the participants and researchers?

We asked participants to conduct the interview in a room where they would not be disturbed. However, in some cases we could not prevent that a family member occasionally disturbed the interview.

16. Description of sample. What are the important characteristics of the sample? e.g. demographic data

The demographic information of both samples is provided in table 1 of the manuscript.

Data collection

17. Interview guide. Were questions, prompts, guides provided by the authors? Was it pilot tested?

All questions and prompts are provided in the manuscript. These prompts were not pilot tested, but were based upon previous research by Yardley and colleagues (2010).

18. Repeat interviews. Were repeat interviews carried out? If yes, how many?

There were no repeat interviews carried out.

19. Audio/visual recording. Did the research use audio or visual recording to collect the data?

All verbalizations were voice-recorded and the computer screen was filmed.

20. Field notes. Were field notes made during and/or after the interview or focus group?

Yes, each interviewer made field notes of the interview.

21. Duration. What was the duration of the interviews or focus group?

The duration of a home visit was approximately 75 minutes.

22. Data saturation Was data saturation discussed?

Yes. After identifying the different themes the transcripts containing all data were read again with these themes in mind. This was done to check whether the data was well captured by the themes.

23. Transcripts returned. Were transcripts returned to participants for comment and/or correction?

No.

Domain 3: analysis and findings

Data analysis

24. Number of data coders. How many data coders coded the data?

    Two data coders (LP and CVDM) coded the data.

25. Description of the coding tree. Did authors provide a description of the coding tree?

    Yes. This is provided in table 3.

26. Derivation of themes. Were themes identified in advance or derived from the data?
    The themes were derived from the data.

27. Software. What software, if applicable, was used to manage the data?

    The qualitative data analysis software nVivo 11 (QSR International Pty. Ltd. Version 11, 2015) was used to manage the data.

28. Participant checking. Did participants provide feedback on the findings?

    No.

Reporting

29. Quotations presented Were participant quotations presented to illustrate the themes / findings? Was each quotation identified? e.g. participant number

    Yes.

30. Data and findings consistent. Was there consistency between the data presented and the findings?

    Yes.

31. Clarity of major themes. Were major themes clearly presented in the findings?

    Yes. Table 3 gives an overview of the number of participants that addressed each theme and subtheme.

32. Clarity of minor themes. Is there a description of diverse cases or discussion of minor themes?

    Yes.
