# Supplementary material for: Users’ thoughts and opinions about a self-regulation-based eHealth intervention targeting physical activity and the intake of fruit and vegetables: A qualitative study
Source: PLoS One. 2017 Dec 21;12(12):e0190020. doi: 10.1371/journal.pone.0190020 (PMC5739439; doi:10.1371/journal.pone.0190020)
Supplement: S2 File — This file describes how ‘MyPlan 2.0’ was adapted based on users’ remarks and frustrations discussed in this study. (PDF) [file pone.0190020.s002.pdf]

Adaptations made in 'MyPlan 2.0'

The table below describes the problems and frustrations regarding 'MyPlan 1.0' discussed by the participants and the adaptations that were made in 'MyPlan 2.0' to overcome these issues.

Problems in 'MyPlan 1.0'	Adaptations made in 'MyPlan 2.0'
Participants stated that 'MyPlan 1.0' was **poorly tailored** to their situation.	'MyPlan 2.0' offers a more personalised approach. For example, success stories based on the participant's age and gender were added to the intervention. (see fig. 1)
Participants did not understand **the usefulness of several behaviour change techniques** implemented in 'MyPlan 1.0'.	'MyPlan 2.0' gives a rationale for each proposed behaviour change technique. For example, the website explains why coping planning is important and how it can help users in the behaviour change process. (see fig. 2)
Participants stated that 'MyPlan 1.0' **was not time-efficient** and described the programme as a long questionnaire rather than an intervention.	Questions solely asked for research purposes were deleted. Furthermore, information is given via quizzes instead of lengthy text pages. (see fig. 3) Going through one session now only requires 10 minutes of the users' time.
Participants stated that they did not like the **lay-out** of the website.	'MyPlan 2.0' has a different lay-out than 'MyPlan 1.0'. Lengthy text pages were deleted and more images were added. (see fig. 1-3)
Participants stated that **a mobile application** would be useful in their behaviour change process.	A mobile application accompanying the website was created. Via this application users can monitor their behaviour (see fig. 4), do quizzes (see fig. 5), revise and adapt their plan (see fig. 6) and find solutions for hindrances (see fig. 7).

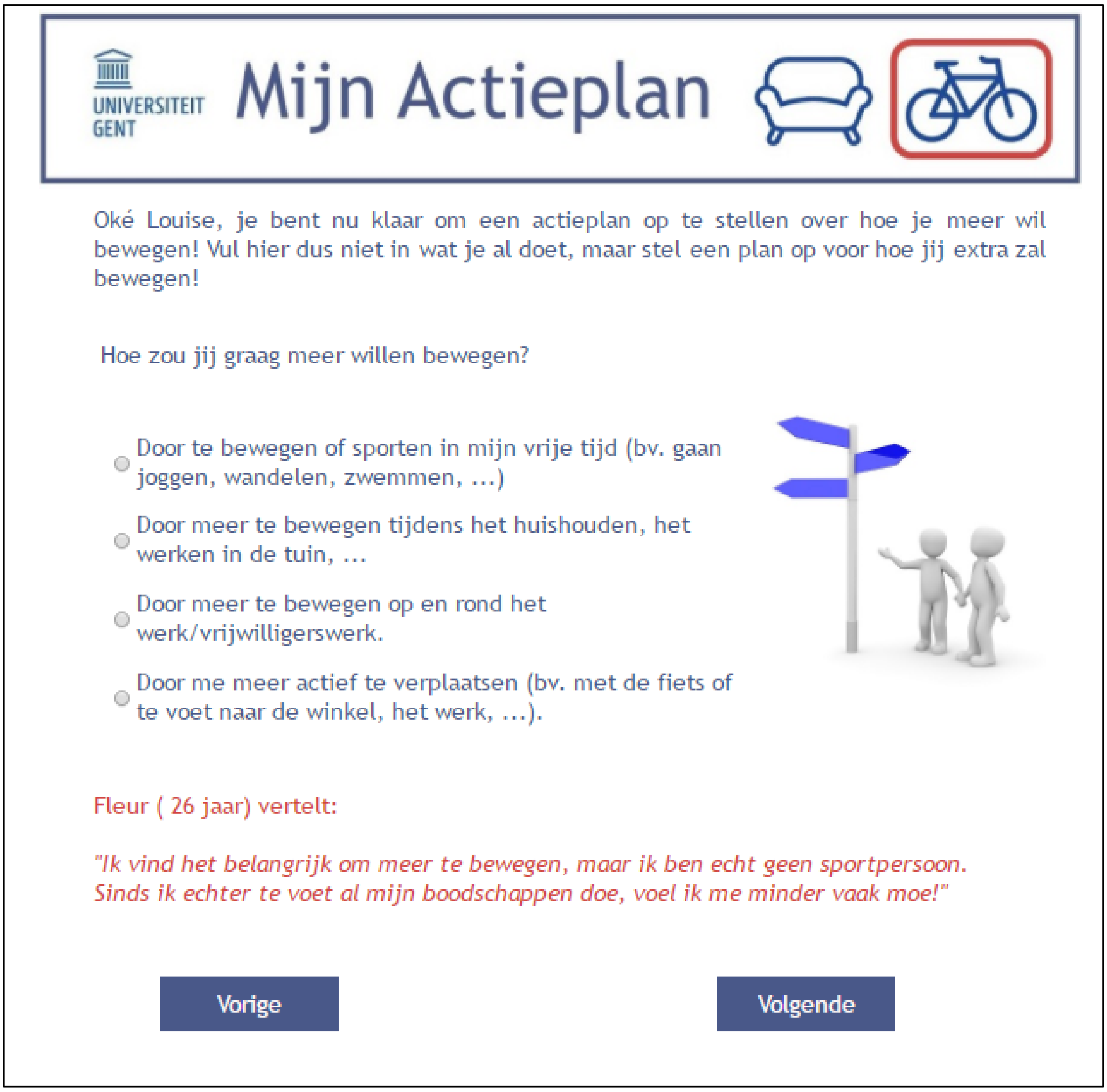

Figure 1. Success stories (website)

The text in red is a success story.

Translation: “Fleur (26 years old) tells: I consider it important to be more physically active, but I am not a sporty person. Since I do all my groceries by foot, I feel less tired during the day!”.

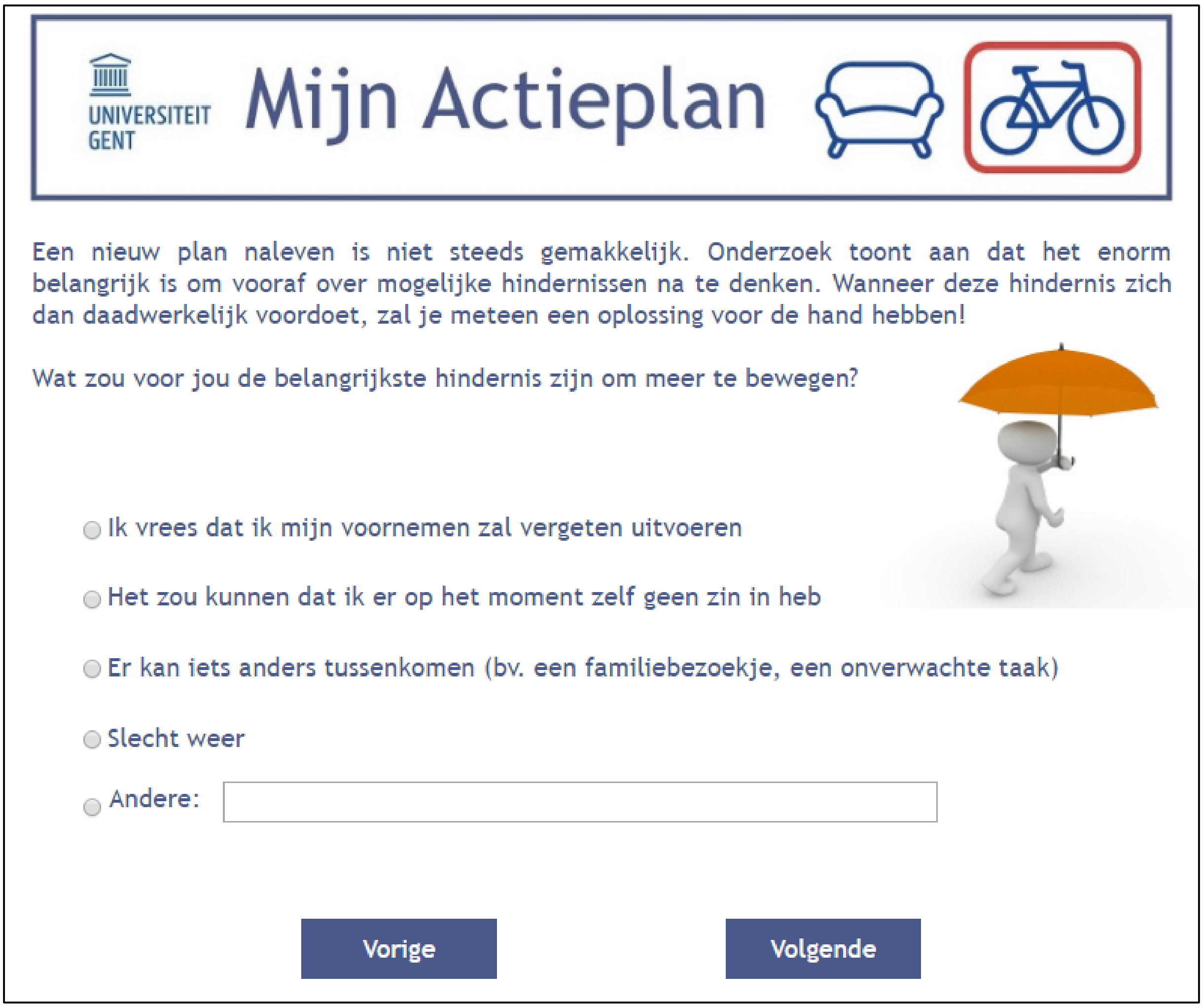

Figure 2. Rationale for coping planning (website)

Translation: “Living up to a new plan is not always easy. Research shows that it is really important to consider potential hindrances in advance. When this hindrance appears, you will have an immediate solution at hand!”

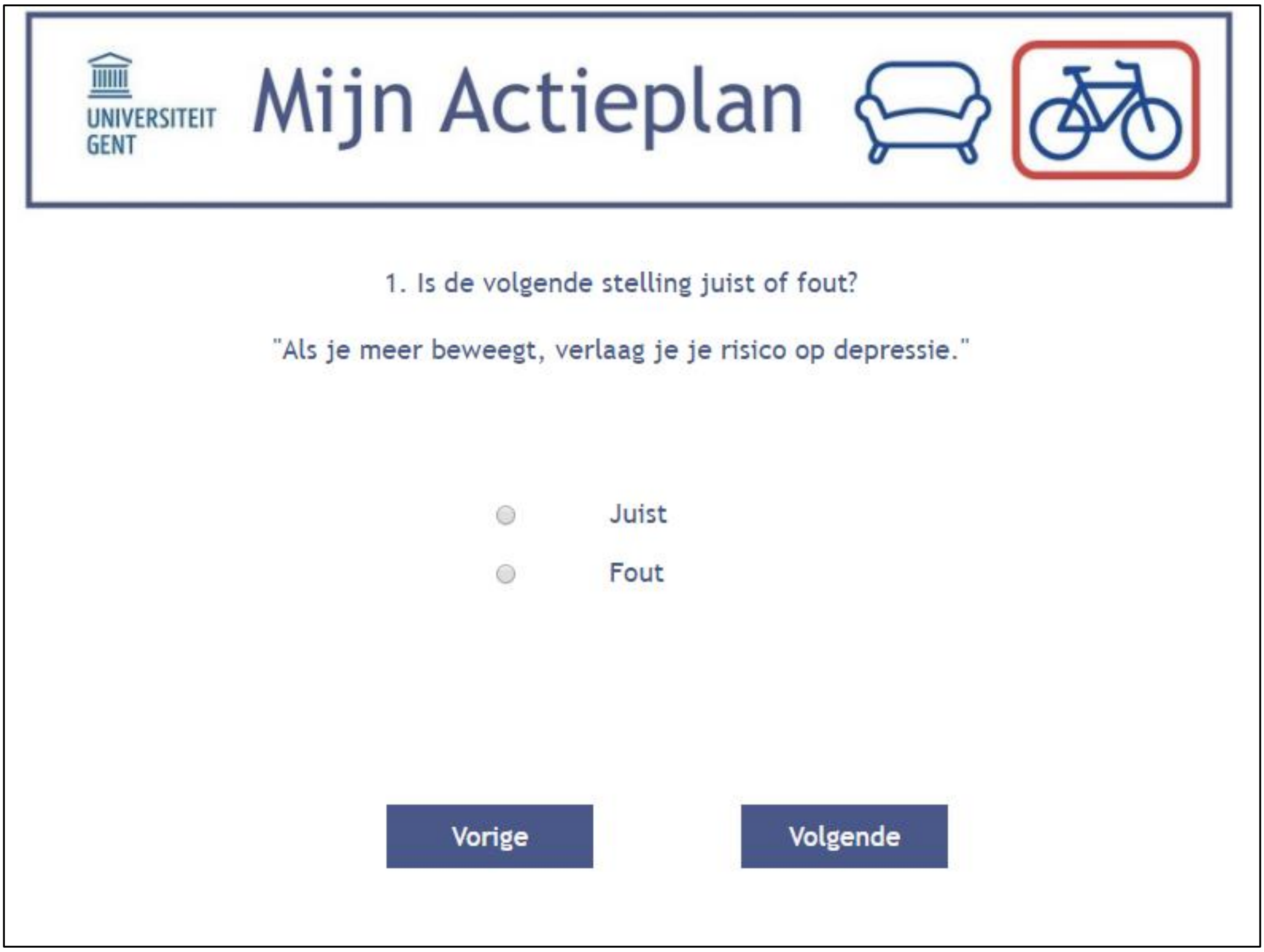

Figure 3. Example of a quiz question (website)

Translation: “Is the following statement true or false? I you are more physically active, you have a lower chance on developing a depression.”

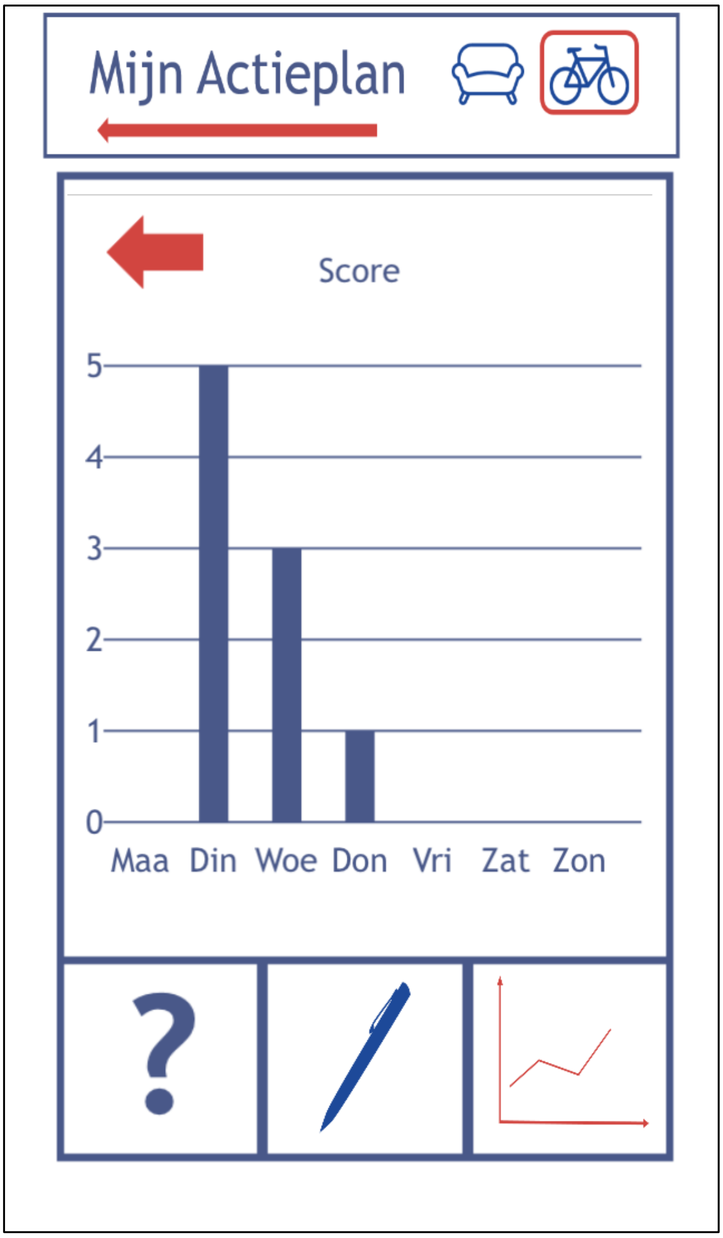

Figure 4. Monitoring health behaviours (mobile application)

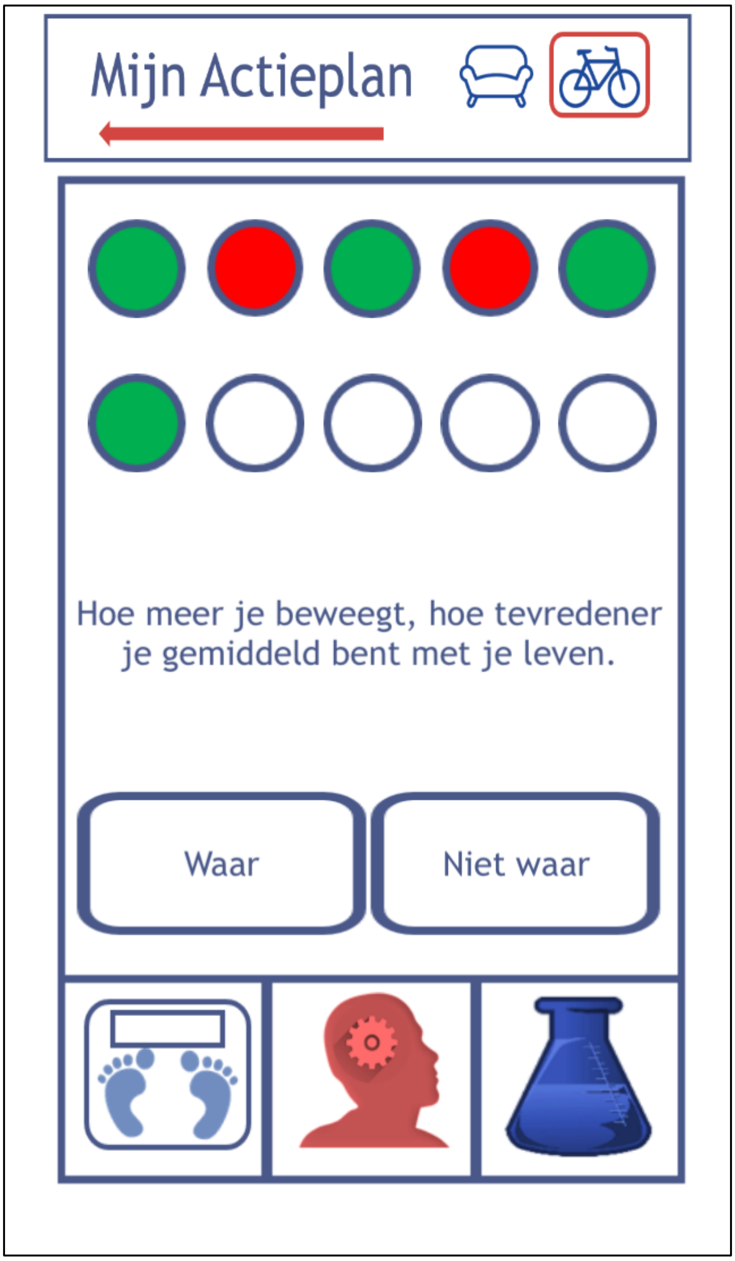

Figure 5. Quizzes (mobile application)

Translation: “In general, the more people are physically active, the more happy they are with their lives.” Blue boxes: “True” and “False”.

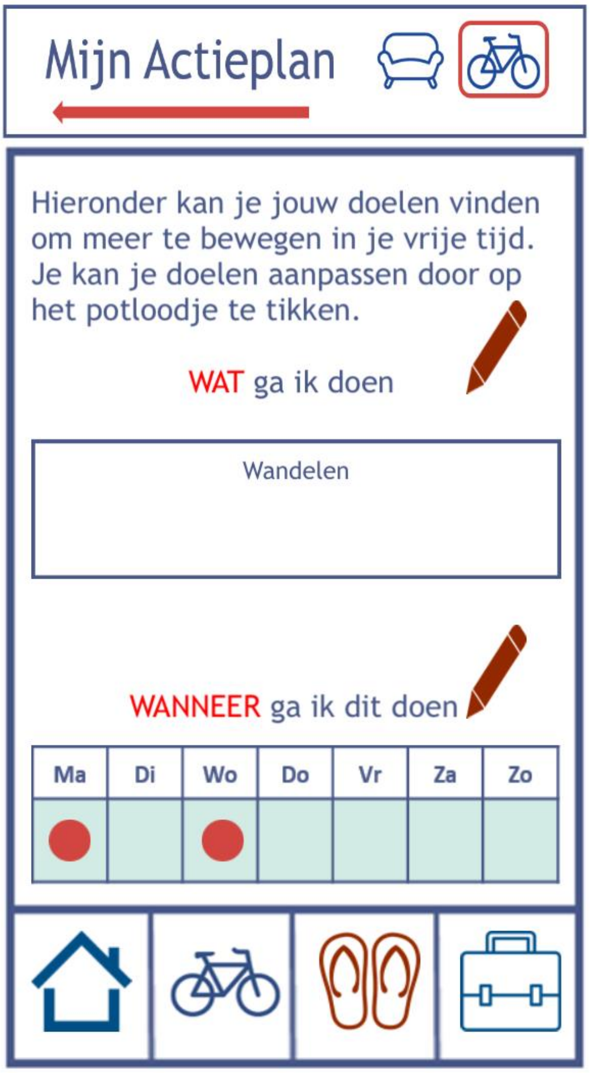

Figure 6. Revising and adapting plans (mobile application)

Translation: “Below you can find your goals to be more physically active during leisure time. You can adapt your goals by tapping on the pencil icon.”

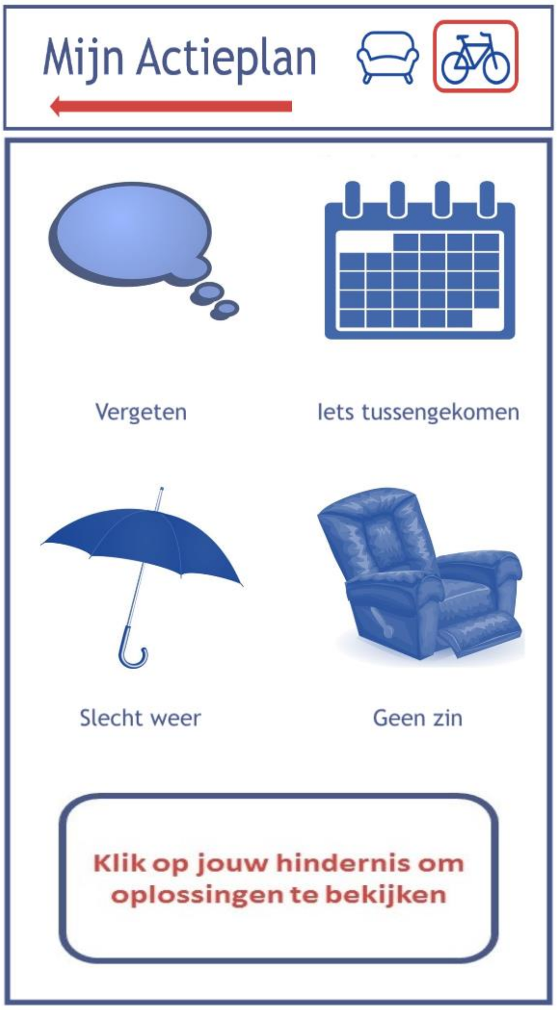

Figure 7. Coping planning (mobile application)

Translation: “Tap on a hindrance to find solutions”.
