# Supplementary material for: Users’ thoughts and opinions about a self-regulation-based eHealth intervention targeting physical activity and the intake of fruit and vegetables: A qualitative study
Source: PLoS One. 2017 Dec 21;12(12):e0190020. doi: 10.1371/journal.pone.0190020 (PMC5739439; doi:10.1371/journal.pone.0190020)
Supplement: S3 File — This file contains the transcribed interviews. (ZIP) [file pone.0190020.s003.zip › general_population/TA1BENZ.docx]

**Code filmpjes:**

| Deel interventie | Minuten | Transcript |
| --- | --- | --- |
| DEEL 1  VRAGENLIJST | 0-10 | **Je mag de website doorlopen en zeggen wat in je opkomt.** Hoeveel beweeg je en is dat genoeg denk je. Niet genoeg. Hoeveel, dat is zeer wisselend. Eet je voldoende groeten op een dag. Hmm .. ja. Dat denk ik wel. *(vult zijn code in)* geef je leeftijd op. Verder. Bedankt. Je paswoord is .. ja goed. 300 gram groenten per dag, 2 porties fruit. Is fruitsap ook goed? Anders zit ik er wel over, haha. **Vers fruitsap dan?** Neen neen. **Ah neen, dat telt niet mee dan!** 30 minuten bewegen .. ja. Ik moet dat niet luid lezen he? **Neen dat hoeft niet.** Moet ik 1 van de drie kiezen, ah ja dat ik wil verbeteren .. fruit! Daar heb ik het meeste aan. Welkom. Je hebt gekozen voor de module fruit. *(leest in stilte verder).* Hoe heet je. Heb je al een andere module ingevuld, neen. Wat is je email adres. GSM nummer. **Dat is niet verplicht.** Wat is je leeftijd geslacht hoogste diploma .. hup. Hoeveel beweeg je. Ik denk eeeeh.. hm. Vier dagen in de week. Hoeveel heb je de voorbije week gegeten, aah vanalles! Pakt, een stuk of vier appels. Dat is voor alles he, niet alleen fruitsla he toch? **Neen het is voor elk apart.** Frambozen .. ’t is just! Ik heb 2 dagen aardbeien gegeten, moet k terug keren? Ja he. Een stuk of vier vijf zeker. Abrikozen dat heb ik niet gegeten, pruimen ook niet neen. Kersen krieken niet. Nectarine.. mango’s, dat is de voorbije zeven dagen he. Hup. Meloen aah, ik heb dat gegeten. Oke. Druiven. Hoeveel fruit denk je dat je eet, eigenlijk niet veel. Ben je van plan meer te eten, misschien wel misschien niet. Gemakkelijk. Het is te zien wie dat er naar de winkel is gegaan. Zal ik mij mentaal beter voelen, jah. Zullen anderen mij steunen.. pfff. **Waarom blaas je bij die vraag?** Dat is te zien hoe dat de anderen – ik ga hen niet zeggen dat ik meer fruit ga eten. Meer fruit kan eten, ja. Meer fruit in onmogelijke situaties, hmm hmm .. dat is wat anders. **Als er dingen bij je opkomen mag je die zeker zeggen he.** Ik heb het er moeilijk mee om voor mezelf doelen te stellen om meer fruit te eten. Neen. Ik heb er moeite mee om plannen te maken om deze doelen te behalen, neen. Ik hou mijn vooruitgang bij om na te gaan of ik meer fruit eet, euuuh, waarschijnlijk niet. Als ik dat doe, dat duurt een week en na een week doe ik dat al niet meer. Ik ga niet opschrijven wat voor appels ik eet he. Ik heb een duidelijk plan voor wanneer ik meer fruit ga eten, hmja, dat zou wel kunnen! Wat ik soms doe is naar de winkel gaan ‘ smiddags, en als we een appel of vier kopen en dan eet ik dat in de voormiddag, dat gebeurt wel. Voor hetzelfde geldt is dat yoghurt of boterkoeken haha! Er zit geen structuur in. Maar ik zou het eigenlijk wel kunnen. Ik zou dat kunnen. ik heb een duidelijk plan voor wat ik ga doen als iets tussen mijn plan komt te staan, neen. In situaties waarin het moeilijk is.. ook niet. Fruit eten, daar komt er niets tussen he. Als ik dat wil ga ik dat doen. En als een andere zegt je mag geen fruiten eten, dat pff. |
| DEEL 1 ADVIES | 10-11.20 | Je geeft aan dat je 4 dagen fruit eet. In die dagen eet je gemiddeld zoveel, het is goed dat je fruit eet. Je moet gemiddeld 2 porties per dag eten. Om je een idee te geven … ja. Dat zou wel lukken, ik ga meedoen. Oké. |
| DEEL 1 OPSTELLEN ACTIEPLAN | 11:20 | (leest de vragen heel stil voor) ik ben gemotiveerd. Je kan ook zelf een methode bedenken en noteren bij anderen. Fruit op een boterham eten .. hmmm. Fruit als tussendoortje dat is goed. Dat ander, ahja, telt dat ook mee appelmoes? **Ja.** Ah want we eten toch wel 2x per week appelmoes. En peren stoven doen we ook, dat is juist. Dat zit er niet bij he. Hup. We gaan het erbij zetten. Fruit in salades neen dat ga ik niet rap eten. Aperitief, dat doe ik ook veel he! Olijven eten, telt dat ook? **Ja.** Dat is raar dat dat een fruit is, daar verschiet ik van! Dat doe ik wel. Fruit op voorhand bereiden, neen. Goed. **Wat vind je van dat onderdeel als je het zo ziet?** Het zitten goeie dingen bij, maar er zitten ook dingen bij die ik al doe eigenlijk. Ik denk dat ik mijn eigen heb onderschat bij het fruit eten. Nu dat ik het hier allemaal lees .. bij mij is het hup een stuk fruit eten en dat gestructureerd doen, dan ga je dat veel meer in je handen hebben. Want voor hetzelfde geld dan eet ik een week geen fruit hoor. Maar als je daar een structuur in brengt, dat zou wel een structuur kunnen geven. 6 dagen. Ik ga altijd wel eens een dag tussenlaten. Ik wil vooral fruit eten thuis, op het werk. Op mijn fiets, dat doe ik wel he. Als ik ga koersen een stuk fruit meenemen. Of dat ik stop aan een winkel en appels koop, dat doe ik wel. ‘op mijn fiets’ dat lijkt zo raar hé. Wanneer wil je vooral fruit eten? Tijdens het ontbijt. Als tussendoortje. ’s Avonds niet. ‘als-dan’ plan, dan kan je het volgende doen. Als ik een tussendoortje zal eten, zal ik vooreerst fruit eten. Goed. **Wat komt er zo bij je op bij dat onderdeel?** Dat dat goed is , maarja weet je wat, ik eet zo geen tussendoortjes eigenlijk. Ik werk altijd 7 uur aan een stuk. Die tussendoortjes dat is zo niet gestructureerd, dat is doorwerken eigenlijk. Ik ga om een glas water of eens naar de keuken, of om koffie maar vooral om me eens te verplaatsen, of ik ga gaan printen om niet te blijven zitten, en dan zou ik eigenlijk wel fruit kunnen eten, dat is wel mogelijk. In de keuken staat er wel altijd vanalles om te snoepen dat is wel verleidelijk. Maar ja. Het is dat. Dat is het meest praktische he, dat ik tussendoor fruit eet. Maar ik doe het eigenlijk wel, maar niet gestructureerd. **Moet er hier nog iets bij?** Dat mag je zelf beslissen. Fruitsap mengen met water, dat doe ik wel echt veel. Telt dat? **Neen.** Je bent wel een strenge he jij! **Ja, maar het echt om fruit.** Fruit na het eten, dat is iets dat wij niet doen. Hup, die tussendoortjes zijn het meest haalbaar. **En nu moet je eigenlijk ‘vanaf vandaag’ aanklikken anders blokkeert het programma.** **Normaal gezien mag je kiezen wat je aangeeft.** Ja. Fruit bij de warme maaltijd eten, dat is iets dat we veel doen. Als aperitiefhapje, dat is ook iets dat ik veel doe. Tot wanneer ga ik dat doen, vanaf 5 .. |
| DEEL 1 ACTIEPLAN | 18:51 | meer dan 2 porties fruit eten. Te vermijden.. **wat komt er dan op bij jou?** We zijn de Franse Revolutie al lang voorbij en mijn .. wordt niet bepaald door zoiets he. Om te vermageren, ik heb niet gezegd dat ik wilt vermageren he. Weet je wat het is, dat is niet nodig want nu begint het wielerseizoen he. Ik ga van de zomer vooral veel vermageren door te koersen, in de plaats van suiker een stuk fruit eten. Ongezonde tussendoortjes, maar ik eet bijna geen tussendoortjes. Wat aardbeien. Het beste wat je kunt doen is waar ik werk dat eens opschrijven. Zo een tabelleke, dat ik daar elke dag naar kan kijken. Of een kalender. Zo een groot papier op mijn bureau. **Ja een kalender is dat eigenlijk he**. Ja voila. Al ist dat je dat kunt he op je smartphone, dat je verwittigd wordt dat je nog geen fruit gegeten hebt. **Zo een app bedoel je?** Dat zo interessant zijn. Dat denk ik ook. Een app voor de smartphone. Dat is het. Een app voor het fruit. Ik ken geen emailadressen. Volgende. Je actieplan is klaar, is het al gedaan? Versturen. Oei. Ze gaan dat naar hier sturen? Dat gaat daar toekomen, dit is de PC van mijn werk. |
| DEEL 1 REST |  |  |
| DEEL 2 VRAGENLIJST |  |  |
| DEEL 2 AANPASSEN ACTIEPLAN |  |  |
| DEEL 2 REST | (volledig 2^e^ fragment) | Goed? Oke nog een keer email adres. Ja. Ja. 6 dagen. Ja hé? Slaatjes fruit, nee he. Ik heb zeker een appel of vijf gegeten. Vijf. Ja. Aardbeien dat ga ik zeker een stuk of vier keer gegeten hebben. Abrikozen, neen. Mandarijntjes .. mango’s. druiven .. fruitmoes, ah appelmoes! Een keer. Nul. Inderdaad. Ja. Ja. Hetzelfde. Goed. Ja. Wanneer, dat staat al ingevuld kan dat? **Ja vandaag zijn we de 5^e^.** veel succes en dan ga ik een mailtje krijgen? **Normaal gezien komt er dan nog een email na een maand maar dat gaan we niet doen, maar je gaat die wel krijgen dus als je wilt kan je die dan bekijken.** **Dat was het eigenlijk voor de website, nu zou ik willen vragen welke zaken dat je eigenlijk goed vond?** Als je echt je best doet ga je iets kunnen bereiken, de follow-up hé. Met een app ga je veel meer kunnen bereiken met berichtjes te sturen enzo. En een beloning geven ofzo. **En hoe zie je dat dan?** Tussen de eerste duizend verloot je een reis naar een of ander fruitland ofzoiets. Iets dat eraan gekoppeld is. **Wordt dat dan niet iets te commercieel?** We leven wel in een commerciële wereld. **En wat zat er in dat je goed vond?** Euh .. begeleiden is belangrijk, moest je hier niet zitten zou ik het misschien niet helemaal ingevuld hebben. **Normaal gezien zit ik er wel niet bij, stel dat je de website alleen had** **gedaan.** Waar we naartoe moeten is dat er huisartsen zijn waar er ook een psycholoog is die dat afneemt en dan gaan die dat veel beter kunnen volgen dan artsen die daar niet in geïnteresseerd zijn of geen tijd voor vinden. Diabetes volgen enzo, gezondheidspsychologie. 1 keer per maand ofzo. Daar je voor pleiten. **En het programma op zich?** Het programma op zich is goed, persoonlijk vind ik het niet heel aantrekkelijk. K vind het niet flashy ofzo of je moet met geweren schieten naar een stuk banaan, ik zeg maar iets. Je moet dat echt opbouwen als iemand vooruit wil, het is moeilijk om te zeggen. **Maar je mag dat zeggen hoor.** **Er is nog een vragenlijstje over het programma zelf zoals je nu aan het vertellen bent.** |
