# Supplementary material for: Users’ thoughts and opinions about a self-regulation-based eHealth intervention targeting physical activity and the intake of fruit and vegetables: A qualitative study
Source: PLoS One. 2017 Dec 21;12(12):e0190020. doi: 10.1371/journal.pone.0190020 (PMC5739439; doi:10.1371/journal.pone.0190020)
Supplement: S3 File — This file contains the transcribed interviews. (ZIP) [file pone.0190020.s003.zip › general_population/TA1BEON.docx]

**Code filmpjes:**

| Deel interventie | Minuten | Transcript |
| --- | --- | --- |
| DEEL 1  VRAGENLIJST | 0-10 | **Je mag zeggen wat er in je opkomt. Je mag het doorlopen.** Het gaat over of ik genoeg fruit eet. **Sommige mensen lezen luidop als dat gemakkelijker is.** Nee cava. (vult toegangscode in). Leeftijd, 33. Eet je voldoende porties fruit per dag en is dat genoeg denk je. Fruit ja, ik drink wel lemons, ik eet wel fruit maar ik overdrijf niet, niet te veel, niet te weinig. K eet wel veel groenten en k beweeg sowieso wel genoeg met men fiets. Hier en daar wat trainen. Dat is safe. Hmm. We gaan eens fruit nemen om te beginnen. Hoeveel weeg je, 70. Lengte 1m86. Geen suikerziekte. Geen dieet. Citroen is ook fruit eten? **Neen neen**. Kheb in een periode massaal veel fruit gegeten – **het gaat hier echt om de laatste week. Vanaf vorige week donderdag**. Ik ga dat aanduiden. Ananas is een. Perzik dan moet ik 2 nemen. Wat is de bedoeling. Hoeveel heb je gegeten van deze fruitsoorten. 100, wat wilt dat dan zeggen? **Dat je heel veel porties ervan hebt gegeten.** Dat is zelf niet meer gezond! Als je dit soort niet hebt gegeten geef dan 0, dus dat wil zeggen dat dat een soort fruit is en dan is ja 1 en nul neen gewoon en de andere cijfers van geen belang. Dat zou wel wat duidelijker kunnen vind ik. **Je moet het zien als een portie.** Bessen, frambozen, niet echt. Aardbeien, tis de periode nog niet. Niet de voorbije weken. Kiwi’s neen. Nectarine, neen. Tros druiven, neen. Zit boordevol pesticiden. Fruit uit blik. Niet de voorbije week. Wat is dat die eetlepels? **Dat is een hoeveelheid.** **Als standaardportie wordt 125g fruit gerekend.** Dus een pot appelmoes, ik pak een eetlepel eruit? **Neen dat is meer voor die ananas van daarjuist.** |
|  | 10-12 | Over die voorbije week enkel maar? Weinig. Anders zou ik iedere dag meer fruit eten. Dat is wreed he dadde, zouden andere mij steunen.. wat is dat nu weer. Als ze willen dat ik gezond ben ja, als ze willen dat ik niet gezond ben nee. Ik zal hier op klikken. Fout. **Waarom blaas je?** Denk je dat je meer fruit kan eten ook als je problemen en zorgen hebt, ‘kheb zorgen ik ga geen fruit eten’ dat heeft daar niets mee te maken. Dat is gewoon voeding. Het zou zelf beter zijt om fruit te eten, dan zit je met goede vibes. Zoals citroen dat is goed tegen depressie. Ik wil geen actieplan, als ik zin heb ga ik dat eten en anders niet he wat is dat nu. Dat moet niet zo gepland zijn toch. Ik eet gewoon waar ik goesting in heb, waar je op dat moment zin in hebt. Geen plan. Dat is allemaal niet safe he ja. Buiten het plan in het algemeen dat je fruit moet eten, daarvoor moet je geen plan hebben he. |
| DEEL 1 ADVIES | 12-16:15 | Persoonlijk fruitdoel *(lacht)*. Eens een week fruit eten, een week geen. Een week veel fruit. **Voor het verder verloop zou ik toch willen dat je eens op ‘ja’ drukt, je kan maar eens proberen he.** Nu? Oke cava. **Misschien helpt het.** Ja. |
| DEEL 1 OPSTELLEN ACTIEPLAN | 16:15 | Een beetje een gewoonte van maken. Moet ik dat erbij zetten? **Als je dat wilt.** **Je mag nog altijd positieve en negatieve zaken zeggen.** Ja ja. **Eet jij elke dag fruit?** **Neen.** Dat is toch te veel he, iedere dag 2 porties. **Dat is wel de aanbevolen norm**. Je eet ook groenten, daar zitten ook goeie vibes in; ik denk 7 op 7 fruit eten.. **als je drie wilt aanduiden dan moet je dat doen.** Het is eigenlijk best dat je het … wat moet ik hier doen ook als dan vibe checken. **Je moet dat maken voor jezelf.** Dat is hatelijk dat ik dat moet checken. **Waarom.** Kweetniet ja, ik vind dat echt dwaas. Dat gaat mij niet helpen. Ik weet wel wat de bedoeling is dat je weet hoe je in de realiteit je gedrag zou aanpassen. Maar wat willen ze dat ik zeg ‘als ik een koekje wil eten dan zal ik in de plaats daarvan fruit eten’ alleja. Mag dat erbij? **Als jij dat wilt.** **Nu moet je de datum van vandaag aanklikken anders loopt mijn programma fout.** **Wat vond je van dat onderdeel?** Lastig. Voor mij he. Ik heb meer aan gewoon informatie, zoals alle soorten fruiten en dat soort fruit is goed voor dat en dat en die vitamines zitten daarin, daar heb ik echt veel meer aan dan zo een actieplan, dat is echt een beetje kinderachtig voor mij zo een plannetje opstellen. Snap je. Ik heb dan meer aan een informatieve site waar je naartoe kan gaan en dan lezen. Of heb je klachten van dat soort dan is het goed om dat te eten enzovoort. Dat vind ik beter. Ik denk niet dat dat mij zoveel gaat helpen. |
| DEEL 1 ACTIEPLAN | 24:20 | En nu? **Je moet het niet versturen.** |
| DEEL 2 VRAGENLIJST | (2^e^ fragment) | Staan er overal rode sterretjes? Dat je het moet invullen? **Ja.** **Je telefoon nummer moet je niet invullen.** Het is weeral die fruit vibes? **Ja je bent begonnen met fruit dus je doet verder met fruit he. Alles gaat verder op fruit. Moest je groenten of beweging nog doen dan kan je dat later nog doen**. **Je moet je inbeelden dat je een week verder bent.** |
| DEEL 2 AANPASSEN ACTIEPLAN | 3:25 – 4:04 + 3^e^ fragment | Nice. **Je mag nog altijd zeggen wat je positief of negatief vindt**. Onverschillig. Yess. Validator. Weeral dat. Schaaltje fruitsla. 3 ananas. Niet zoveel bessen. Aardbeien, k eet al elk jaar voor mijn verjaardag aardbeientaart. Het is bijna april, de periode van aardbeien. **Wat denk je zo bij dat onderdeel?** Pff .. ik weet het niet. Wat moet ik daarvan denken. **Misschien is er iets dat bij je opkomt.** Het is niet leuk, misschien wat foto’s van fruit op plakken. Het is boring. |
| DEEL 2 REST | 3:45 – 5:30 | **Dat was het einde**. Yess! I**k heb juist nog een aantal algemene vragen, in het algemeen bij het overlopen van het programma, welke zaken vond je precies goed?** Het is misschien goed wel dat ze je vragen of je zelf er een keer goed aan moet denken of je wel genoeg fruit eet, dat is misschien niet slecht. Dat doet je wel denken of je genoeg fruit eet. **Zijn er dingen die je echt slecht vond?** Dat laatste met die cijfertjes, dat is een beetje saai wel. Dat zou je wel beter kunnen aankleden. En in algemeen op de site meer foto’s, van nice fruit zodat het je zin geeft om fruit te eten. Vers gesneden sinaasappel ofzo. Dat het je zin geeft om fruit t eten. |
