# Supplementary material for: Users’ thoughts and opinions about a self-regulation-based eHealth intervention targeting physical activity and the intake of fruit and vegetables: A qualitative study
Source: PLoS One. 2017 Dec 21;12(12):e0190020. doi: 10.1371/journal.pone.0190020 (PMC5739439; doi:10.1371/journal.pone.0190020)
Supplement: S3 File — This file contains the transcribed interviews. (ZIP) [file pone.0190020.s003.zip › general_population/TA1BEOR.docx]

**Code filmpjes:**

| Deel interventie | Minuten | Transcript |
| --- | --- | --- |
| DEEL 1  VRAGENLIJST | 0-10 | *(invullen van de gegevens).* Bestaat uit drie modules. Uhu. Ja. Ow. Dat weet ik niet vanbuiten. **Je mag zeggen wat er in je opkomt ondertussen.** Over de website zelf? Het is wel raar dat ik 2x mijn leeftijd moet invullen. Het hoogste diploma. Ja. Hoeveel weeg je, 72. Lengte. Ja. Diabetes en suikerziekte neen. Dieet neen. Hoeveel dagen heb je de voorbije week groenten gegeten. Op hoeveel dagen .. euuuuhm. Nu moet ik wel eens goed denken. Ik twijfel tussen 5 en 6. We zetten 5. Hoeveel heb je de voorbije week gegeten van deze groenten soorten. Rauwkost, ja. 5 keer. Volledig rauwe tomaten. Oei. Neen. Schijfjes tomaat. Nul. Hier drie. Eetlepels geraspte wortelen. Twee. Volledige rauwe wortel. Nul. Eetlepels komkommer. Kan je dat zo eten? Hm. Raar he. Rauw witloof of gekookte mais. Pff. Ahja een eetlepel, nu ben ik mee. Ik ben traag van begrip. Eetlepels van andere rauwe groenten. Gekookte wortels. Bloemkool. Twee. Nul. Andere gekookte groenten, eens denken hé. Neen. Hoeveel groenten denk je dat je eet? Euh .. tussen de twee. Hmm. Fout of helemaal juist. **Wat vind je van die vraag?** Ik denk dat dat juist is als je het zelf al gedaan hebt, als je meer groenten hebt gegeten weet je dat het beter voelt. Maar als je dat nooit eet kan je dat niet weten he. Vind ik hé. Ik ben er zeker van dat ik meer groenten kan eten dan ik nu doe. Ja dat is juist. Meer groenten kan eten .. ja ja. Ik ga dat zetten. Ook als ik denk dat .. ja juist. Ook als ik problemen en zorgen heb. Juist. Ook als mijn familie/vrienden dat niet doen.. juist. **Waarom twijfel je?** Ik had de vraag verkeerd geïnterpreteerd. Ik heb een duidelijk plan voor wanneer ik meer groenten ga eten. Hmm .. neen. Mee oneens. Als er iets tussen mijn plan komt, situaties waar het moeilijk is.. |
|  | 10-11:10 | Oneens. Ja. Hm. Gohja, doelen stellen, dat doe ik eigenlijk niet. Maar ik heb er geen moeite mee. Ik heb er moeite mee om plannen te maken die mij helpen in mijn doelen .. heb ik ook niet. |
| DEEL 1 ADVIES | 11:10 | Je gaf aan dat je op 5 dagen in de week groenten eet. Het is goed dat je groenten eet, positieve stap .. het is aangeraden elke dag 300g groenten te eten. Oei, dat is veel he! Dat is meer dan het dubbele van wat ik eet maar dan nog eens elke dag. Oké. Vandaag kan je nog een stap dichterbij komen door te plannen om je hierbij te helpen heeft deze website enkele tips waarmee je een eigen actieplan kan opstellen. Een voorbeeld van wat anderen hebben gekozen .. ja. Dat zijn voorbeelden? **Nu gaan wij een maken die persoonlijk is voor jou? Je mag op ‘ja’ klikken**. (lacht). Oke ! |
| DEEL 1 OPSTELLEN ACTIEPLAN | 12:36 | *(leest inleiding voor)* **wat vind je daarvan?** Goed, ik vind het wel goed dat dat daar ook instaat en er niet staat van ‘je moet’. Er staat wel ‘300g’ maar er staat niet dat je dat MOET doen om dat te bereiken, dat vind ik goed dat je zelf kan inplannen en zeggen ik voel me goed bij maar 3 of 4 keer per week groenten te eten. Ik wil dan wel veel eten.. denk ik dat ik het in bepaalde situaties moeilijk zal vinden om veel groenten te eten. Neen dat denk ik niet. Hoe ga je proberen om meer groenten te eten dan nu. Door groenten op mijn boterhammen te eten, ja dat doe ik te weinig eigenlijk. Groenten als tussendoortje, hmm.. andere groenten te eten ja**.** Mag je er meerdere aanklikken? **Jaja.** Dat vind ik hier al wel interessant, andere soorten groenten vind ik ook interessant, dat het niet saai wordt. Groenten als aperitief, hmm neen. Salades te eten, ja. Neen. Om mee te nemen naar het werk. Ja. Dat gaan we hier invullen he. Ik zou hetzelfde willen doen als 5. En beter een doelstelling he, 6. Hoeveel groenten wil je eten, dat is een moeilijke he.. ik zie dat niet voor mij 300g groenten hoeveel dat dan is. Ik vind dat nogal veel eerlijk gezegd! Ik ga 250 nemen. Waar wil je vooral groenten eten, thuis. Op het werk ja. Op welke momenten wil je vooral groenten eten, middagmaal. Avond. Tussendoortje, hmm eerder niet. Ontbijt neen. Om zelf een als-dan plan op te stellen kan je het volgende doen .. als ik een tussendoortje eet in de voormiddag dan eet ik een stuk wortel bijvoorbeeld. Ah ja. Ik mag dat zelf intypen he? **Ja.** Opeten of ertussen leggen dat is hetzelfde he. Dan zal ik er twee soorten groenten bij eten. Volgende. Wanneer wil je beginnen? **Je zou vandaag moeten kiezen anders zitten we vast.** Ahja, vandaag he! |
| DEEL 1 ACTIEPLAN | 18:36 | Mijn actieplan! 6 dagen per week 250g, op mijn boterham, andere soorten, salades op restaurant ja dat heb ik eigenlijk zelden gedaan! Ik eet altijd vis, maar dat is ook niet slecht he. Voldoende groenten mee te nemen naar het werk, dat is waar.. ik neem meestal enkel fruit mee naar men werk. Als-dan: als ik ’s middags boterhammen eet zal ik 2 soorten groenten erbij eten. Dan moet ik ze meedoen. Vanaf vandaag. Goed. Wel leuk hé! Ik vul dat wel graag in! Goed. Ik ga dat moeten afwegen he, anders weet ik niet hoeveel 250g is. Ik vind het goed. BMI: huh ?? **247.000 ? dat zal een misser zijn**. Dat is met de komma zijn zeker dat ik verkeerd heb ingevuld! **Ja dat zou kunnen**. hoeveel moet je BMI zijn onder de 25? **Ja, ik denk vanaf 30 is obesitas. Het hangt ook af van je leeftijd.** Suikerrijke of vetrijke koek, een wortel, ja dat is waar. Duidt de methode aan die je zelf wilt gebruiken, men agenda. Daar schrijf ik ook alle sport in die ik gedaan heb. Kalender neen. Een app! Bestaat er een app? **Nog niet**. Dat zou handig zijn he! Volgende. Wil je jou actieplan versturen. Ja ja ! ik zou beter nee zetten he, ik zou niet weten naar wie ik het zou sturen, joa toch wel. Wat moet ik dan doen hun email adres? Godverdikke.. euuuhm. Dat weet ik natuurlijk niet vanbuiten. Mag ik dat eens zoeken? **Ja doe maar!** Voila ! je actieplan is klaar, tijd voor actie, oei! Een eerste stap naar een gezonder leven. **Nu hebben we de eerste module doorlopen en normaal een week erna krijg je een email dan kan je nog eens doorlopen wat je allemaal hebt gedaan in die week, hoe goed je bezig bent.** En krijg je dat via je email adres? **Ja. Maar omdat ik niet volgende week ga terugkomen gaan we dan nu al doen.** **Je moet het je inbeelden.** |
| DEEL 2 VRAGENLIJST |  |  |
| DEEL 2 AANPASSEN ACTIEPLAN |  |  |
| DEEL 2 REST |  |  |
