# Supplementary material for: Users’ thoughts and opinions about a self-regulation-based eHealth intervention targeting physical activity and the intake of fruit and vegetables: A qualitative study
Source: PLoS One. 2017 Dec 21;12(12):e0190020. doi: 10.1371/journal.pone.0190020 (PMC5739439; doi:10.1371/journal.pone.0190020)
Supplement: S3 File — This file contains the transcribed interviews. (ZIP) [file pone.0190020.s003.zip › general_population/TA1BERB.docx]

**Code filmpjes:**

| Deel interventie | Minuten | Transcript |
| --- | --- | --- |
| DEEL 1  VRAGENLIJST | 0-10 | Eet je voldoende porties fruit per dag? Beweeg je genoeg? Ik ben benieuwd! Misschien kom ik wel meer te weten over mezelf! Dat zou tof zijn. Ik heb dat zelf al geprobeerd een eigen actieplan op te stellen maar het is moeilijk je daaraan te houden. Vind ik supergoed want als je jonger bent heb je wel een moeilijkere levensstijl, op 18 kan je al iets serieuzer doen. *(vult persoonlijke gegevens in)* dat is echt verwarrend dat je moet klikken zo bij die leeftijd! Dat is niet goed. 300g groenten, dat is goed he. 2 porties fruit, daar kom ik nooit aan. Dagelijks 30minuten bewegen, ik doe mijn best. Altijd interessant. Chill, ik zie het zitten! We zullen fruit nemen. Ja, het is daar. Heb je al een andere module.. neen. Vaste lijn, oei dat heb ik niet. Wat is je leeftijd. Geslacht. Hoogste diploma. Bachelor he. Dat is waar. Ligt het aan mijn pc of is die site vrij traag? **Ik denk internet.** Aah ja natuurlijk je hebt daar internet voor nodig. **Ja dat gebeurt wel vaker he bij een site!** Oei. **Dat klopt niet, stop dat maar.** Gewoon opnieuw beginnen dan. Hoeveel weeg je, 68. 174cm, niet 100% zeker. Geen diabetes, geen dieet. Op hoeveel dagen heb je de voorbije week fruit gegeten. Een moeilijke vraag.. 2 dagen. Hoeveel verschillende soorten .. ananas. Een banaan. En een mango. Ja. Hoeveel dagen heb je de voorbije week – eerder weinig fruit. Meer fruit gaan eten: ja en nee, misschien wel misschien niet. Ik wil dat wel, maar ik weet dat het moeilijk is, ik probeer echt naar de winkel te gaan en te denken ik moet nog een banaan meenemen. Ik denk dat het niet makkelijk is, dat dat een vast patroon is van naar de winkel gaan en dat voorbij lopen. Ik heb dat niet standaard liggen en dan ligt dat ook maar op mijn kot te stinken. Als ik meer fruit eet dan: is mijn kans op ziektes kleiner, dat is juist. Zal ik me mentaal beter voelen, waarschijnlijk wel een stuk, maar neen. Misschien dat mijn gemoedsrust beter zou zijn maar niet dat ik me mentaal beter voel. Zullen anderen mij steunen, geen idee! Ik weet dat niet, ik denk niet dat ik naar iemand zal gaan en zal zeggen ‘ik eet meer fruit!’ en dat zij dan gaan zeggen ‘yeeey !!’. ik ben er zeker dat ik meer fruit kan eten dan ik nu doe: dat is zeker waar. Dat was een makkelijke vraag. Meer fruit kan eten ook in moeilijke situaties, awel dat is het he, het is die te weinig tijd, maar dat is niet alleen bij fruit eten, maar in het algemeen. Ook als ik telkens opnieuw moet proberen als het lukt, juist. Als ik problemen en zorgen heb, dat denk ik wel ja. Als mijn familie en vrienden dat niet doen.  Ik heb er moeite mee om voor mijzelf doelen te stellen om meer fruit te eten. Ik stel die doelen eigenlijk niet, ik denk er niet zo bewust bij na .. ik heb er dan waarschijnlijk wel moeite mee om ze te stellen. Om plannen te maken die mij helpen mijn doelen te bereiken, ja ik heb daar moeite mee. Neen waarschijnlijk niet, nee doe ik niet. Ik zou al niet weten hoeveel ik er heb gegeten dus dat wil al zeggen.. ik heb een plan voor wanneer ik meer fruit ga eten.. zo rap mogelijk hé. Helemaal mee eens. Ik heb een duidelijk plan voor hoeveel fruit ik ga eten: zoveel mogelijk. Ik heb een duidelijk plan voor wat ik ga doen als er iets tussenkomt. Euuh. Neen dat heb ik niet. Situaties waarin het moeilijk is: JA. Eerst naar de fruitafdeling gaan in de winkel. |
| DEEL 1 ADVIES | (2^e^ fragment) 5:16 | Ze spreken je zo persoonlijk aan, dat vind ik wel leuk. *(leest advies voor)* ik wil zeer graag mijn eigen actieplan, absoluut! Voila. Ik wil eerst nog wat meer informatie lezen over fruit. |
| DEEL 1 OPSTELLEN ACTIEPLAN |  | Ik denk dat ik het vaak moeilijk zal vinden. Ik vind veel fruit lekker, ik eet heel graag fruit. Ik ken niet veel verschillende fruitsoorten: jawel. Ik vind fruit duur? Ja. Ik vind fruit niet lang genoeg houdbaar, ja dat is zeker! Absoluut. Het zou langer houdbaar moeten zijn, dat zou nogal eens een gat in de markt zijn. ik bereid fruit niet graag ja zeker !! dan moet je dat schillen en dat sap en da maakt dan plekken op u broek .. ik heb te weinig tijd, gohja.. een beetje hé. Ik weet niet wanneer ik allemaal – niet supermoeilijk he. Te weinig beschikbaar op mijn werk – neen. Thuis: neen. Ik vergeet vaak fruit te eten: dat is zeker, grootste probleem. De belangrijkste hindernis: ik vergeet het vaak. Door een fruitschaal op een goed zichtbare plaats. Ik zou het toch niet opeten. Ik leef vrij onregelmatig dus dat is geen goed idee. Combineren met andere maaltijden: dat helpt wel! Eerst het bordje leegeten en dan een stukje fruit. Door meer dagen te eten, grotere porties zou ik niet kiezen, want als ik eet is het wel mango. Euuh. Hoeveel dagen: 5 van de 7 zal wel volstaan zeker? Ik moet realistisch blijven ook he. Waar. In de badkamer, de living *(lacht)* thuis, op het werk. Ook bij familie en vrienden. Wanneer: tijdens het ontbijt, middagmaal, dat is het. Als-dan. Als ik ’s morgens ontbijt, dan eet ik een stuk fruit. Vanaf wanneer? **Je moet vandaag ingeven anders werkt het niet.** Mijn actieplan, het is klaar seg! |
| DEEL 1 ACTIEPLAN | 11:23 | Dat is top hé. mijn doel bijhouden in mijn agenda. Via een app, nog beter! Versturen yeey! Op hoeveel dagen? 2 dagen. Neen. Dat vind ik moeilijk, misschien 3 dagen. Dat ik dan 2 bananen heb gegeten en een mango, dat eet ik echt supergraag. Sowieso mango. Alhoewel neen, ik ga het anders doen: zo een dessertschaaltje, ge kunt dat in de albert hein echt voor géén geld krijgen, k ga zo eentje kopen, dan moet ik het niet schillen. Zo ben ik dan weer wel. Nectarines, dat is echt nen brol hé. heb ik dat nu juist gedaan? Ah nee nee. Wat was volgens jou de reden dat je je doel nog niet kon – euhm .. qua motivatie denk ik, dat ik dat soms een beetje mis. Welke informatie zou je graag nalezen .. euuhm.. ik denk tips om meer fruit te eten. Tussendoor eens een stuk fruit eten en tussendoor eens andere soorten fruit, dat is wel echt een goeie tip, dat ik niet altijd hetzelfde neem. |
| DEEL 1 REST |  |  |
| DEEL 2 VRAGENLIJST |  |  |
| DEEL 2 AANPASSEN ACTIEPLAN |  |  |
| DEEL 2 REST |  |  |
