# Supplementary material for: Users’ thoughts and opinions about a self-regulation-based eHealth intervention targeting physical activity and the intake of fruit and vegetables: A qualitative study
Source: PLoS One. 2017 Dec 21;12(12):e0190020. doi: 10.1371/journal.pone.0190020 (PMC5739439; doi:10.1371/journal.pone.0190020)
Supplement: S3 File — This file contains the transcribed interviews. (ZIP) [file pone.0190020.s003.zip › general_population/TA1BERG.docx]

**Code filmpjes:**

| Deel interventie | Minuten | Transcript |
| --- | --- | --- |
| DEEL 1  VRAGENLIJST | 0-10 | *(vult persoonlijke gegevens in)* Het is leuk om te lezen, de kans op ziekten doet het verminderen. Diabetes neen, op hoeveel dagen heb je groenten gegeten de voorbije week? Hmm .. Portie sla, ik heb gisteren bij men boterham een beetje sla gegeten en in het begin van de week ook, veel van die pakjes van die gekuiste sla, volledig rauwe tomaten misschien alles bij elkaar deze week toevallig 1 gegeten. Schijfjes tomaat.. geraspte wortelen niet. **Als het nul is moet je ook nul invullen anders gaat hij straks een foutmelding geven.** Is het zo het terugkeren of zeg nu maar de afgelopen week? **Neen het gaat echt over de voorbije week**. Dan heb ik ook een eetlepel komkommer binnen. Rauw witloof toevallig gisteren, met mayonaise dat eet ik graag. **Dat is wel al minder gezond he mayonaise.** Ja maar dat staat hier niet in de lijst he! Bloemkool of broccoli. Hebben we dat gegeten deze week, neje… ja. Eetlepel dat gaan er snel vier zijn he zo een bloemkes. Rode kool neen, jammer he.. **waarom zeg je jammer?** Ik eet graag boontjes met ajuin. Tussen de twee zo denk ik. Ik heb wel de goesting om meer fruit en groenten te eten maar het vergt een inspanning he, je moet naar de winkel gaan, die groenten kuisen, als ik de dames op het werk ’s middags een groentenslaatje zie maken dan ben ik daar wel jaloers op. Als blijkt uit die enquête dat dat zo is, dan ben ik wel iemand die daar werk gaat van maken. Gemakkelijk is het niet, maar nu ook niet om te zeggen dat dat supermoeilijk is. Als ik meer groenten eet dan … ja. Dat is juist he. We hebben het daarjuist gelezen. Zal ik mij mentaal beter voelen, joa, in kader van mijn sportieve inspanning en dat ik wat minder kilo’s bij krijg. Zijn er anderen die mij steunen.. neen. Ik denk het niet hé. Zouden anderen mij steunen? **Wat denk je bij die vraag**. Als ik meer groenten eet zullen anderen mij steunen, in wat dan? In mijn eetgedrag? **Ja en in je persoon zijn.** Ahja? Neen dat denk ik niet. Ik ben er zeker van dat ik – juist. Moeilijke situaties dat is gewoon dat ik geen tijd heb, maar dan kan ik ook niet meer eten. Ik denk niet dat het voor mij eerder een opgave is van ‘het gaat mij nie lukken’, ik moet kik niet proberen. Ook als ik problemen heb jah – juist. Ook niet echt euh .. als mijn vrienden en familie het niet doen wil dat zeggen dat er geen groenten zijn. **Ja of dat jij ze zelf klaarmaakt en de rest niet.** Juist. |
|  | 10-12:48 | Ik ga niet zeggen helemaal juist, omdat ik misschien zelf niet altijd de moeite ga doen. Ja meer groenten - Ik ben daar mee bezig dat dat gezond is. Ik heb een duidelijk plan – neen he. **Wat vond je van dat onderdeel?** Goh.. vergaand… een plan maken om groenten te eten – neeje. Veel goesting, maar om nu te zeggen ik ga er een plan voor maken, in dat stadium ben ik niet. Ik heb gewoon geen plan. **Waarom blaas je?** Ik hou mijn voortgang bij.. van geen kanten. **Niet voor jou?** Neen, pas op het is niet dat ik dat niet zo willen hé! Maar het is het niet. |
| DEEL 1 ADVIES | 12:48-14:53 | **Wat denk je daarbij?** Dat ik er nog ver af ben he. Ik ben aan het denken in andere weken kan het misschien 150 zijn maar het zal niet – gelijk vandaag. 200g groenten .. elke dag 300gram groenten. **De bedoeling is dat we samen eigenlijk zo een plan gaan opstellen. Om daar dichter bij te geraken. 300g is veel he.** Soms en vaak. Gaat hij ook vragen waarom? **Nee straks.** |
| DEEL 1 OPSTELLEN ACTIEPLAN | 14:53 – 24:55 | Moet ik ze er allemaal opzetten? **De belangrijkste zou ik doen.** Ik bereid groenten niet graag, ik heb te weinig tijd. Kan ik zo slepen? **Ja je kan dat andere dan boven zetten.** Niet graag.. gohja, het is in functie van geen tijd en geen prioriteit.. want ik moet kiezen hé. **Maar je kan er twee inzetten he.** Mag je er drie inzetten**? Drie mag ook.** Gebrek aan steun dat is zwak hé. **Wat bedoel je met zwak?** Welja .. **een zwak excuus?** Ja dat. Wanneer ik allemaal groenten kan eten- jah. Dat er te weinig aanwezig is op het werk.. jah wij hebben fruit, iedere dag hebben we een stuk fruit. Moet je daar nu groenten leggen ik weet het niet, je kan het nie combineren. En rauwe groenten, ik ga niet in zo een witte kool een stuk afbijten of .. kan je het daarmee doen? **Ja zeker als dat voor jou het belangrijkste is.** De rest is zo mjoa, maar niet overheersend. Ook eens diepvriesgroenten .. **wat denk je daarbij?** Awel, dat dat door mijn partner afgepraat wordt van dingen uit blik te kopen, omdat dat als een stuk van zen waarden verliest. Maar ja, dat ze natuurlijk een oplossing zijn en als ik zou kiezen tussen diepvries op blikgroenten, dan zou ik blikgroenten nemen. **Maar je mag meerdere antwoorden kiezen ook hé.** Ja, eigenlijk zou dat een oplossing kunnen zijn en dat ook. Voor mij is dat al op voorhand gaan plannen. **En dat wil je niet?** Dat is raar he. Want ik kom er niet toe, en waarom ja, puur ingesteldheid he. Maar als je ’s avonds thuis komt en dan moet je rond half 9 zeggen van ik ga groenten kuisen dat ik mijn potje heb voor de dag nadien en de dag daarna, het zou prachtig zijn, maar die goesting ja, ik kan het mijzelf niet opleggen. **Maar de bedoeling is wel dat je haalbare doelen stelt.** Eén keer op voorhand zou dan toch wel moeten lukken he, het zou toch een engagement moeten zijn he. **Ja, meerdere antwoorden zijn mogelijk.** Ik wil eigenlijk iedere dag groenten eten, maar is dat dan niet wat té? Ik ga de limiet niet zo hoog zeggen, ik zet hem op 6. Anders .. hoeveel groenten wil je eten op die dagen.. nu kom ik aan 100g, dat is te weinig. **Ja de dagelijkse aanbevolen portie is 300.** Dan ga ik daar wat bij doen he. Op het werk, misschien moet ik ook wat kijken op restaurant een menu met groenten. Bij familie en vrienden? Dat is te zeldzaam om te zeggen dat we daar een doel van gaan maken! No way, zeker niet tijdens het ontbijt! Als tussendoortje in de voormiddag ook niet. Pas vanaf de middag. ‘als-dan’ plan. Ik heb gezegd ’s middags? **Tijdens je middagmaal en je avondmaal.** Ja. Ik denk dat ik gewoon ne schep meer ga nemen. **Ja daar kan je ook een plan van maken om een portie meer op te scheppen.** Als ik ’s middags mijn boterhammen eet, dan zal ik zorgen dat er een stuk rauwe groente bij zit. Als ik groenten eet, dan zou ik moeten zeggen nog een extra portie. **Je kan er maar 1 invullen.** Nu is dat niet altijd zo, het is maar 20 % het geval dat er een stuk rauwe groente bij zit. **Dan kan je inderdaad zeggen ‘zorgen dat er meer groente bij ligt’.** **Nu ga je een kalendertje hebben, maar je moet eigenlijk vandaag aanduiden anders klopt mijn programma niet meer.** Ahja. Mijn actieplan. |
| DEEL 1 ACTIEPLAN | 24:55 – 31:49 | **Wat vind je daarvan? Van dat stuk?** Dat die 300g dat dat veel gaat zijn, maar ik ga me wel voornemen meer groenten te eten en eens een stukje vlees minder. **Als je wilt kan je ook teruggaan op je actieplan te wijzigen**. Als je zegt van 300g is veel? Neen dat moet lukken. Want het is een primeur maar vanmiddag ga ik bevestiging krijgen of ik mee doe aan de Amstel of niet, en dan ga ik beginnen fietsen en gezonder eten. Ik zou 3 kilo moeten afvallen, eigenlijk 5 om goed te zijn. Dat is véél he dat kan bijdragen. Maarja dat is ook maar tijdelijk dus dat mag het niet zijn he. Neen maar ik vind dat dat moet lukken. **Het programma loopt eigenlijk een maand zogezegd.** Zo 1 witloof hoeveel zou dat wegen? **Een volledige broccoli, zo de roosjes, dat is 300g. of 3 wortels, 2 paprikas en een bakje kersttomaatjes.** Dat is ook 300. Godverdomme seg! **Dat is over de hele dag he, dat moet je niet enkel s avonds eten.** Ja maar dat is ook al veel he! Wat eet ik, 2 witloven. **Dat zal toch al wat zijn ze**. En een paar patatten. **Maar patatten**- tellen niet echt mee he ja. Maar zo rauwe groenten, dat weegt niets he. **Dat is heel weinig. Maar een slaatje bestaat voor een heel groot deel uit salade en dat is toch al iets he.** Dat is veel he.. maar toch zou het moeten. Het zal wel met de komma inbegrepen zijn. Oei!!! BMI te bespreken met jouw huisarts. **Ja omdat je BMI iets aan de hoge kant ligt. Je hebt geen overgewicht he, maar het is ietsje aan de hoge kant**. Wat zou het moeten zijn? **Normaal mag het wel tussen de 20 en 24 liggen. Dat hangt ook af van je leeftijd.** Dus het is puur gewicht gelinkt aan gestalte en leeftijd? **Ja.** Ik moet eigenlijk 80 kunnen wegen… dat is wel iets dat een belangrijke rol zijn my wife, ook in dat niet akkoord zijn met die blikgroenten of diepvriesgroenten. Mag je meerdere mensen kiezen? **Ja.** **Wat vind je van dat onderdeel?** Moeilijk. Dat zal stuiten op commentaar. **Maar je doet het toch?** Ja omdat ik haar die blikgroenten wil laten zien! Dat dat belangrijk is dat ik aan mijn totaal kom en weinig tijd heb en steun vraag. De partner gaat klagen ‘je moet het maar opeten en ik kook gezond’. Njah .. naar wie zou ik het anders moeten sturen. Mijn secretaresse op het werk zou zeggen ‘wat is dat met den dienen’. |
| DEEL 2 VRAGENLIJST | 2^e^ fragment | Huisarts, groenten .. **dat is de naam van het programma. Dus nu zitten we een week verder he.** Ik ga eerlijk zijn – op 5 dagen. We hebben redelijk wat sla gegeten. **Het staat erbij he, portie rauwkost is ongeveer 50g.** dat gaan we toch wel een keer of drie in de week gedaan hebben. Ik heb geen rauwe tomaten gegeten maar wel schijfjes. Toch wel een schijfje of 15. Geraspte wortelen niet omdat we te veel moeten opkuisen en afdrogen, dat kost ons te veel tijd. Ik heb bewust 3 rauwe wortels gegeten en komkommer 6 eetlepels. Eetlepels witloof, toch wel een 10-tal. Eetlepels van andere groenten, nul. Gekookte wortelen, bloemkool, toch wel 10 in totaal. Witte kool rode kool en daar hebben we ons blikje bonen gebruikt en dat waren 4 eetlepels in totaal, ik heb dat gemeten. Gekookt witloof ja wij hebben 1x witloof gegeten en prei dat is juist. Dus laat ons zeggen dat dat in het totaal 10 eetlepels waren. Andere groenten, ajuin. Dus dat was bij elkaar toch wel 2 eetlepels. We moeten eigenlijk naar 7x 350g . **maar je doel was 6 dagen op een week he? En je hebt er 5!** Ja maar ik heb wel gecompenseerd he. Ik ben gedeeltelijk geslaagd. Door omstandigheden, geen tijd is dat of onvoldoende gemotiveerd? Want je hebt altijd tijd voor de dingen die je belangrijk vindt he, dat zeg ik ook altijd tegen de collega’s: geen tijd is niet waar, geen prioriteit**. En wat is de omstandigheid dat je die ene dag geen groenten hebt gegeten**. Twee dagen niet. Omdat er niets voorraden was. **Dan mag je dat bij ‘andere’ schrijven.** |
| DEEL 2 AANPASSEN ACTIEPLAN | 6:02 | ‘ik wil graag nieuwe en haalbaardere doelstellingen’ ja ik wil toch nog proberen, dat ik aan 385 geraak. **Je mag nog altijd zeggen wat er bij je opkomt het bij het doorlopen van het programma.** Het klonk praktisch onhaalbaar wanneer ik heb vorige week ingevuld heb, en als ik dan zie wat ik gegeten heb, dat ik daar niet meer van mijn doelstelling ben, dan zeg ik van oke ja dat is een haalbaar doel, en ja, dankzij die doelstelling kom ik dan in de buurt, dat ik er bewust een project van gemaakt heb. Ik ga ook groenten kweken in de tuin. **Dat is leuk.** |
| DEEL 2 REST |  |  |
