# Supplementary material for: Users’ thoughts and opinions about a self-regulation-based eHealth intervention targeting physical activity and the intake of fruit and vegetables: A qualitative study
Source: PLoS One. 2017 Dec 21;12(12):e0190020. doi: 10.1371/journal.pone.0190020 (PMC5739439; doi:10.1371/journal.pone.0190020)
Supplement: S3 File — This file contains the transcribed interviews. (ZIP) [file pone.0190020.s003.zip › general_population/TA1BERI.docx]

**Code filmpjes:**

| Deel interventie | Minuten | Transcript |
| --- | --- | --- |
| DEEL 1  VRAGENLIJST | 0-8 | *(vult persoonlijke gegevens in)* Ik moet hier nu zeggen waarop ik wil focussen? **Inderdaad.** Ik denk dat ik tamelijk veel beweeg en wel veel groenten eet, ik ga focussen op fruit. Heb je al een eerdere module ingevuld neen.. email adres.. leeftijd 56 .. man .. universitair onderwijs .. hoeveel weeg ik jah, 64. Lengte 172 tenzij ik zou gekrompen zijn dat kan, geen dieet en geen suikerziekte. Op hoeveel dagen heb je de voorbije week fruit gegeten, hmm hmm .. ik ga gokken hoor, ik denk toch wel 5 van de 7 dagen. Van welke soort .. schaaltjes fruitsla nul, appels peren bananen, zeker 5. Ik ga bij mandarijnen sinaasappelen nemen he? **Dat staat apart**. Ahja oke. Ananas ook niet. Dessert schaaltjes, neen. Stekelbessen. Schaaltjes aardbeien, neen. We zijn gisteren op restaurant geweest en we hebben daar een aardbeiendessert gegeten, is dat een aardbeienschaaltje? **Ja dat mag je zo noemen.** Handen vol kersen of krieken neen, maar daar is het niet het seizoen voor he. Nectarines ook niet. Mango’s vind ik wel lekker! Maar niet gegeten. Meloen is ook buiten het seizoen. Lychees .. lang geleden dat ik dat gegeten heb. Trosjes druiven eet ik regelmatig maar niet vorige week. Eetlepels andere soorten, even nadenken. Neen. Ik denk het niet. Schijven fruit uit blik neen. Fruitmoes niet neen. Het zou kunnen dat ik niet aan mijn standaardportie kom. Hoeveel fruit denk je dat je eet .. eerder weinig fruit. Waarschijnlijk wel. Ik eet gemakkelijker groenten dan fruit omdat ik vind dat fruit wat meer werk met zich meebrengt, schillen enzovoort. Als ik meer fruit eet is het kans op ontwikkelen van ziektes kleiner, dat is helemaal juist. Zal ik me mentaal beter voelen, dat zal juist zijn. en zullen anderen mij steunen, ja mijn vrouw zou mij zeker steunen. Die vindt dat ik te weinig fruit eet. Volgende. Ik ben er zeker van dat ik meer fruit kan eten, ja dat is juist. Ook in moeilijke situaties ja. ook als ik telkens opnieuw moet proberen tot het me lukt: dat is wel een rare vraag… maar dat zal wel lukken he. Ook als ik problemen en zorgen heb.. jah. En ik ben er zeker van dat ik meer fruit kan eten, ja zeker en vast. Ik heb er moeite mee om voor mezelf doelen te stellen neen zeker niet. Ik heb er moeite mee om plannen te maken om mijn doelen te bereiken. Waarschijnlijk niet. Ik hou mijn voortgang bij, jah waarschijnlijk niet. Dat klinkt nogal wetenschappelijk he.. ik heb een duidelijk plan voor wanneer ik meer fruit ga eten. Neen dat heb ik nog niet. Dat kan nog komen he. Ik heb een duidelijk plan voor hoeveel fruit ik ga eten, hoeveel .. euhm.. ja neen geen plan wat dat betreft. Ik heb een plan voor als er iets tussen mijn plan komt te staan: dat is eigenlijk wel een goede vraag. Mee eens. Maar ik heb het nog niet. Neen. Dus ik moet eigenlijk oneens antwoorden, maar ik zou het wel willen hebben. Ik heb een plan voor in situaties waarin het moeilijk is om meer fruit te eten, hetzelfde hier eigenlijk. Ja. |
| DEEL 1 ADVIES | 8:00-8:55 | Ik eet 5 dagen per week fruit, het is goed dat je fruit eet … ja. dus in plaats van 5 naar 6 of 7 dagen gaan. dus mijn plan zou dan zijn om eigenlijk 6 of 7 dagen in de week fruit te eten. Ja. |
| DEEL 1 OPSTELLEN ACTIEPLAN | 8:55 – 14:26 | Hoe wanneer en met wie.. ik denk dat er soms wel moeilijke situaties zullen zijn, en dan is dat meestal tijdsgebrek he. Welke opties kunnen hindernissen vormen om meer fruit te eten.. ik lust wel fruit, ik ken ook wel veel soorten, duur neen, geen gebrek aan steun integendeel, dat fruit niet lang genoeg houdbaar is dat vind ik niet, ik bereid het niet graag, ja dat klopt. En ik heb niet altijd veel tijd. Ik vind dat er te weinig – er is nu op mijn werk toch ook wel aandacht besteed aan gezond eten en meer bewegen, en er is meer fruit beschikbaar in het bedrijfsrestaurant. Maar het probleem is dan dat ik moet kiezen tussen een portie fruit en een portie chocolademousse en ja, dan is het meestal chocolademousse. Maar chocolade is ook heel gezond blijkbaar! Ik denk er niet altijd aan, ik vergeet het. En ik heb vaak zin om iets anders te eten dan fruit zoals chocolademousse. Wat is de belangrijkste hindernis: ik bereid het niet graag. Dus als iemand tegen mij zou zeggen: ‘hier een portie fruit’ zou ik het veel meer eten. Voorgesneden diepvriesfruit, daar heb ik nog niet aan gedacht. Blikfruit.. dat is niet zo lekker. Drie is wel interessant. K ga dat nemen. Andere .. iemand vragen om voor mij te bereiden. Mijn vrouw dus hé. Dus ik kies voor de meer dagen, geen grotere porties. Ja, toch allebei. Meer dagen én meer fruit. Hoeveel porties fruit wil je eten op de dagen dat je fruit eet? 1. Ik ga niet overdrijven. Hoeveel dagen per week, laten we zeggen 6. Waar wil je vooral : thuis of op het werk. Wanneer .. tijdens het ontbijt niet. Ik vermoed dat confituur niet telt als fruit. Want ik eet altijd boterhammen met aardbeienconfituur. Maar ontbijt neen, dat smaakt me zo niet dan. Een tussendoortje in de voormiddag dat is wel een goed idee. Middagmaal niet, en tussendoor dan weer wel. ’s Avonds eignelijk. Ja tussendoor hé eigenlijk, ’s morgens ’s middags en ’s avonds. Een als-dan plan: als ik een tussendoortje eet, zal ik eerder een stuk fruit eten dan iets zoets. Ik eet nogal veel tussendoortjes. Ik ga het vooral houden bij de tussendoortjes, ik denk dat ik daarvan het meest effect zal hebben. Vanaf wanneer wil je meer fruit eten. **Je moet vandaag ingeven anders gaat het systeem blokkeren.** Dus vandaag zijn we de 9^e^. Vanaf vandaag dus! Een meerkeuzevraag zonder meer keuze. |
| DEEL 1 ACTIEPLAN | 14:26 - | Mijn actieplan, goed. Meer fruit. Zonder dat ik het hoef te bereiden. Wat vaker eens een kiwi kopen. En bananen inderdaad. En tussendoortjes inderdaad; vanaf nu. Ik vind dat een goed actieplan. Ik kan dus bijhouden hoe ik mijn actieplan opvolg, ik kan dat noteren. Duid de methode aan. In mijn agenda kalender neen gsm ook niet , app .. ik ga dat niet bijhouden. Wil je je actieplan versturen? Volgende. Amai tot vijf personen, dat is voor enthousiaste deelnemers! Het actieplan is gemaakt… **gewoon op versturen klikken**. Oke versturen. |
| DEEL 2 VRAGENLIJST | (2^e^ fragment) | Wat moet ik nu eigenlijk doen, nog eens hetzelfde? **Nu doe je alsof dat we een week verder zijn**. aah oke goed. Ik vul dan 7 dagen in he, en wat ga ik meer gegeten hebben, ik ga ofwel een banaan… of geen dessertschaaltjes nemen. Geen vijgen. Mandarijntjes wel. Verse pruimen. Handen vol kersen of krieken, nu nog niet. Nectarines daar ben ik niet zot van. Mango vind ik wel lekker. Meloen, lychees niet. Trosjes druiven. Dat is dan nul. Niet uit blik. Geen fruitmoes. Zo. Ik heb iets niet beantwoord zie ik. **Misschien de eerste vanboven jah.** Ja goed. Ik moet me dan schuldig voelen als ik dat niet gedaan heb hé. Dat is goed he, een goed begin.  Je bent erin geslaagd om meer dagen fruit te eten, niet om meer porties fruit te eten. Ja .. motivatie. Hmm hmm. Ik heb genoeg informatie. Ik kan wel eens opzoeken hoeveel fruit ik – fruit kan groenten niet vervangen neen denk ik. Veel toegevoegde suikers he in confituur en siroop enzo. 2 stukken fruit per dag, interessant! Tips om fruit te eten. Fruit op een boterham, dat heb ik vroeger nog gedaan als ik jong was, dan at ik schijfjes banaan op mijn boterham. Dat is ergens verdwenen. Fruitdessert.. ja dat eet ik wel! Yoghurt met vers fruit. Ik eet soms ook muesli met vers fruit, ook met noten. Verse ananas.. hmm hmm. Stekelbessen vind ik te zuur. Verse pruimen. Kiwi’s. ook bij de warme maaltijd, die gewoonte hebben we ook niet behalve soms appelmoes. Bij salades kan je fruit toevoegen, daar denk ik ook te weinig aan. Daar ben ik ook zo wat twijfelachtig bij. En fruit meenemen als ik ga werken. Wat wil ik nog verder doen. Blijven proberen! Hetzelfde doel nastreven. Vanaf nu he. Ziezo! Bij hoeveel mensen moet je dat doen? **In totaal met 20 mensen.** |
| DEEL 2 AANPASSEN ACTIEPLAN | (3^e^ fragment) | Heb je de vorige keer een actieplan opgemaakt om meer fruit te eten, ja. oke. Wat ga je doen: meer fruit 6 dagen per week, eens doen alsof ik een heel goeie leerling ben he! Ik ga op mijn werk eens een schaaltje fruitsla eten volgende week. En banaan appels en peren. Daar gaat er niet van komen denk ik, ik moet realistisch zijn. en verse aardbeien ook eens op een schaaltje. Verse abrikozen dat denk ik niet. Mandarijntjes ga ik blijven eten. Kersen of krieken. En kiwi’s ga ik regelmatig nemen, veel vitamine C. Fruitmoes ook niet. Nog altijd gedeeltelijk geslaagd. Tijd en motivatie. Eigenlijk als je gemotiveerd bent heb je altijd tijd! Dit is het einde? Versturen. |
| DEEL 2 REST |  |  |
