# Supplementary material for: Users’ thoughts and opinions about a self-regulation-based eHealth intervention targeting physical activity and the intake of fruit and vegetables: A qualitative study
Source: PLoS One. 2017 Dec 21;12(12):e0190020. doi: 10.1371/journal.pone.0190020 (PMC5739439; doi:10.1371/journal.pone.0190020)
Supplement: S3 File — This file contains the transcribed interviews. (ZIP) [file pone.0190020.s003.zip › general_population/TA1BERN.docx]

**Code filmpjes:**

| Deel interventie | Minuten | Transcript |
| --- | --- | --- |
| DEEL 1  VRAGENLIJST | 0-9:10 | *(vult persoonlijke gegevens in)* **je mag gerust zeggen want je** **denkt.** Geen diabetes, arts heeft me nooit afgeraden om te bewegen wegens hoge bloeddruk .. fysieke activiteiten in de vrije tijd, op het werk. Als je werk hebt he! **Ja dat klopt**. Hm, interessante vraag. 10 minuten fietsen om ergens heen en terug te gaan, weinig.. als je met je fiets verplaatst, middelmatig tempo. Op welke dag wandel je teminste 10 minuten, toch wel 6 op 7. We zitten toch wel aan een uurtje. Ook middelmatig tempo. Klusjes en gezinstaken, euuhm.. in de tuin of moestuin, dat heb ik niet helaas. Dus dan zal het geen zijn eh. Zware activiteit in de tuin, neen. Matige fysieke activiteit binnenshuis, een uurtje. Sport/ontspanning in de vrije tijd. Ja oke. Drie dagen hé. De maandag is dat 4 uur, de dinsdag is dat een 2-tal uren en de woensdag een uurtje. Ik ga hier meer dan 2 uur zetten want dat is zo hé. Zware activiteiten, neen totaal niet. En dan wandelen ja, wacht he.. gewoon wandelen, dat zal 1 dag zijn. en dan kan dat wel 2 uur zijn. tempo, laag tempo. Fysieke activiteit tijdens het werk, ik werk niet momenteel. Hoeveel beweeg je denk je, algemeen veel. Ben je van plan meer te bewegen de komende maand, dat denk ik niet. Ik zit altijd wel rond hetzelfde. Hoe moeilijk of gemakkelijk denk je dat het is om meer te bewegen: dat is altijd gemakkelijk voor mij, er mag iets bijkomen ook, daar heb ik geen probleem mee. Als ik meer beweeg is mijn kans op het ontwikkelenvan ziektes kleiner, ja daar ben ik volledig mee akkoord. Zal ik mij mentaal beter voelen, juist. Zullen anderen mij steunen. Neen daar word ik niet in gesteund, dat is ook niet nodig. Ik ben er zeker van dat ik meer kan bewegen dan ik nu doe, ja je kan altijd meer he. Meer kan bewegen ook als ik problemen en zorgen heb, ja dat is ook just. Ook als mijn familie en vrienden dat niet doen.. dat heeft er niets mee te maken.  Ik heb er moeite mee om voor mezelf doelen te stellen.. neen. Ik heb er moeite mee plannen te maken, neen. Ik hou mijn vooruitgang bij : neen eigenlijk niet, waarschijnlijk niet. Ik heb een duidelijk plan voor wanneer ik meer ga bewegen. Ik heb een duidelijk plan voor hoeveel ik ga bewegen. Ik heb een duidelijk plan voor als er iets tussen mijn plannen komt.. ja dat wel. Ik probeer dat in evenwicht te houden. Stilzitten is niet goed. |
| DEEL 1 ADVIES | 9:10-10:45 | Dat is een beetje een overzicht he. Heel goed. Je haalt de gezondheidsnorm. Blijf dit volhouden. Dat is een beetje een overzicht dan. Ja. deze website geeft je tips waarmee je een actieplan kan opstellen .. Hieronder kan je een voorbeeld zien van Charlotte en Bart. |
| DEEL 1 OPSTELLEN ACTIEPLAN | 10:45 – 15:00 | Denk je dat je het in bepaalde situaties moeilijk zal vinden om meer te bewegen.. neen eigenlijk niet. Wees wat actiever in het huishouden hmm.. ja hier wel in feite ja. ik kan er maar een aanduiden, ik ga die aanduiden dan. Voila. Meer actief te verplaatsen eigenlijk wel. Te voet of met de fiets naar de winkel, familie, vrienden.. vroeger van de bus of tram te stappen.. eigenlijk alletwee, dat is niet slecht. Welke momenten.. meerdere antwoorden ja. winkel zeker, bakker.. toch wel een dag of drie vier. Pak maar vier. Als-dan plan: dat voorbeeld hier is echt een heel goede. Ik ga die overtypen. Je kan niet zeggen in de toekomst of wel? Of is het tegenwoordige tijd? **Je mag zelf kiezen wat je typt daarin!** Aahja oke. **Daar mag je de datum van vandaag invullen**. |
| DEEL 1 ACTIEPLAN | 15:00 – 31:00 | *(leest actieplan voor).* Versturen naar anderen*..* Ik denk dat er weinig mensen zijn die mijn voorbeeld gaan volgen.  **Dat was eigenlijk de eerste module. En dan krijg je een mail van een week later om terug te keren naar de site, maar nu gaan we die onmiddellijk opnieuw doorlopen, je hebt natuurlijk geen week tussen gehad, maar je moet je eigenlijk voorstellen dat het zo is.** |
| DEEL 2 VRAGENLIJST | *(2^e^ fragment)* | Nu moet ik al eens goed nadenken he. Een half uurtje .. welk tempo, dat blijft hetzelfde. 6 op 7. Een uurtje. Welk tempo, middelmatig. In een gewone week in en rond het huis ja, dat is weer met die tuin en moestuin he. Die heb ik niet. Dus dat blijft hetzelfde. Dat is met de boodschappen erbij he, ik reken toch op een half uurtje. Dat zal een lager tempo zijn want je hebt wat gewicht in je handen. Neen, blijft hetzelfde .. ja. meer actief verplaatst. 3 à 4 dagen. Ja.  Dat is nog eens een overzicht dat je fysiek actief bent. Ja. hetzelfde doel. Oké. Hmm .. nog eens het actieplan ja. ja. naleven van het actieplan, nu is het weer versturen hé. **Dat is het laatste dat we vandaag zullen doen. Het enige wat ik zou willen vragen is om een heel kort vragenlijstje over de website in te vullen.** |
