# Supplementary material for: Users’ thoughts and opinions about a self-regulation-based eHealth intervention targeting physical activity and the intake of fruit and vegetables: A qualitative study
Source: PLoS One. 2017 Dec 21;12(12):e0190020. doi: 10.1371/journal.pone.0190020 (PMC5739439; doi:10.1371/journal.pone.0190020)
Supplement: S3 File — This file contains the transcribed interviews. (ZIP) [file pone.0190020.s003.zip › general_population/TA1BERR.docx]

**Code filmpjes:**

| Deel interventie | Minuten | Transcript |
| --- | --- | --- |
| DEEL 1  VRAGENLIJST | 0-10 | De aanspreking vind ik echt niet welkom en er mag geen komma achter hallo. *(leest inleiding verder voor)* Ik vind het vrij kinderachtig geschreven, de benadering vind ik iets te onvolwassen zeker als je ziet dat het vanaf 18 jaar is. Dit vind ik meer voor pubers of jongvolwassenen. Dat tweede stuk vind ik tof, dat is mooi uitgelegd. ‘intense intensiteit’ is heel raar om te lezen. Ik zou ‘bovenstaande’ richtlijnen zeggen, anders.. **het is meer de bedoeling dat je zegt wat bij je opkomt, maar niet op vlak van lay-out**. Ah ja oke. Ik voelde mij zo een beetje beangstigd omdat ik weet dat ik niet aan de voorwaarden voldoe en dat is wel zo van oei! Ik ga voor veiligheidshalve fruit kiezen, omdat ik daar het meest aan voldoe. Beweging zou voor mij de grootste uitdaging zijn, het hangt er van af wat het doel is van de website. **Je mag zelf kiezen wat je wilt verbeteren.** Dan ga ik voor beweging*. (vult persoonlijke gegevens in).* Euhm. Minstens 10 minuten. Ik heb geen fiets. Dat is wel net iets meer. Dat is vrij moeilijk om in te schatten altijd .. euhm .. hoor hier presidium bij of vrijwilligerswerk? Want dat zijn de dingen die je rond het huis doet als dingen heffen? **Ik zou dat als werk beschouwen.** Oké. Dat kan ook als vrijetijd- dat is heel bewust van ik ga sporten veronderstel ik? **Ja.** Dat is zo pijnlijk. Neen .. neen.. oke vrijwilligerswerk, studiewerk.. dat weet ik echt niet. Als je een avond werkt, is dat dan wandelen? |
|  | 10-13:00 | **Het is zoals je het zelf interpreteert.** Dan ja. Als ik over en weer tjol met cocktails. Het is niet dat ik de hele dag in mijn zetel zit ook niet. Kunnen en willen is twee he. Ik vind dat dat extra motiveert. Dat wel, ik heb een duidelijk plan. Ja een plan om het gewoon af te zeggen is ook een plan. |
| DEEL 1 ADVIES | 13:29 | Het spreekt mij aan dat het op maat is gemaakt, ik vind het ook geruststellend van als ze dat zeggen dan moet je dat kunnen. Langs de nadere kant voel ik mij wel goedgelovig omdat het een online test is. Oke maar dat doe ik op zich, het is meer bewegen als hobby dat lastig is, met de trap gaan te voet of de fiets dat is geen probleem, dat past in mijn tijd. |
| DEEL 1 OPSTELLEN ACTIEPLAN |  | Het is moeilijk want alles hangt zo af van dag tot dag en week tot week, want niet elke dag of week is hetzelfde en op maandagmorgen lukt het wel om ’s morgens te gaan lopen, dat je een beetje afweegt maar de dag erna kan je dan niet uit je bed enzo .. ik zou sowieso ’s ochtends of ’s avonds lopen maar niet ’s middags. Als het weekend is, dat is geen voorwaarde om te lopen. Ik denk dat het makkelijker is dan in een weekend. Dat vind ik lastig, ik wil wel sporten en dat op zich is een half uurtje dat je bezig bent, maar je moet je omkleden, douche, dus je bent zoveel meer tijd kwijt dan dat je bezig bent. **Ja maar nu gaat het over de sport op zich.** dat vind ik even lastig omdat dat niet elke week is, of niet elke maandag. Dan is het wel de moeite want dan verlies je minder tijd. Ik ga muurklimmen, daar moet ik wel een partner voor vinden, dan moet je iemand vinden die op dat moment ook kan én die dat wilt doen.. moet ik nog verder. **Dat is niet verplicht**. Is dat normaal dat het zo traag gaat? **Dat ligt aan het internet denk ik.** **Je zou vandaag moeten invullen anders blokkeert het systeem.** Haha oke! Eigenlijk maak je het zelf- oké. |
| DEEL 1 ACTIEPLAN |  | Dat is echt veranderlijk van week tot week of welke dagen .. ik vind dat een heel raar gevoel omdat je het voor jezelf moet doen en de controle van anderen daar eigenlijk niet voor nodig hebt. Ik zou dat doen via een app dat je kan registeren hoeveel je hebt bewogen hoeveel oefeningen je bijvoorbeeld gedaan hebt. Oké. Ik vind het heel fijn dat je nu beseft van, je krijgt zogezegd je persoonlijk actieplan maar je hebt het wel zelf gemaakt waardoor het voor jou wel werkt, en het gepersonaliseerd is. |
| DEEL 1 REST |  |  |
| DEEL 2 VRAGENLIJST |  |  |
| DEEL 2 AANPASSEN ACTIEPLAN |  |  |
| DEEL 2 REST |  |  |
