# Supplementary material for: Users’ thoughts and opinions about a self-regulation-based eHealth intervention targeting physical activity and the intake of fruit and vegetables: A qualitative study
Source: PLoS One. 2017 Dec 21;12(12):e0190020. doi: 10.1371/journal.pone.0190020 (PMC5739439; doi:10.1371/journal.pone.0190020)
Supplement: S3 File — This file contains the transcribed interviews. (ZIP) [file pone.0190020.s003.zip › general_population/TA1BERT.docx]

**Code filmpjes:**

| Deel interventie | Minuten | Transcript |
| --- | --- | --- |
| DEEL 1  VRAGENLIJST | 0-10 | Ik moet nog wat verbeteren – als ik een maniak wil zijn moet ik nog verbeteren, maar dat wil ik niet zijn dusja. *(vult persoonlijke gegevens in).* De 300g groenten eten per dag, ik weet dat dat de norm is en 2 porties fruit ook, ik moet daar een gedacht van maken van die 2 stukken fruit en 300g groenten probeer ik ook aan te geraken maar euh het is 1 ding dat dat een beetje werk vraagt om verse groenten alle dagen op tafel te zetten, dat is het nadeel. En ja bewegen, dat moet ik me soms wel eens forceren om te gaan lopen. Vroeger was ik er 100% van overtuigd dat ik veel bewoog omdat ik lopend werk had, maar nu is het zittend werk dus nu denk ik toch dat ik in mijn vrije tijd meer moet bewegen. Ik zou inderdaad wel een beetje meer moeten bewegen. Want de voorraden van de menopauze – **je mag kiezen welk van de drie je gaat op verdergaan**. Bewegen. *(vult gegevens in)* hoeveel dagen in een gewone week.. eigenlijk in de zomer fiets ik regelmatig en in de winter niet he. **Je moet rekening houden met dit seizoen.** **Een gewone werkweek.** Dan geraak ik misschien aan 3 dagen. Ik fiets niet zo lang. Eerder een laag tempo. Ahja hoe je het zelf aanvoelt, dan vind ik dat het een middelmatig tempo is. Wandelen.. Op een gewone dag eigenlijk niet. **Je mag nog altijd positieve en negatieve dingen zeggen.** Wat bedoel je? Bedenkingen bij de site? **Niet bij de site maar bij de vragen, je mening.** **Soms helpt het als je luidop leest.** Licht werk in de tuin of moestuin, praktisch niet. |
|  | 10-20 | Eerlijk gezegd geen. Zware fysieke activiteit in de tuin, ook geen he. Matig fysiek binnenshuis: zeker 1 dag he. We gaan er 2 dagen van maken. Als je eens een dag van plan bent om te kuisen zit je toch rapper aan meer dan 2 uren he, maar je kan niet meer invullen dan 2 uur. Recreatiesport: iets sneller en dieper ademt dan normaal: dat doe ik altijd, als ik loop denken ze altijd dat ik niet gewend ben van te lopen omdat ik zo een zware ademhaling heb. 1 dag. Hoeveel tijd aan matig FA: 1 uur. Zware FA: dat zijn dan de activiteiten dat je zelf zwaar vindt? Als ik een uur lopen zwaar vind, dan zit dat bij die zware? **Ja matig is meer als je vindt van ik heb een halfuur gewandeld en het was vrij zwaar..** dus dan heb ik hier verkeerd geantwoord? **Ja waarschijnlijk wel. Er staan een paar voorbeelden bij he.** **Ook naargelang hoe zwaar je het zelf vindt.** Als ik zelf ga lopen vind ik het wel zwaar, ook al is het niet in voorbereiden van een wedstrijd. **Dan mag het bij zwaar.** Wanneer wandel je 10 minuten in je vrije tijd.. zeker niet wekelijks. Je hebt hier wel geen keuze he. Moest je zeggen in de maand kan ik zeggen 1 keer, maar dat kan je niet .. dus moet je ofwel geen pakken ofwel 1 dag en daar geraak ik niet alle weken aan. Dus dan pak ik best geen he? **Ja.** Activiteit op je werk: eigenlijk heb je maar keuze tussen matig en zwaar, maar ook al doe ik fysiek redelijk zware activiteiten, wat echt sport is ga ik niet sneller en dieper ademen. Huishoudelijke taken inet he? 2 dagen. Meer dan 2 uur. Zware fysieke activiteiten: jah. Dat is beetje ertussen he. 2 dagen, kun je niet echt zwaar noemen dus ik ga 1 nemen. Wandelen als deel van je werk, die twee dagen he. Als we de kilometerteller pakken zitten we gemakkelijk aan 9(?) kilometer. Hoeveel beweeg je denk je. Niet veel niet weinig. Ben je van plan meer te bewegen, waarschijnlijk wel. Hoe moeilijk of makkelijk denk je dat het is. Tgoh. **Je mag nog altijd positieve en negatieve dingen zeggen.** Als ik meer beweeg is mijn kans op het ontwikkelen van ziektes .. helemaal juist. Zal ik mij mentaal beter voelen ook helemaal juist. Zullen anderen mijn steunen ja, ze zullen dat toejuichen als ik meer sport. Ik ben er van overtuigd dat ik meer kan bewegen dan dat ik nu doe, juist. Ook in moeilijke situaties: ja. Ook als ik telkens opnieuw moet proberen, goh, wat bedoel je daarmee? **Als het niet lukt, dat als je blijft proberen dat het wel gaat lukken?** Bijvoorbeeld in de zomer ga ik dan meer tussenin oefenen, ja. **Het gaat eigenlijk meer om de intentie**. Ahja. Dat is waar. |
|  | 20 – 24:44 | Ook als ik problemen heb – euuuhm. Joa dat is ook waar he. Ook als mijn familie/vrienden dat niet doen. Tja. Juist. Ik heb er moeite mee om doelen te stellen, joa dat ga ik niet rap doen. Waarschijnlijk wel. Plannen te maken om doelen te bereiken: joa waarschijnlijk wel. Ik hou mijn voortgang bij: als ik kijk op mijn horloge als ik 5km loop? **Neen als je een doel stelt of je bijhoudt of je er toe komt.** Ahja zo dus ik wil 5 km lopen in 30min.. **neen het gaat meer om het feit dat je zegt ‘ik ga elke week gaan lopen’ dat je bijhoudt of je dat wel doen**. Ah ja ja. In mijn gedachten wel, waarschijnlijk wel. Ik heb een duidelijk plan: dat is een rare vraag. Eens of oneens. Euuh. Als ik zou meedoen aan een wedstrijd heb ik wel een plan, maar helemaal oneens he dan? Ja. Duidelijk plan voor hoeveel ik ga bewegen. Mee eens. Ik heb een duidelijk plan voor wat ik ga doen als er iets tussen komt: mee eens. In situaties waarin het moeilijk is: daar is niet veel verschil met het vorig he. Ja mee eens. Volgende. |
| DEEL 1 ADVIES | 24:44 – 28 | 2 uur per dag, daar zit ik toch wel meer aan he. Maar ja dat is omdat je niet meer dan 2 uur kan invullen he. Als je kuist, beweging op je werk. Het maximum dat je kan zeggen is ‘meer dan 2 uur’. Bij mij op het werk zit je toch snel aan 6-7 uur. Ik ben tamelijk tevreden over dat resultaat. Ik vind het jammer dat dat daarbij staat van in de tuin werken. Stel nu dat je op een appartement woont, die kan dan wel veel huishoudelijk werk doen maar .. *(leest stukje van advies voor)* **dat zijn fictieve voorbeelden van de website.** Met de fiets naar het werk gaan, dat is iets wat ik ook kan doen maar dat gaat mijn beweging niet veel vooruit helpen omdat ik heel dicht tegen mijn werk woon. **Als het kan bij actieplan op ‘ja’ klikken.** Ja dat ging ik eigenlijk wel doen! |
| DEEL 1 OPSTELLEN ACTIEPLAN | 28 - 38 | Denk je dat je het in bepaalde situaties moeilijk zal vinden om meer te bewegen .. ja. Welke opties uit de volgende lijst kunnen voor jou lessen zijn: mag je er meerdere aanduiden? **Ja.** Gebrek aan tijd. Dat ook wel een beetje. Wacht eens. Dat ik verplicht ben om binnen 6 weken .. heb dat ik er tijd ga moeten insteken. **Maar dat is een gebrek aan tijd dan he? Of niet?** Ahja ja oke. Maar dat is iets dat erbij komt dat je anders niet hebt he. Tussen die twee he, als je wilt kan je ook lopen, ik ga die nemen. De grootste hindernis: zelfdiscipline. Wat kan je daarbij helpen.. je kan ook een sportpartner of sportclub zoeken. Duid het voorstel aan dat je zelf wilt gebruiken. **Je mag zeggen wat je daarbij denkt he.** Een sportpartner zoeken ja.. voor op mijn tempo te lopen.. de meesten lopen sneller dan ik en de sportclub is ook hetzelfde als je dan iedere keer aan de staart hangt is dat ambetant.  De beste beweging is van te sporten he niet van te werken.  **Het is 1.** En lopen staat er dan niet op he, het is joggen? **Ja**. Ik ben ook ingeschreven in tennislessen. Weet je al bij welke club, ja. Waar zal je joggen uitvoeren; is dat een stad? **Ja bijvoorbeeld.** Eigen gemeente ga ik schrijven. En tennis: TC. Hoeveel dagen per week: tjah.. ik wil dat 2 dagen. Ik ga 1 dag aanduiden. Op welke dagen: niet op dinsdag en donderdag. Euuhm. Ah ik mag meerdere antwoorden. Maandag, woensdag, zaterdag zondag. Tweede activiteit: dinsdag en eventueel vrijdagnamiddag. Soms dat, dat nooit, dat wel. Als het middag is, namiddag is dat kan. Als ik thuis ben na het werk, dat kan. Avond kan. Voor het het avondeten kan.  Als het namiddag is de vrijdag. Als het avond is, eigenlijk is dat het uur dat ik normaal zou tv kijken. 45 minuten. Tweede activiteit een uur. |
|  | 38 - 41 | Als-dan plan: euuhm. Als het weer het toestaat, ga ik joggen.… als ik niet te moe ben. **Je kan er ook meerdere maken**. Apart dan. Als het weer het toestaat, ga ik joggen als ik niet te moe ben. Als ik dan apart wil gaan oefenen moet ik een partner hebben om te tennissen. ‘… en mijn partner tijd heeft’. **Je moet zeggen vanaf vandaag anders klopt mijn programma niet.** Hier niets? **Neen.** |
| DEEL 1 ACTIEPLAN | 41 – 45:33 | Dat is hetgeen dat je kunt printen zeker. **Ja.** Ik ga dat naar mijn tennis partner mailen he, maar ik weet haar email niet vanbuiten. Moet ik het gaan halen? **Dat moet niet per se.**  Agenda, kalender … hier. Versturen. **Je hebt nu de eerste module doorlopen en normaal is de tweede na een week maar wij gaan die nu al doorlopen.** |
| DEEL 2 VRAGENLIJST | (2^e^ fragment)  Begint op 2:00 – 10 | *(leest vragen voor)* Het zou dus in die week moeten veranderd zijn? **Je moet je inbeelden dat je de eerste week hebt doorlopen.**  … ik woon echt niet op een locatie waarvan je zegt ‘k ga wandelen’. Niet meer dan 2 dagen .. meer dan 2 uren. Fietsen.. welk onderscheid had ik de vorige keer gemaakt he .. **zwaar is wat voor jou zwaar is**. **Lopen heb je bij zwaar gezet en matig heb je geen ingevuld.** Ja juist. 2 dagen. 1 uur. 2 uur. Dat doe je toch dagelijks he. Pak vier dagen. Dat gaat dan over die trappen he? Dat is 10 minuten he. **Aan een stuk he staat er op**. 2 dagen. Als je dat zo kan omschrijven he.. je stapt en je staat dan weer ergens stil, en dan stap je door maarja. Dus meer dan 2 uur. Als ik gelopen heb deze week.. dan denk ik .. dat ik het wel ging gedaan hebben. Ik denk wel dat ik 1x zou gaan lopen zijn. Hier moet je ook veronderstellen dat je? – planning hé maar dat is ook pas na Pasen dat dat doorgaat. **Laten we veronderstellen dat het al gestart is.** Ja. Sowieso is dat 60 minuten. |
|  | 10 – 19 | Wat was de reden .. door bepaalde omstandigheden. Het eerste is gewoon van 30 minuten te lopen en wil dit langzaam opdrijven. Tennispartner is nog op reis voor 2^e^ spel tennis.  Waarom doel niet kon behalen; dat is toch weer hetzelfde he? Het ene is de partner dat ik niet heb en het ander is omdat ik het voor mijzelf heb uitgemaakt dat ik het niet wil om daar vijf te doen. **Ja. Als je niet akkoord bent kan je dat er nog altijd bij zeggen he.** Ik kan natuurlijk wel aan iemand anders vragen om te tennissen. Ik ga dat dan doen. Hoe wil je meer bewegen: in mijn vrije tijd. Weet je al op welke plaats bij welke club: ja. Eigen gemeente. **Wat denk je bij dat onderdeel nu?** Ja dat het dezelfde vragen zijn als dat vorige. De locatie gaat niet snel veranderen he.. dus dat is eigenlijk overbodig. Denk ik. Hoeveel dagen per week: 1 dag in de week, dat is ook dezelfde vraag. De maandag, de woensdag, de vrijdag, zaterdag, zondag. Dat zijn allemaal dezelfde vragen. Niet als het ochtend is maar in de voormiddag, als het middag is, namiddag, avond .. dat is ook nog een keer hetzelfde dan. Tweede activiteit.. als het weer het toelaat en de tennispartner kan. |
| DEEL 2 AANPASSEN ACTIEPLAN | 19 – 28 | Dan het actieplan, weer hetzelfde als mijn eerste he. Daar ga ik niet veel aan veranderen he. In die week. **En nu wachten we weer op een mail want er is een module die normaal naar 1 maand komt maar we gaan die nu maken.** |
| DEEL 3 VRAGENLIJST |  | ….  1 dag onkruid uittrekken.. ik vind het jammer dat er geen optie tussen is van de maand bijvoorbeeld. Dat zal niet meer dan 20 minuten zijn. 2 dagen. Dus trappen 4 dagen. **Wat denk je bij dat stuk?** Wel ja dat ‘als-dan’ hebben we al gemaakt he .. |
| DEEL 3 REST |  | **Ik ga nu nog 3 meer gericht vragen stellen. Welke zaken vond je achteraf gezien echt goed? In het algemeen dan he.** Dat je dingen moet vooropstellen. Dat je tijd en dagen moet vooropstellen. **En zijn er dingen die je slecht vond?** Dat je bijvoorbeeld van je werkuren maar 2 uur kunt aanduiden. Dat je niet die activiteiten doet in een week dat er eens een week voorbij gaat waar je niet helemaal, het is makkelijker te beoordelen in een maand. **En vond je dat gemakkelijker om over na te denken na een maand zogezegd?** Ja. **En wat zijn volgens jou de belangrijkste punten ter verbetering?** Hetgeen ik gezegd heb, die herinneringen dat ze sturen via mail dat vind ik ook wel goed. Als je echt van plan bent om meer te bewegen, en als je dat dan nog eens via sms aan herinnerd wordt, dan ga je dat veel rapper doen. |
