# Supplementary material for: Users’ thoughts and opinions about a self-regulation-based eHealth intervention targeting physical activity and the intake of fruit and vegetables: A qualitative study
Source: PLoS One. 2017 Dec 21;12(12):e0190020. doi: 10.1371/journal.pone.0190020 (PMC5739439; doi:10.1371/journal.pone.0190020)
Supplement: S3 File — This file contains the transcribed interviews. (ZIP) [file pone.0190020.s003.zip › general_population/TA1BESS.docx]

**Code filmpjes:**

| Deel interventie | Minuten | Transcript |
| --- | --- | --- |
| DEEL 1  VRAGENLIJST | 0-10 | ik zal beweging kiezen. Drie onderdelen. Ja. Hoe heet je .. *(vult persoonlijke gegevens in)*  activiteiten in en rond het huis, dat is kuisen enzo? **Ja**.  Fiets ik 10 minuten aan een stuk, neen het zijn er 7. En ik mag dat niet optellen he want het moet aan een stuk zijn. Hoeveel fiets ik .. dat hangt er van af he, soms is dat 10 soms 20. **Dan mag je een gemiddelde nemen.** Op welk tempo fiets je dan, middelmatig. ’s Morgens is dat snel maarja, het telt niet mee want dat is maar 7 minuten he! Boodschappen doe ik met de fiets. **Als er zo dingen in u opkomen dat mag je gerust zeggen.** Minstens 10 minuten ja .. ik vind kuisen toch wel een matige fysieke activiteit maar niet dat je daarvan dieper of sneller inademt he.. maar ik vind dat voor mij .. ja. Dan toch maar op 1 dag. Tuin of moestuin .. dat zal 30 minuten zijn. Zware activiteiten in de tuin, dat is niet aan mij besteed. |
|  | 10-19:25 | Matige activiteit.. hmm moet dat ook achtereenvolgens zijn? Ik stop wel eens tussendoor. **Neen pak maar in totaal.** Stofzuigen, pakt dat dat 10 minuten is, dan dat erbij, dan den boven, anderhalf uur tohc he. Sport als recreatieve activiteit.. 2 dagen. Zware fysieke activiteiten.. wat was dat ander dan? Ahja matig. Ik vind die beweging toch vrij intensief dus ik twijfel of ik dat nu bij zwaar of matig moet zetten. Ik ga het dan op 1 dag zetten. Dat wandelen is dan wel wat langer. Die andere dan is dan wel 2 keer. Oei, dat is nog iets anders? Ahja wandelen staat hier niet tussen. 1 keer. Ik ga die fietsen hier moeten gaan aanpassen hé. Als je wandelt in je vrijetijd.. welk tempo. Ik stap toch wel door. Matig.  Tijdens je werk. Als ik de voetbal erbij reken dan wel. Maar in mijn dagelijks werk niet eigenlijk. Hoeveel beweeg je denk je .. hmm. Tussenin. Ben je van plan meer te bewegen, als het beter weer begint te worden dan wat meer wandelen en fietsen, waarschijnlijk wel. Hoe moeilijk of makkelijk denk je dat het is meer te bewegen, tussenin. Als ik meer beweeg is mijn kans op het ontwikkelen van ziektes kleiner: helemaal juist. Zal ik mij mentaal beter voelen ja. Zullen anderen mij steunen, als ik het laat weten aan anderen wel ja! Ik ben er zeker van dat ik meer kan bewegen dan dat ik nu doe, ja dat is waar. Ook in moeilijke situaties, dat is juist. Ook als ik telkens opnieuw moet proberen, euuuhm.. ahja wacht, het is meer ‘kan’. Dat ik in staat ben om? **Ja.** Hm ja. Meer kan bewegen ook als ik problemen en zorgen heb, ja. Ook als familie/vrienden dat niet doen ja. Ik heb er moeite mee om doelen te stellen. Dat doe ik wel. Plannen te maken.. ik hou mijn voortgang bij. Joa. Ik heb een duidelijk plan.. het is nog een beetje vaag. Het is altijd afwachten met het weer. Voor hoeveel ik ga bewegen, dat niet he. Als er iets tussen mijn plan komt, daar ben ik nog niet mee bezig. In situaties waar het moeilijk is, ik denk dat ik te vlug ga toegeven dat het niet gaat. |
| DEEL 1 ADVIES | 19:25 – 21:10 | *(leest advies voor)* en mag ik nu kiezen of ik een actieplan maak? **Het is eigenlijk de bedoeling dat je er een maakt ja!** Haha oke! |
| DEEL 1 OPSTELLEN ACTIEPLAN | 21:10 – 34:40 | In bepaalde situaties moeilijker zou vinden.. joa. Hindernis.. haha! **Waarom lach je?** Ja fysieke hindernis dacht ik he, dat sowieso al. Gebrek aan tijd, dat is een smoesje he. Het is de bedoeling iets te kiezen dat je graag doet he. Slecht weer kan natuurlijk .. gebrek aan sportpartner, neen ik heb er geen probleem mee alleen iets te doen. Blessure ja. Welke optie is de belangrijkste.. ahja dus hier waren het alle en nu de belangrijkste? En dat mogen er verschillende zijn ahja. De externe factoren en de blessure.. ah neen het mag er toch maar 1 zijn!  Ik heb bijvoorbeeld bij het zwemmen een krak gehad in mijn schouder en daardoor heb ik het zwemmen laten liggen al 2 maand, want ik ging ook 1x in de week zwemmen. Actieve levensstijl .. bwa in de vrije tijd toch. American football haha ja dat is helemaal mijn ding! Ja mag toch een ding dan, dan ga ik wat scrollen! We gaan het bij fietsen houden. Het wandelen mag er ook bij. Dat stond er niet tussen.. **je kan nog naar beneden scrollen**. oei ik heb nog niet alles gezien! Weet je al waar je deze activiteiten kan doen.. wandelen ook best ja he. Op straat. Waar .. moet dat echt een plaats zijn? **Je kiest zelf.** Waar zou ik eens gaan fietsen .. Waar het plat is! Meetjesland. Tweede activiteit was het wandelen.. dan gaan we naar het bos. Hoeveel dagen per week, we beginnen met 1 dag in de week, alleja nog 1 dag extra. En de tweede ook. Gaat het hier specifiek over volgende week of altijd? **In het algemeen.** Oke dan fietsen op vrijdag en wandelen op zondag. Wanneer .. hmm. Eerst nog een beetje wakker worden hé. In de voormiddag ben ik het meest fris. Hoe lang.. toch iets langer dan 60.. we zullen het dan maar een beetje verminderen.. 45. Wandelen doe ik wel 60min. Als-dan. **Je frontst zo**? Ja ik ben niet direct mee.. aahja! Als ik klaar ben en ontbeten heb, dan vertrek ik hé. Voor mijn tweede activiteit.. weer hetzelfde. Als ik ontbeten heb, |
| DEEL 1 ACTIEPLAN | 34:40 – 36:35 | Dat is een koddig tekeningetje! **Waarom lach je?** Omdat het zo mooi uitgeschreven is! Het is leuk voorgesteld. Mijn als-dan plan is niet zo spectaculair maar bon. Ik wil het niet per se tonen.  In een boekje is goed. Tijd voor actie! Ja. Versturen. |
| DEEL 2 VRAGENLIJST |  |  |
| DEEL 2 AANPASSEN ACTIEPLAN |  |  |
| DEEL 2 REST |  |  |
