# Supplementary material for: Users’ thoughts and opinions about a self-regulation-based eHealth intervention targeting physical activity and the intake of fruit and vegetables: A qualitative study
Source: PLoS One. 2017 Dec 21;12(12):e0190020. doi: 10.1371/journal.pone.0190020 (PMC5739439; doi:10.1371/journal.pone.0190020)
Supplement: S3 File — This file contains the transcribed interviews. (ZIP) [file pone.0190020.s003.zip › general_population/TA1BETE.docx]

**Code filmpjes:**

| Deel interventie | Minuten | Transcript |
| --- | --- | --- |
| DEEL 1  VRAGENLIJST | 0-9:10 | *(leest inleiding).* Ik denk dat ik fruit ga nemen, want dat is het meest dramatisch. *(vult gegevens in).* Hoeveel dagen heb je de voorbij week fruit gegeten. Goh, dat hangt er een beetje van af. Vorige week heb ik meer fruit gegeten dan deze week. Mag ik dan het gemiddelde nemen? Ik ga toch een beetje het gemiddelde nemen. 2 dagen. Appels en peren 2. 0, aardbeien is apart zeker. 1, 0. Mandarijntjes .. het is de tijd niet van de mandarijntjes. Kiwi’s neen. Schijven meloen 0, lychees 0, oei .. ik heb niet zoveel fruit gegeten. Andere soorten vers fruit .. eetlepels? Waarom is dat? Fruit uit blik of glas. Fruitmoes, neen. Hoeveel fruit denk je dat je eet? Weinig. Ik vind dat wel nog lekker maarja, ik vergeet dat ook om te eten, je kan dat niet als maaltijd eten.  Ik denk dat het makkelijk is als je er op let.  Ik denk dat het allemaal wel juist is, ik denk wel niet dat andere mensen mijn gaan steunen. Mentaal wel, omdat ik dan denk dat ik gezond bezig ben. Ik denk wel dat het gezonder is dan een koek ofzo tussendoor. Hoe bedoel je over de vragen? **Of je het raar gesteld vindt ofzo..** euhm .. neu. Niet echt eigenlijk. Meer fruit in moeilijke situaties.. maar weinig fruit beschikbaar? Er is toch altijd fruit in de winkel? Of te weinig tijd .. meer fruit kan eten ook in moeilijke situaties .. ik zo zeggen ‘neutraal’ maar die optie is er niet. Ik ben daar niet zeker van ik denk fout. Meer fruit als ik telkens opnieuw moet proberen tot het me lukt, opnieuw mee beginnen dan? Dat vind ik niet duidelijk die vraag, kan je dat overlaten? Neen zeker. . ik ga juist doen he, ik weet niet wat ik daarop moet antwoorden. Als ik proberen en zorgen heb .. ik denk dat je daar niet aan denkt dan. Ook als vrienden/familie het niet doen: dat denk ik wel. Ik heb er moeite mee om mezelf doelen te stellen: maar ik stel mij eigenlijk geen doelen. Hmm .. pff ik weet dat niet. Neutraal. Ik heb er moeite mee plannen te maken: maar ik maak geen plannen. Dus kheb er wel moeite mee denk ik dan? Ik vind dat een rare vraag over dat plannen. Want ik denk niet dat veel mensen dat plannen om fruit te eten.  Ik heb geen fruitdoelen, dus zeker niet dan.  Ik heb een duidelijk doel: helemaal mee oneens. Ik heb helemaal geen plan! Ik vind dat raar dat je zou plannen wanneer je fruit eet. |
| DEEL 1 ADVIES | 9:10- 10:30 | *(leest advies voor).* Elke dag twee porties, ja ik weet het. Een persoonlijk actieplan pfff …. **Je zou ja moeten zeggen**. Ahja oke, maar eigenlijk zou ik neen zeggen. **En waarom dan?** Euhm, omdat ik dat een beetje banaal vind dat je fruit zou inplannen, dat moet je gewoon eten wanneer je zin hebt in fruit. Je kan er wel op letten dat je tegen de maaltijd minder eet en dan erna of ervoor nog een stuk fruit eet, maar ik denk dat ik me er toch niet aan zou houden aan zo een plan. |
| DEEL 1 OPSTELLEN ACTIEPLAN | 10:30 | Ik denk wel dat ik dat niet zou volhouden eigenlijk. Ik vergeet gewoonweg om fruit te eten. Ja nee, ik vergeet het gewoon echt. Hoe wil je proberen meer fruit te eten dan nu.. enkele voorstellen. Euuuhm. Ja ik denk door dat klaar te leggen. Maar dan mag je het ook weer niet vergeten he. en dat van die plaats, het is een gewoonte dat het daar staat, daar kijk ik eigenlijk niet meer naar. Dan loop je daar sneller aan voorbij.  Toch wel 5 dagen per week. Dat zou beter zijn dan nu.  Waar will je, WAAR.. dat is mij al gelijk ze. Ik zal ze allemaal aanduiden. Dat doet er volgens mij niet toe waar je dat eet. En wanneer, dat doet er ook niet toe. Ik denk dat het belangrijkste is dat je het eet. ‘als-dan’: als ik niet vergeet om mijn fruit klaar te leggen, dan zal ik het meenemen naar school. En als ik er aan denk om fruit te kopen, dan zal ik het klaarleggen op kot. Ik weet niks meer. Vanaf wanneer, vanaf vandaag zeker? **Ja dat mag je aanduiden.** |
| DEEL 1 ACTIEPLAN | 15:30 | Dat is eigenlijk gewoon een samenvatting? BMI.. euuuh .. **dat is omdat je een kommagetal hebt ingegeven, dat is een foutje.** Van die twee porties, dat wist ik al he, ik denk niet dat ik naar de dokter ga gaan daarvoor. Ik wil dat niet echt bijhouden, ik denk dat het gewoon het belangrijkste is dat dat een beetje een routine wordt. Ik denk dat ik het zou vergeten aanduiden gewoon, of de dag zou verwisselen. Plan aan iemand ander tonen.. neen? Ik denk niet dat mensen geïnteresseerd zijn in hoeveel fruit ik eet per dag! Moet ik het versturen? Ja. Oke. |
| DEEL 2 VRAGENLIJST | (2^e^ fragment) | Dus nu zijn we zogezegd een week later? **Ja.** Ik vind dat lettertype zo groot, ik vind dat niet zo aangenaam, een beetje aanvallend eigenlijk. Dat zou wel wat kleiner mogen.  …  Ik zal zeggen 4 dagen dat ik toch mijn best gedaan heb he. ik vind die onderverdeling ook wel wat raar.. dessert schaaltjes, niemand doet dat toch in schaaltjes? En wat zijn sweeties? Ik snap de onderverdeling niet goed waarom dat druiven apart staan, meloenen, mango’s, en dan zo bessen maar dan staat aardbeien alleen. En fruitsla is een mengeling van alles. Appels en peren wel 3. Dessert neen, daar hou ik mij niet mee bezig, het is gewoon een stuk fruit. Aardbeitjes heb ik ook gegeten een keer. Abrikozen eet ik niet zo graag. Vijgen, joa ik vind dat wel lekker maar ik koop dat gelijk niet. Een hand vol… je hebt mensen die grote handen hebben en mensen die kleine handen hebben. 75g oke, maar ik ga dat wel niet wegen denk ik. Nectarine heb ik ook niet gegeten. Schijven meloen. Schijven? Wij doen dat niet in schijven. Trosjes druiven, bedoelt ze dan een hele tros? Je gaat er toch niet meer dan 1 per week eet als je dat eet, tenzij dat je de hele dag druiven eet. Eetlepels andere soorten vers fruit .. niet dat ik me herinner. Fruit uit blik ook niet. Fruitmoes, neen. |
| DEEL 2 AANPASSEN ACTIEPLAN | 4 min | Ja dat is gewoon een te grote sprong van 2 dagen in de week naar 5, misschien was mijn doel inderdaad een beetje te hoog. Ik wil nog altijd mijn huidig doel en zo goed mogelijk verder doen tot ik het haal. En dat dan volhouden. |
| DEEL 2 REST |  | **Wat vond je van het tweede deel?** Ik vind het eigenlijk niet echt nodig, ik denk niet dat de mensen de website nodig hebben om aan die website te vertellen ik heb dat en dat gedaan. Ik heb dat toch niet nodig. Ik denk ook niet dat dat veel invloed zal hebben om toch mijn doelen te bereiken, om toch die 5x fruit per week te eten. Dat is mijn mening. Ik vul ook niet graag enquetes in, ik vind dat een beetje tijdverlies en dat dat niet echt zin heeft voor mezelf. Ik denk niet dat ik het een tweede keer zou invullen. Ik denk dat ik er meer aan zou hebben om een website te hebben met meer tips enzo snap je? Meer informatief zou ik aangenamer vinden. |
