# Supplementary material for: Users’ thoughts and opinions about a self-regulation-based eHealth intervention targeting physical activity and the intake of fruit and vegetables: A qualitative study
Source: PLoS One. 2017 Dec 21;12(12):e0190020. doi: 10.1371/journal.pone.0190020 (PMC5739439; doi:10.1371/journal.pone.0190020)
Supplement: S3 File — This file contains the transcribed interviews. (ZIP) [file pone.0190020.s003.zip › general_population/TA1BETH.docx]

**Code filmpjes:**

| Deel interventie | Minuten | Transcript |
| --- | --- | --- |
| DEEL 1  VRAGENLIJST | 0-7:45 | Eet jij voldoende porties fruit per dag? *(leest richtlijnen)* Ik heb geen advies nodig, ik weet perfect wat ik moet doen, maar ik doe het niet. Dus .. enteren of moet ik op iets klikken? **Je moet een module kiezen.** *(vult persoonlijke gegevens in)* geen diabetes, geen dieet. Volgende. Hoeveel dagen heb je de voorbije week fruit gegeten. Haha, geen enkele! 1 keer appelmoes. Heel weinig. Waarschijnlijk wel want we gaan op reis dus ga ik meer tijd nemen om te eten. Als ik meer fruit eet is mijn kans op ziektes kleiner. Waarschijnlijk wel. Zal ik mij mentaal beter voelen. Neen. Zullen anderen mij steunen, neen. Ik ben zeker dat ik meer fruit kan eten, ja. In moeilijke situaties: weinig tijd .. pff. Ja. Kan eten als ik telkens opnieuw moet proberen (blaast). Neeje. Ik denk daar allemaal niet aan. Ook als ik zorgen heb, daar eet ik geen fruit voor. Wacht. Helemaal fout. Meer fruit kan eten ook als mijn familie en vrienden dat niet doen. Nee. Voor mezelf doelen stellen.. zeker wel. Ik koop ze, maar ik eet ze niet op. Ik heb er moeite mee plannen te maken, ja zeker wel. Ik hou mijn voortgang bij, baneen. Hup. Ik heb een duidelijk plan, neen. Iets tussen mijn plannen komt.. helemaal niet. Seg het is blijkbaar de derde wereldoorlog .. een plan maken om fruit te eten man man man! De voorbije week geen fruit gegeten.. ja. |
| DEEL 1 ADVIES | 7:45-8:40 | *(leest advies voor)* oh my god. Dat een mens hem daarmee kan bezig houden. Neen ik wil geen plan maken. **Je moet op ja klikken.** Pfff… allee dan omdat het voor u is. Miljaar! |
| DEEL 1 OPSTELLEN ACTIEPLAN | 8:40 | Met wie je meer fruit wil eten?? Denk je dat je het moeilijk zal vinden, ja ben ik helemaal zeker van. Ja. Welke opties – ik vind veel fruitsoorten niet lekker dat is 1, ik vind fruit duur neen dat is het niet, gebrek aan steun zeker niet ik vind dat fruit niet lang genoeg houdbaar is neen, ik bereid fruit niet graag ja dat is waar. Ik heb te weinig tijd, ja. Ik weet niet wanneer ik fruit kan eten neen.. neen. Ik vergeet vaak ja. Voila. Welke optie is je belangrijkste hindernis: het is nog eens hetzelfde? Wat is het verschil? **Het is nu enkel de belangrijkste.** Ik vind veel fruitsoorten niet lekker. Ik heb te weinig tijd. Hoe wil je proberen; een nieuw gebit? Voorgesneden diepvriesfruit, daar zitten geen vitaminen in, daar zitten meer suikers in. Blikfruit is ook niet goed. Neen. Op voorhand jah .. door 6 soorten te kiezen die je niet hoeft te bereiden. Aan de hand van .. pfffff .. om de 2 dagen. Volgende. Waar wil je fruit eten? Thuis. Wanneer. Tijdens het avondmaal.  Als-dan plan: **wat denk je ik zie je zo fronsen?** Pfff .. als ik nog honger heb, dan neem ik fruit als dessert. **Zeg maar wat je denkt;** wat ik denk, dat dat allemaal goed is op papier maar … **je moet schrijven vanaf vandaag anders blokkeert het systeem.** |
| DEEL 1 ACTIEPLAN | 13:30 | Te bespreken met je huisarts.. ah voila! Bananen dat eet ik nu wel eens. Ik ga dat allemaal niet bijhouden, ik heb mijn brain. Ik wil dat niet bijhouden. Ik ga dat onthouden, ik heb geen boek of agenda kalender allez .. neen. Ik ga dat niet doen he, ik ben geen klein kind. Actieplan is klaar. Versturen. |
| DEEL 2 VRAGENLIJST |  | Nog eens fruit ?? **ja nu is het alsof je het al een week hebt geprobeerd.** Ik heb daarnet mijn email enal ingevuld … **ja maar nu is het zogezegd een week later.** Ah oke. **Begrijp je deze module?** Neen. **Je moet doen alsof je het plan een week hebt gevolgd.** Ik ben dan aan het liegen he. **Ja maar dat maakt niet uit.** **Het is vooral wat er in je opkomt bij het doornemen van de module.** Dus schaaltjes, neen. Aardbeien niet. Vijgen niet. Handen vol kersen, neen. Kiwi’s neen. Lychees ook niet. Mango ook niet.  **Zeg maar wat er in je opkomt.** 1 keer appelmoes.  Zelfde doel.. ik lust geen fruit dus. Wat ga ik doen. Pff wéér dat zelfde. **Dat is omdat je hetzelfde doel heb ingegeven.** |
| DEEL 2 AANPASSEN ACTIEPLAN |  | Email adres, weeral. Telefoon. Volgende. Heb je de vorige keer een actieplan gemaakt, ja. **Het is nu na een maand he.** Ik ga helemaal hetzelfde terug invullen. Appels peren bananen. Dus schaaltjes, neen. Aardbeien niet. Vijgen niet. Handen vol kersen, neen. Kiwi’s neen. Lychees ook niet. Mango ook niet. Waarom staan mandarijntjes en pruimen samen? |
| DEEL 2 REST |  |  |
