# Supplementary material for: Users’ thoughts and opinions about a self-regulation-based eHealth intervention targeting physical activity and the intake of fruit and vegetables: A qualitative study
Source: PLoS One. 2017 Dec 21;12(12):e0190020. doi: 10.1371/journal.pone.0190020 (PMC5739439; doi:10.1371/journal.pone.0190020)
Supplement: S3 File — This file contains the transcribed interviews. (ZIP) [file pone.0190020.s003.zip › general_population/TA1BETS.docx]

**Code filmpjes:**

| Deel interventie | Minuten | Transcript |
| --- | --- | --- |
| DEEL 1  VRAGENLIJST | 0-10 | Ik ga bewegen kiezen. *(vult gegevens in)* ik heb dat al eens ingevuld.. Ik ga er dat op zetten maar ik weet het eigenlijk niet goed. **Als het ongeveer is dan is het goed he.**  …  **Je mag verwoorden dat wat er in je opkomt als je dat leest.** Tgoh ja, ik vind het nogal moeilijk die ‘zware’ en die ‘matige’ waar je de lijn eigenlijk trekt .. en dat is ook voor iedereen een beetje anders he. Ik werk soms in de tuin en als ik 5 uur in de tuin werk ben ik ook moe he.  Ik ga er 6 van maken, ik gebruik mijn fiets wel heel veel. Wacht hé. Ik ga 50 minuten pakken, dat is ook een gemiddelde hé? Goh.. ja. wacht he. Wandelen doe ik niet echt veel. Het ging over, voor het werk of – ontspanning he? Ik moet nu wel zeggen, ik ga 3x in de week de kindjes halen, en dat doe ik te voet. Dat mag daarbij dan? **Is dat dan meer dan 10 minuten?** Ja dat zal meer dan 10 minuten zijn! **ja dat mag daarbij**!  Dat snap ik hier niet goed, hoelang wandel je normaal op zo een dag om ergens heen te gaan en terug te keren.. ahja! Dat gaat over die minuten .. ja 20. Goh.. middelmatig tempo. Ik moet nu wel zeggen dat ik heel weinig structuur heb. Omdat ze hier spreken van een gewone week, maar deze week kan totaal anders zijn dan vorige week. Ik ga niet zeggen dat ik geen structuur heb maar wel zeer weinig. Dus voor mij is het moeilijk om daar een gewone week van te maken. **Dan zou ik zeggen dat je een beetje gemiddeld rekent..** dat gaat eigenlijk .. maar ze schrijven hier ‘tuin of moestuin’ maar mag dat ook iets anders zijn? **vul maar in wat je er zelf onder verstaat.** Hoeveel dagen .. ik ga daar toch 3 van maken. Hoeveel tijd.. moeilijk he, maar we gaan er een uur van maken. Daar ga ik toch wel 1 dag van maken. Mag ik dat ervan maken? **Ja het is hoe je het zelf interpreteerd!** Ik ga er twee dagen van maken. |
|  | 10-22 | Ik vind dat dikwiljs moeilijk, je doet wel iedere dag iets.. de was ophangen, ruiten kuisen, allez ja. hoeveel tijd .. hmmm. En boodschappen valt dat er ook onder? **Als je vindt dat dat voor jou even zwaar is als bijvoorbeeld ruiten kuisen dan mag dat er ook bij**. Ja wacht he. Twee dagen. ik probeer 2 keer in de week te gaan joggen en 1 keer te gaan zwemmen. Het zijn er die vinden dat dat al veel is, maar hier wil ik eigenlijk ‘geen’ want voor mij is dat niet ‘kweetnie hoe zwaar he’. **Lopen en zwemmen staat er wel bij als voorbeeld, maar je kan het ook later invullen.** Ik weet niet goed wat ik eigenlijk moet invullen.. voor mij persoonlijk is dat geen zware fysieke activiteit. Maar voor andere mensen misschien wel. **Ja dat is een goeie opmerking.** Ik ga er 1 dag van maken. Wandelen is dus met een wandelgroep he? **Ja of op jezelf maar niet echt om ergens naartoe te gaan**. dat doe ik soms , maar niet iedere week.  Wandelen in het stad moet ik dat daarbij rekenen? **Als je dat doet als ontspanning mag dat daarbij**. Ik vind dat het zo een beetje te weinig gespecifieerd is zo. Voor mij wandelen vanaf 2 uur, anders vind ik het geen wandelen he. Maar je kan ook wandelen in het stad dat je gaat shoppen, maar dat is een ander soort wandelen he. **Ja ik begrijp wel wat je bedoelt.** in het stad gaat dat een laag tempo zijn. maar als ik ga wandelen om te wandelen gaat dat een hoog tempo zijn. dus wat moet ik nu invullen? **Doe maar wat je denkt. Dat is zeker een goede opmerking.**  ik ga onbetaald opschrijven .. ik vind toch dat ik veel beweeg.  De volgende maand meer bewegen.. de komende maand is nogal druk. Ik ga opschrijven ‘waarschijnlijk niet’. Minder zal het allesinds niet zijn. als ik meer beweeg dan.. dat is gewoon wat ik ervan denk? **Ja inderdaad.** Goh .. hier wil ik eigenlijk noch ja noch nee op antwoorden. Hier zou ik ook graag iets in het midden willen zetten terug. Ik zou eigenlijk hiertussen willen maar die optie is er niet. Dat is zeker juist. Dat is ook zeker juist. Dat eigenlijk niet neen. Ik heb blij dat ik dat gekozen heb, ik ging zeker moeite gehad hebben met die groenten en dat fruit ! dat ga ik hier zo doen.  Ik ben er niet zo mee bezig, soms wil ik 2 keer joggen en 1 keer zwemmen maar nu in mei als ik veel simulatie patient moet zijn komt dat er niet van. Soms ben ik dan geambeteerd dat het er niet van komt, maar dan heb ik wel iets anders gedaan snap je. Dus ja, je mag niet overdrijven. Als ik een uur fiets naar het UZ heb ik niet gelopen maar wel meer fietsafstand gedaan.  Ik heb een duidelijk plan .. hier staat er weer niet bij .. noch het een nog het ander he; dan ga ik ‘neen’ zetten. Ja.. dat is niet echt, dat is zoiets wat ik daarjuist vernoem eigenlijk. Hetgeen ik doe heeft heel dikwijls met beweging te maken. Bijvoorbeeld tuinwerk noem ik ook beweging. Goh. Ik lees ook graag in de boekjes. Maar daarvoor ga ik mijn beweging niet laten he. |
|  | 22 – 23:45 | Ik vind het nogal moeilijk, ik moet nu kiezen dit of dat maar het is eigenlijk ertussen. **Dat is vaak het probleem he?** Ja. **het doet er ook niet zoveel toe wat je invult, het is vooral wat je erover zegt dat belangrijk is.** Oke ik ga nu dat kiezen. Dat is goed he. |
| DEEL 1 ADVIES | 23:45 – 30:53 | *(leest advies voor)* ja, dat lijkt zo veel maar.. dat zou ook wel kunnen ja. ik heb ook geen auto he, dus ik moet alles met mijn fiets doen he. Haha.  …  eigenlijk wil ik dat actieplan niet maken. **Ja, wil je dat misschien maken voor een fruit of groenten? Omdat je zegt ik beweeg al veel** .. Dat is misschien een idee! Dat kan ik mij inbeelden. Voor dat wil ik dat niet maken want ik wil 2x joggen en 1x zwemmen en dan fiets ik veel maar ik ga daar geen structuur in maken, soms is dat wat minder en soms wat meer maar geen structuur er in. Dat is niet nodig voor mij. **Misschien moet je dan op het laatste klikken**. **Nu mag je op versturen klikken.** |
| DEEL 1 OPSTELLEN ACTIEPLAN |  | / |
| DEEL 1 ACTIEPLAN |  | / |
| DEEL 2 VRAGENLIJST | (2^e^ fragment)  0-10  (3^e^ fragment)  0 – 15:35 | DEZE PERSOON SCHAKELDE OVER NAAR MODULE ‘GROENTEN’.  Zitten er groenten in lasagne bolognaise? **Ja soms wortels, ajuinen, tomaten..** ik zal er 4 van maken. Wat moet ik nu invullen, hoeveel schijfjes ik gegeten heb? Eetlepels niet .. dat eet ik niet zo dikwijls. Hoeveel wortels, 5. Komkommer eet ik niet.  …. Dat heb ik nu wel uitzonderlijk deze week eens gegeten. Gekookt witloof, of spinazi .. witloof in hesp dat durf ik wel eens klaarmaken. **Ja dat zit daar ook bij.** Ik wou 250g eten en ik kom minder uit, dus mijn plan is absoluut niet geslaagd he. Ik ging hier net op antwoorden van ‘eigenlijk wil ik geen doel’ ik wil dat ik af en toe eens groenten eet maarja. **Ja dan kan je aanduiden niet voldoende gemotiveerd om echt een doel te hebben.** Ik ben ook niet bezig met men kilo’s. … we gaan eens verder kijken.  Bonen zijn geen groenten?? **Ja raar he**. Die lust ik nochtans he.  ….  Als je soep eet, zijn dat ook groenten? **Dat beslis je eigenlijk zelf.** Ik zou dat er allemaal bij nemen. En pesto is dat ook een groente? **Ik denk het niet.** **Dat zijn meer kruiden.** Dat zou ik willen overslaan als het mag.. dat gaat niet.. **ik zal het even doen**. Ja doe jij het maar. Hoeveel groenten denk je dat je eet, weinig. Dat gaat wel gemakkelijker. Ik ga bij alles juist zetten want het gaat te lang duren anders! **Ja dat is goed.** Mijn wortel die ik iedere dag eet, valt dat er onder? **Ja.**  We gaan dat kiezen. Ik kan dat hier verzetten he? Ja. dat is ook wel juist, maar ik kan er iets anders boven zetten.. ja. dat komt er zeker boven. Euhm .. dat is eigenlijk, ik ga dat er boven zetten.  Er zijn te weinig groenten beschikbaar maar dat is omdat ik ze zelf niet meebreng he.  Ik bereid het niet graag dat is waar, maar ik ga een zakje gekuiste sla kopen, maar dat is te veel voor mij.. en dan ga ik er geen kopen want ik ga het toch niet allemaal opeten. **Dat is nog een andere hindernis?** Ja. **dat mag er zo bij.** Ahja oke.  Ik ga voor dat kiezen, dat doe ik ook soms. Blikgroenten dat is absoluut niet goed. Goh eigenlijk vind ik, moeten .. aan die vragen wordt je wel gewaar dat we ons soms laten leiden door wat er in de media verschijnt. Of dat groenten nu echt per se iedere dag moet, zoals ze schrijven ? dat betwijfel ik een beetje. Ik laat mij niet echt leiden door media, maar toch hier.. ik heb zoiets van ja je moet iedere dag groenten eten, maar is dat wel zo.. toch 4 dagen.  Ik heb ook niet zo een idee van hoeveel groenten wegen. Een tomaat, hoeveel weegt dat? **Dat vind ik zelf ook moeilijk hoor.** Aanbeveling is 300 gram maar ik weet niet wat ik mij moet voorstellen bij 300 gram. En de ene groente weegt ook meer dan de andere hé. Ik ga 200 invullen. Op die dagen. kom we maken er 250 van. Ik werk niet maar soms als ik ergens ben, mag ik dat ook? **Ja.** ik ga andere aanduiden. Ah maar ik mag maar eentje aanduiden? **Neen er staat meerdere antwoorden mogelijk.**  ….  **Je zet het best op de dag vandaag.** |
|  |  | Ik ga hier op volgende klikken. Dat opschrijven, dat vind ik nogal nutteloos. Nie omdat het over groenten of fruit gaat, maar ‘ik ga dat bijhouden in mijn computer’ , ik vind nu niet dat het zo slecht is dat ik heb moet gaan bijhouden. Ik kan mij wel een beeld vormen van deze week. Ik mankeer eigenlijk niets he. Maar in excel hou ik wel bij wat ik heb bijgehouden in mijn visa, maar dat kan je ook als tijdverlies zien he. Ik werk wel graag met cijfers, en dan zie ik het wel, maar van mijn groenten .. sorry neen. **Ja dat is niet erg, ik denk dat het er bij staat.** Ik ben in die apps nog niet thuis, waarschijnlijk ga ik er wel mee beginnen, maar voor zo zaken, neen. Goh .. ik wil dat wel tonen aan iemand maar dat blijft gewoon bij tonen he.  ….  Dus het is gedaan? **Ja.** |
| DEEL 2 AANPASSEN ACTIEPLAN |  |  |
| DEEL 2 REST |  |  |
