# Supplementary material for: Users’ thoughts and opinions about a self-regulation-based eHealth intervention targeting physical activity and the intake of fruit and vegetables: A qualitative study
Source: PLoS One. 2017 Dec 21;12(12):e0190020. doi: 10.1371/journal.pone.0190020 (PMC5739439; doi:10.1371/journal.pone.0190020)
Supplement: S3 File — This file contains the transcribed interviews. (ZIP) [file pone.0190020.s003.zip › general_population/TA1BEYE.docx]

**Code filmpjes:**

| Deel interventie | Minuten | Transcript |
| --- | --- | --- |
| DEEL 1  VRAGENLIJST | 0-10 | **Je mag alles zeggen wat er in je opkomt. Als dat kan helpen mag je ook luidop lezen.** .. ik lees traag sorry.  Moet je ze alle drie doen? **Neen je mag eentje doen**. Ik ga groenten pakken. **Je mag echt luidop denken.** Ik heb maar 1 dag groenten gegeten. 1 keer is 1 dag he? **Ja.** ik ben echt erg op vlak van groenten.  …….  **Je mag zeggen wat er in je opkomt.**  …..  ik eet maar 120 gram groenten en ik heb dat maar 1 keer in de week gegeten.  **En wat denk je bij die vragen?**  Als je een pak sla koopt dat wordt slecht he. |
| DEEL 1 ADVIES |  | *Niets gezegd* |
| DEEL 1 OPSTELLEN ACTIEPLAN | 10:45 – 16:23 | Werk is dan school he? **Ja.** is dat normaal dat er daar nul blijft staan vanboven? **Ja. dat is het blauw dat betekent hoe ver dat we zitten.**  **…..**  ik vind dat moeilijk, ik weet dat nooit.  Oei waar, ik ga gewoon thuis nemen en dat is dan hier.  ….  Hoe zeg je dat een hongertje. Dan eet ik een wortel.  **Je moet vandaag ingeven anders blokkeert het systeem.**  Ik ben daar echt slecht in.  Met computers kan ik niet werken.  Neen hoeft niet, niemand moet dat weten.  **Je moet op versturen klikken.** |
| DEEL 1 ACTIEPLAN |  | *Niets gezegd* |
| DEEL 2 VRAGENLIJST |  | **Je mag opnieuw alles zeggen wat in je opkomt.** Er komt niet zoveel op in mij.  Ik zal 2 dagen zeggen zeker.  Tomaten eet ik sowieso niet.  Eetlepel komkommer, hoe kan je dat nu eten?  **Waarom lach je?**  Omdat ik hetzelfde doel ga nastreven.  **Je mag opnieuw versturen.** |
| DEEL 3 |  | Hetzelfde als daarnet. Ik lust geen tomaten.  Ik twijfel, maar ik ga gedeeltelijk doen. Dat zal wel geslaagd zijn.  Oke dat was alles. **Ik heb juist nog een paar vragen. Wat kwam er van positieve of negatieve elemente in je op?** Ik vond het goed dat je geconfronteerd wordt met het feit dat je zo weinig groenten eet.  **En iets negatief?** Veel dezelfde vragen. |
