# Supplementary material for: Users’ thoughts and opinions about a self-regulation-based eHealth intervention targeting physical activity and the intake of fruit and vegetables: A qualitative study
Source: PLoS One. 2017 Dec 21;12(12):e0190020. doi: 10.1371/journal.pone.0190020 (PMC5739439; doi:10.1371/journal.pone.0190020)
Supplement: S3 File — This file contains the transcribed interviews. (ZIP) [file pone.0190020.s003.zip › general_population/TA1BIAN.docx]

**Code filmpjes:**

| Deel interventie | Minuten | Transcript |
| --- | --- | --- |
| DEEL 1  VRAGENLIJST | 0-7:30 | Ik ga fruit nemen. Hoe heet je.. maakt dat uit. Dat is om het wat persoonlijker te maken. Ik vind het raar dat ze dat willen. Ze zeggen dat het anoniem is en de eerste vraag is ‘hoe heet je’ dat is toch wel heel raar. Wat is je email adres.. als ze mij daar niet mee gaan stalken .. ze gaan mij een reminder sturen .. ah oke. Ik dacht iedere keer als ik iets niet deed in dat actieplan. Hoeveel heb ik fruit gegeten.. oei oei dat zal niet veel zijn. een schaaltje fruitsla. Moet je iedere keer als je dat niet gedaan hebt neen invullen? Awel dat vind ik lichtelijk ambetant. Als dat een slim programma zou zijn zet je automatisch nul.dan heb ik liever een open vraag zodat je kan invullen wat je gegeten hebt. Als ik meer fruit eet dan is mijn kans op ziekten pfffffff.. juist. Zullen anderen mijn steunen?? Het is nu echt niet dat ik een fanclub ga ontwikkelen, de meeste mensen gaan zeggen goed voor u. Ja ik ben er zeker van dat ik kàn ja natuurlijk juist. Je hebt kunnen en kunnen he. Maar of ik het realistisch vind, dat is iets anders. Problemen en zorgen maar seg, dat heeft daar niets mee te maken. Ik vind het overdreven. ik heb het moeite om doelen te stellen, waarschijnlijk wel. Plannen te maken, waarschijnlijk wel. Ik hou mijn voortgang bij, neen. Ik heb een duidelijk plan .. wie heeft er dat gemaakt die vragenlijsten … **iemand van de universiteit.** Ik heb in mijn leven zodanig veel plannen over vanalles en nog wat, maar niet over fruit. Mega grappig. Alleja. Oneens hé ja. dat hangt er ook van af hoe belangrijk je dat vindt he. Ik heb een plan voor als er iets tussen mijn plan komt. Maar jongens toch. |
| DEEL 1 ADVIES | 7:30-8:00 | Eric en eline, maar seg. Haha! Eric koos.. hahah dat vind ik verschrikkelijk kinderachtig, ik vind het gewoon hilarisch. Het is precies voor in de lagere school seg! Het is precies een vraagstuk. Ik ga dat nu doen omdat ik echt wel wil zien wat die mij gaan aanraden. |
| DEEL 1 OPSTELLEN ACTIEPLAN | 8:00 – 13:00 | Het is wel duidelijk he. Maar ‘denk je dat je het in bepaalde situaties moeilijk zal vinden’ ze hebben dat nog maar net gevraagd en ik heb ja gezegd, en dan herhalen ze gewoon weer die vraag daar kan ik echt niet tegen ik doe niet graag 2x hetzelfde. Als je een programma maakt moet je zorgen dat dat doorloopt, dat ze dat onthouden. Dat kan toch niet zo moeilijk zijn om dat te programmeren. Aangezien het een persoonlijk actieplan is zouden ze moeten schrijven ‘u gaf aan…’ dan heb je zoiets van ze hebben het onthouden, dat is positief. En ze hebben dat daarnet ook al gevraagd. Ik heb dat daarnet ook ingevuld. Welke opties kunnen hindernissen vormen.. maar allez.. ik heb ik geen steun nodig van mijn vrienden en familie. Ik bereid fruit niet graag, ja ik ben lui. Ik maak geen tijd. Ik vergeet het. Ahja. Vooral da. Welke optie is de belangrijkste hindernis. Ik vergeet het. ‘hoe wil je proberen’ ze hebben nog niet eens gevraagd of ik wil proberen. Aha nu ben ik curieus. Door op vaste momenten te eten .. t schijnt dat je dat niet mag eten bij je maaltijd? Dat dat super slecht is omdat dat gist in je maag? Pascale naessens en nog veel anderen zeggen dat. Ik ken er niets van maar ik heb het al heel dikwijls gehoord. Als dat waar is dan is dat wel héél erg dat dat op een site staat van gezonde voeding he. **Ik weet er ook het fijne niet van**. Dat staat overal in alle boeken. Klaar leggen zal bij mij al helpen. Hoe wil je beginnen.. meer dagen ja allez dan. Hoeveel porties, jong, 1 zal al goed zijn he. 7 op 7. Waar wil je fruit eten.. wat voor vraag is dat nu weer. Op mijn werk. Wanneer. Volgende. Als-dan: haha. Oei oei zeg dan moet ik dat nog invullen ook. Als ik op mijn werk fruit wil eten, dan ga ik naar de mini delhaize om er te kopen. Vanaf wanneer. **Je moet vandaag ingeven anders blokkeert het systeem**. gelukkig dat je dat zegt. |
| DEEL 1 ACTIEPLAN | 13:00 | Ik ga dat niet doen. Voila. Maar seg. Pas op dat kan nuttig zijn he, maar niet voor fruit. Voor mij hé. Versturen. |
| DEEL 2 VRAGENLIJST | (2^e^ fragment) | Dat is toch niet normaal dat ze dat niet meer weten? Maar kom zeg .. Dat vind ik echt nie oke. Ze sturen u een mail naar aanleiding van u actieplan en ze vragen of je er een hebt gemaakt, het is precies een vragenlijst rond Alzheimer. Jouw doel .. als-dan als-dan, .. **hier moet je doen alsof.** O my god zeg .. serieus. Dat vind ik echt niet wijs. En zo een beue dingskes. En zo een eindeloos lange rij dat is om depressief van te worden. zo beu. Kunnen ze dat niet doen met bolletjes zoals daarnet, daarnet had je zo een goed overzicht en je moest gewoon een kruisje zetten, nu moet ik hier elke keer die vakjes invullen. En zo dicht bij elkaar.  Mijn doel was te moeilijk voor mij.. neen ik was onvoldoende gemotiveerd. Hatelijk. Ze zeggen ‘misschien kan het je helpen om nog dingen te lezen’ maar als je daar geen zin in hebt, dan moeten ze nie vragen of het ‘misschien’ is he, dan moeten ze eerst vragen ‘wil je dat’ ja of nee, en als het ‘neen’ is naar de volgende vraag. Tips om meer fruit te eten. Allez nu krijg ik dat wéér. Ik zal maar eens proberen anders krijg ik hier weer iets nieuw. Ik vind het ook totaal geen attractieve website.. je moet eens kijken. Dat is zo lelijk gelijk de nacht, dat is niet aantrekkelijk dat is niet interactief dat is niets, dat is precies van in de jaren .. dat is voor oude mensen. En dan die layout alleen al. Dat is echt niet oké.  Aha een open vraag! Ze gaan er precies toch aan beginnen. |
