# Supplementary material for: Users’ thoughts and opinions about a self-regulation-based eHealth intervention targeting physical activity and the intake of fruit and vegetables: A qualitative study
Source: PLoS One. 2017 Dec 21;12(12):e0190020. doi: 10.1371/journal.pone.0190020 (PMC5739439; doi:10.1371/journal.pone.0190020)
Supplement: S3 File — This file contains the transcribed interviews. (ZIP) [file pone.0190020.s003.zip › general_population/TA1BIBI.docx]

**Code filmpjes:**

| Deel interventie | Minuten | Transcript |
| --- | --- | --- |
| DEEL 1  VRAGENLIJST | 0-13:50 | De inleidende tekst geeft je wel het gevoel dat je het wilt lezen. Als je het leest wil je ook wel doorgaan. … Het blijft wel uitnodigend.  *(vult gegevens in).*  Het gaat wel vlot he. Ik vind wel als je aan het lezen bent dat je niet direct zo iets luidop kunt gaan denken of zeggen omdat je zodanig aan het lezen bent..  ….  Het is eindelijk met momenten he.. het hangt af van hoe druk je het hebt en of er iemand is om mee te gaan..  Dat is in het algemeen he? Werk vrije tijd alles he**? Ja.**  Dat zal wel juist zijn zeker .. ik weet niet of het te maken heeft met bewegen. Ik denk niet dat dat er iets mee te maken heeft. Mentaal beter voelen ja. Doelen heb ik maar ze uitvoeren is iets anders. |
| DEEL 1 ADVIES | 13:50-15:35 | Dat is eigenlijk wel leuk om te weten he zo. We gaan dat misschien niet altijd opzoeken of naartoe gaan ofzo maar eigenlijk. Nu moet ik aanklikken of ik dat wil of niet? **Ja, voor het onderzoek is het eigenlijk belangrijk dat je zou zeggen van ‘ja’. Tenzij je zegt ik heb nu gezien dat ik genoeg beweeg en ik wil liever fruit of groenten kiezen, ik zal een andere module doen. Maar dan krijg je weer vragen daarover**. Wat ik wel vind is als je een actieplan zou opstellen denk ik dat je er je rapper gaat aan houden. Als je echt een plan hebt denk ik dat je zoiets hebt van – anders zou je er ook niet aan beginnen he. Dus ik ga het wel doen. |
| DEEL 1 OPSTELLEN ACTIEPLAN | 15:35 – 23:35 | Gebrek aan tijd is de belangrijkste reden he eigenlijk.  …  Snowboarden.. maarja je moet iemand vinden dat het kan. Er zijn niet veel mensen..  Waar.. al de rest weet ik eigenlijk niet echt. Als je het voor jezelf weet is het oké. **Het maakt niet veel uit wat je precies invult, dat is voor jezelf eigenlijk.**  Als-dan: mijn zinsbouw is eigenlijk bijna hetzelfde he. Ik ga squash er ook bij zetten. **Hier mag je gewoon de datum van vandaag invullen. Ja.** |
| DEEL 1 ACTIEPLAN | 23:35 – 25:11 | Dus dat moet je dan eigenlijk printen. Versturen. **Als je dat wil mag je dat doen, maar het hoeft niet.** Bijhouden in mijn agenda, dat zal het makkelijkste zijn. |
| DEEL 2 VRAGENLIJST | (2^e^ fragment)  0 – 7:52 | Dat heeft te maken met dat actieplan he? **Ja je moet doen alsof je een week verder bent.** **Het is vooral om te kijken van want vind je daarvan, wat er dan in je opkomt.** Wandelen is echt mijn ding niet.  …  Op het werk ben je altijd meer bezig hé.  …  Versturen? **Ja**. |
