# Supplementary material for: Users’ thoughts and opinions about a self-regulation-based eHealth intervention targeting physical activity and the intake of fruit and vegetables: A qualitative study
Source: PLoS One. 2017 Dec 21;12(12):e0190020. doi: 10.1371/journal.pone.0190020 (PMC5739439; doi:10.1371/journal.pone.0190020)
Supplement: S3 File — This file contains the transcribed interviews. (ZIP) [file pone.0190020.s003.zip › general_population/TA1BICE.docx]

**Code filmpjes:**

| Deel interventie | Minuten | Transcript |
| --- | --- | --- |
| DEEL 1  VRAGENLIJST | 0-10 | Ik probeer iedere dag toch een stuk fruit te eten maar dat wordt soms eens overgeslaan. Het kan beter. Ik denk dat ik genoeg beweeg in combinatie met mijn dansen omdat ik 2x per week ga dansen. En als ik ga werken is het de bedoeling dat ik in het weekend ook loop. Maar vanmorgen dus niet met de paasvakantie, het is een nieuwe maand, het is goed weer dus ik had geen excuus. Ik denk dat dat voor mij genoeg is. Maar het kan beter he! Ik weet niet of ik het kan opbrengen voor meer .. groenten, zeker voldoende. ik ben een rauwkost freak. 's middags altijd worteltjes en dan ’s avonds bij mijn warm eten ook altijd groenten. Wil ik meer bewegen? Hmm ik vrees dat het er niet van zou komen. Gezonder eten, eigenlijk wel. Ik denk dat ik mijn best doe maar het kan zeker wel beter. (vult gegevens in) 300g per dag, daar kom ik zeker wel aan. 2 porties fruit neen, het is meestal maar 1 portie, maar als er druiven en aardbeien in huis zijn .. dan meer. **Nu gaan we ons focussen op het huidige.** 30 minuten bewegen, als je je huishouden daarbij rekent.. maar apart kom ik daar niet aan. Ik ga fruit nemen.  **Je mag constant zeggen wat in je opkomt**. Positief is dat het een groot lettertype is. en dat er ook veel oudere mensen in te vinden is dat is zeer positief. *(invullen gegevens)* |
|  | 10-23 | Het was geen goede week vorige week. Het is wel chocolade week he! Ik eet meestal wel een yoghurtje met fruit in maar dat telt niet he? **Neen. Fruitsap ook niet.** Ik eet wel fruit op mijn werk omdat ik een portie mee neem, maar dat is er wel maar 1. Oei en het is allemaal van de voorbije week .. *(vult soorten fruit in)* hoeveel fruit denk je dat je eet.. niet veel niet weinig. **Als er negatieve zaken zijn mag je het altijd zeggen.** Tot nu toe vind ik het wel goed. *(vult vragenlijst verder in)* |
| DEEL 1 ADVIES | 23-26 | Ja meer dagen oké. Wil je een actieplan, ja he! Als je wilt meewerken moet je het goed doen. **Wat vond je van dat onderdeel?** Ik vond de vragen wel.. **neen ik bedoel de tekst van nu net.** Ja ik vind dat wel goed dat je ermee geconfronteerd wordt en eens beseft, je werd er ervoor ook door de vragen mee geconfronteerd maar.. |
| DEEL 1 OPSTELLEN ACTIEPLAN | 26 - 35 | *(vult vragen in)* gebrek aan steun .. ze ontmoedigen ook niet he, ze doen gewoon niets dus niet van toepassing. Wat bedoelen ze met ‘ik weet niet wanneer ik fruit kan eten’? te weinig op mijn werk .. ja. Zijn dat dezelfde dingen weer van daarnet? **Ja maar nu is het de belangrijkste reden.** Dat komt hier wel allemaal heel duidelijk over!  Vaste momenten, dat is op mijn werk. Wat bedoelen ze daarmee? **In de plaats van een klein stukje een groot stuk.** bij familie en vrienden ga ik daar niet gaan vragen van ‘mag ik wat fruit’ of op restaurant .. daar eet ik wel wat anders.  ‘als-dan’: maar ja ik eet niet elke avond chips he! **Maar dat zijn voorbeeldjes.** Als in de voormiddag iets wil eten, dan eet ik een stuk fruit**. Als je wilt mag je er nog maken**. Als ik een dessert wil eten, dan kies ik ook voor fruit. **Ja heel goed. Je moet de datum van vandaag aanduiden anders klopt het programma niet.** |
| DEEL 1 ACTIEPLAN | 43:00 | *(leest actieplan voor)* **wat vond je van dat actieplan?** Dat vond ik leuk want je kon het nog wijzigen als je wilt! Hoeveel kan je eigenlijk wijzigen? **Je kan helemaal terug gaan** oh oke! |
| DEEL 2 VRAGENLIJST | (2^e^ fragment) | Heb je de vorige keer een actieplan gemaakt, ja. Waarom is dat, heb ik dat gezegd? **Neen ik weet niet waarom dat in fluo staat!** Ik ga nu wel 6 dagen kiezen. Ook de zaterdag.  Dat is wel iets dat heel dikwijls vergeten wordt dat je nul moet ingeven, misschien is het een verbetering als dat standaard op nul staat? **Ja dat zou goed zijn.**  Ik ben er op vooruit gegaan he! Er is wel nog niet zoo veel omhoog he? Maar ahja ja de dagen zijn verdubbeld! Ik was enkel naar de porties aan het kijken.  Je bent ‘niet’ in je doel geslaagd, maar je bent er wel op vooruit gegaan.. maar ik heb toch meer dagen? Ik vind dat dat een beetje negatief klinkt. Voor mij komt dat negatief over. Ik ben op de goede weg, maar omdat die ‘niet’ in vetjes staat wordt daar je aandacht op gevestigd .. daar staat ‘meer fruit’ in vet dat is wel goed. Dat springt toch in het oog he wat in het vet staat.  Ik ga zetten dat mijn doel te hoog was he. |
| DEEL 2 AANPASSEN ACTIEPLAN | 7:40 | Maar ik ga het niet veranderen, blijven volhouden. |
| DEEL 3 REST | (3^e^ fragment) | Ik denk niet dat iemand dat na de derde keer nog allemaal leest.. ik denk dat als het samengevat zou zijn dat het beter zou zijn.  Nu geeft die maar een ‘als dan’ ..  *(vult lijst met fruit opnieuw in)*  **Voila dat was het einde! Ik heb nu wel nog een paar kleine vraagjes. Welke zaken vond je echt goed daarin?** Dat het een groot lettertype is. het was goed dat ik alles kon lezen en dat het duidelijk was, die kleuren vond ik positief. Alhoewel dat ik soms moest kijken zit ik nog bij de juiste vraag, het had misschien beter opzij gekomen met die porties.  **Welke zaken vond je dan slecht?** Daar in het begin, al die tekst voor de algemene gegevens, je loopt daar zo rap door en je leest dat niet echt, zeker bij de tweede enquête. **En zijn er punten ter verbetering die jij voorstelt?** Ja nee ik vind het eigenlijk wel goed! |
