# Supplementary material for: Users’ thoughts and opinions about a self-regulation-based eHealth intervention targeting physical activity and the intake of fruit and vegetables: A qualitative study
Source: PLoS One. 2017 Dec 21;12(12):e0190020. doi: 10.1371/journal.pone.0190020 (PMC5739439; doi:10.1371/journal.pone.0190020)
Supplement: S3 File — This file contains the transcribed interviews. (ZIP) [file pone.0190020.s003.zip › general_population/TA1BICK.docx]

**Code filmpjes:**

| Deel interventie | Minuten | Transcript |
| --- | --- | --- |
| DEEL 1  VRAGENLIJST | 0-9:45 | *(vult persoonlijke gegevens in).* Beweging doe ik al, ik ga groenten nemen. Fruit ik ook niet zoveel maar ik ben er een beetje allergisch aan. Heb je al een andere module ingevuld, neen.. *(vult verder persoonlijke gegevens in)* wat is je leeftijd, weeral..  op hoeveel dagen heb je de voorbije dagen groenten gegeten, pak maar 4. Hoeveel heb je gegeten van deze soorten. Sla heb ik veel gegeten. 3 zeker he. Rauwe tomaten 1 keer. Schijfjes tomaat. Eetlepels geraspte wortelen. Volledig rauwe ook niet. Komkommer niet.  Hoeveel groenten denk je dat je eet.. niet zo veel.  Ben je van plan meer groenten te gaan eten, waarschijnlijk niet. Hoe moeilijk is het, hmm waarschijnlijk wel gemakkelijker want het wordt zomer en dan eet je makkerlijker slaatjes.  Dan is mijn kans op ziektes kleiner, ik hoop het maar ik denk dat dat daar weinig mee te maken heeft. Zal ik mij mentaal beter voelen, dat denk ik ook niet. En zullen anderen mij steunen, dat zal zeker niet zo zijn.  Meer groenten kan eten ook in moeilijke situaties, ja dat kan.  Meer groenten kan eten ook als ik problemen of zorgen heb, neen ik heb geen zorgen. Meer als mijn familie of vrienden dat niet doen, nee, volgende. Ik heb een duidelijk plan: oneens. Hoeveel, dat weet ik ook niet. Als er iets tussen mijn plannen komt: dat zijn heel moeilijke vragen om te antwoorden hoor. Ik heb er moeite mee om doelen te stellen, waarschijnlijk niet. Ik heb er moeite mee om plannen te maken die mij helpen om mijn doelen te bereiken: dat is een moeilijke vraag hoor. Ik hou mijn voortgang bij, ah ja dat is waarschijnlijk wel zo. Volgende. |
| DEEL 1 ADVIES | 9:45-10:53 | *(leest advies voor).* 300g per dag, oei oei ! wil je graag een actieplan maken, ja. volgende. |
| DEEL 1 OPSTELLEN ACTIEPLAN | 10:53 – 20:00 | Ik denk dat ik soms moeilijke situaties zal ondervinden. Volgende. Belangrijkste hindernissen. …..  Bij uitnodiging om te gaan eten zijn er niet altijd groenten.. er is niet altijd een menu met groenten voorzien.  Door groenten op mijn boterham te eten … een extra schaaltje groenten bij de broodmaaltijd dat vind ik wel goed. Voldoende groenten bij de warme maaltijd. Groenten als aperitiefhapje, salades .. diepvriesgroenten ..  …  Ik ga 7 dagen kiezen. 300g lijkt mij heel veel precies, ik ga beginnen met 150g. Dat zal al een goede start zijn. waar vooral: thuis en op het werk.  Op welke momenten: niet bij het ontbijt, tussendoortje niet. Wel tijdens het middagmaal. Tijdens het avondmaal. Dat zal het zijn.  ‘als – dan’.  **Je mag de datum van vandaag invullen.** |
| DEEL 1 ACTIEPLAN | 20:00 – 23:20 | Wauw! Ik hoop dat dat lukt! Ja. salade op restaurant. Vanaf morgen dus! Dat lijkt mij goed! Volgende.  Duid de methode aan die je wilt volgen om je doel bij te houden, ik ga dat doen in mijn agenda op mijn gsm. Een combinatie eigenlijk. Een app neen, dat is niet aan mij besteed. Je kan maar een ding aanduiden precies? Ik zal dan dat pakken.  Wil je het actieplan aan iemand versturen.. ja. ik ga mijn dochter invullen, die zal dat wel positief vinden. **Als je het echt graag wilt kan ik het opvragen en naar jou doorsturen**. Ja dat is goed.  Tijd voor actie. Het is wel een interessante actie! |
| DEEL 1 REST |  |  |
| DEEL 2 VRAGENLIJST | *2^e^ fragment*  *3^e^ fragment* | **Dit is de mail die je na een mail krijgt. Dus nu kan je terug starten.**  *(vult vragen verder in)*  Vroeger had je op 4 dagen groenten .. je doel was 150g, je geeft aan dat je 5 dagen groenten hebt gegeten, 650 gram amai! Je bent gedeeltelijk geslaagd. Nog niet in het aantal dagen geslaagd. Het aantal dagen is wel al gestegen. Je bent goed begonnen.  Ik kon mijn doel niet behalen door omstandigheden.. volgende. Ik werd uitgenodigd bij vrienden en er was geen mogelijkheid om groenten te eten. Oei, dat is nu terug diezelfde vraag? Het is twee keer hetzelfde he.. de reden is terug dat ik bij vrienden ben uitgenodigd en daar geen groenten waren, dus ik ga dat dan terug invullen.  Hoe wil je proberen .. salades ja dat is goed. Diepvriesgroenten dat gaat ook. Middagmaal en avondmaal, de andere dingen vind ik niet zo interessant. Thuis en op het werk. Als-dan plan. Dat is terug hetzelfde als de vorige keer he. Als ik boodschappen doe, dan zal ik meer groenten meebrengen.  Dit is jou nieuw actieplan. Ben je tevreden – volgende week kan je terug evalueren.. je kan ons programma nu sluiten. |
| DEEL 2 AANPASSEN ACTIEPLAN |  |  |
| DEEL 2 REST |  |  |
