# Supplementary material for: Users’ thoughts and opinions about a self-regulation-based eHealth intervention targeting physical activity and the intake of fruit and vegetables: A qualitative study
Source: PLoS One. 2017 Dec 21;12(12):e0190020. doi: 10.1371/journal.pone.0190020 (PMC5739439; doi:10.1371/journal.pone.0190020)
Supplement: S3 File — This file contains the transcribed interviews. (ZIP) [file pone.0190020.s003.zip › type_2_diabetes/TA2BODA.docx]

**Code filmpjes:**

| Deel interventie | Minuten | Transcript |
| --- | --- | --- |
| DEEL 1  VRAGENLIJST | 0-10 | *(vult gegevens in)* 300g groenten per dag, dagelijks 30 minuten bewegen … ik eet zeker geen 300g groenten per dag, 2 porties fruit dat zal in de omgeving zijn, 30 minuten bewegen, ik doe enkel de trap en voor de rest als ik ergens naartoe ga is het met de auto. Ik ga soms 150 meter, 250 meter – waar ik moet zijn he. Dus ik ga groenten kiezen. Hoe heet je .. nog eens mijn naam? *(vult gegevens in)* volg je een speciaal dieet, ik heb nog een andere aandoening waarvoor ik medicatie neem. **In dat geval mag je ‘nee’ aanduiden, dat is oké.**  … |
|  | 10-15:50 | **Je mag blijven luidop zeggen wat er in je opkomt**. Dat ik te weinig groenten eet he ja .. **ja en ook hoe u de website ervaart.** Ben je van plan om meer groenten te gaan eten, waarschijnlijk wel. Want het wordt zomer, dan eet ik wel groenten. Zal ik mij mentaal beter voelen.. waarschijnlijk wel. Zullen anderen mij steunen.. ja. Meer groenten kan eten dan ik nu doe, ja dat is zo. In moeilijke situaties.. dat is moeilijker hé. Als er minder groenten beschikbaar zijn ga ik waarschijnlijk ook minder groenten eten. Ook als ik telkens opnieuw moet proberen tot het me lukt.. dat is niet makkelijk hoor dat.  Neen ik heb nog geen plannen. **Het is de bedoeling dat u ‘ja’ aanklikt zodat we kunnen verder gaan.** |
| DEEL 1 ADVIES | 15:50-18:00 | **Je mag echt zeggen wat je denkt enzo hé bij de website**. Ik vind dat het begrijpbaar is wat er staat, en het is wel lang uitgesponnen..  Dat is interessant. |
| DEEL 1 OPSTELLEN ACTIEPLAN | 18:00 – 24:00 | **Ik zag je daarnet lachen, waarom was dat?** Hier staat er: Ik heb te weinig tijd om groenten te bereiden en erboven dat is, dat is voor mij ja – ik ben alleen he. Kan ik dat hier aanduiden?  Meerdere antwoorden mogelijk? Ja he. Eens diepvries, .. blikgroenten daar ben ik niet voor. Wil je groenten eten… per week.. nja. Ik denk 200g.  Als-dan .. **je mag zeggen als je iets vreemd geformuleerd vindt**. Dat vind ik hier.. die dan – als of als dan .. voor mij gaat dat zijn ’s middags he.. **hier is het belangrijk dat je de dag vandaag ingeeft.** |
| DEEL 1 ACTIEPLAN | 24:00 - 32 | Ja.. BMI is 28,7, ik had meestal 30. Maar ik ben een paar kilo afgevallen. Het is nog teveel he. Ik weet dat dat heel belangrijk is. ’s Avonds ook he.. mijn vrouw zegt eet een stuk fruit maar ik kan het niet laten van koeken te eten. Dat is typisch aan diabetes dat je die suiker wilt hebben he. Een tussendoortje, een yoghurt hé. Dat is voor mij nog een oplossing. Soep ik ook nog goed, die ik zelf maak, en dat is zonder vet dan he. Er wordt geen zout in gedaan, en dat is ook wel lekker dat. Het feit dat je dat schrijft is belangrijk he.. in mijn agenda. Dat is wel interessant.  …  Als ik dat nu verstuur naar iemand.. ik ga eens proberen hé. |
| DEEL 2 VRAGENLIJST | *(2^e^ fragment)*  0-10 | Het is dus de bedoeling dat ik antwoord na een week he? **Ja.** Ik ga dan fictief invullen he. Dus 3 dagen. Sla heb ik niet gegeten. Schijfjes tomaat, is dat hoeveel schijfjes? Euhm ..  Ah oei er is nog een niet ingevuld. .. en nog één niet ingevuld! Hoe komt dat .. andere gekookte groenten .. witloof, spruiten .. dat is er duidelijk onder hé, hoho ! **je mag terug luidop zeggen, het is moeilijk he**. Het is duidelijk he. Deze 2 zijn de enige mogelijkheid he.  Het gaat hier zijn of hier maar niet veel anders ..  Thuis .. ja .. het is weer dan en als he. Tja. |
|  | 10-16 | **Opnieuw is het hier de bedoeling dat je de datum van vandaag kiest**. Ahja. Wat is er nu weeral verkeerd .. ah dat is nog niet ingevuld. Door welke omstandigheden kon je jouw doel niet bereiken .. jah, wat is het doel? **Je mag anders gewoon een fictief iets zetten.** Waarom ik het niet doe, dat is hier weg he.. ik ga zetten...  En nu de volgende.. **eigenlijk heeft u dat al ingevuld.** Het is hetzelfde als de vorige keer he. **Dat is doordat u iets nieuws heeft aangeduid.** Next. Versturen? **Ja. Oké!** |
| DEEL 3 | *(3^e^ fragment)*  0-5:44 | **U mag opnieuw dingen zeggen dat u denkt..** ik had 4 he de vorige keer? Ik ga zeggen 4 dagen.  1 rauwe wortel weegt gemiddeld 100 gram, is dat dan het aantal keer dat je dat eet per week? **Dat mag ik niet zegen maar dat is goed dat je dat zegt dat dat een onduidelijkheid is in de website.** Dat zijn dat vragen dat ik mij stel.. is dat dan hoeveel gram dat ik gegeten heb of hoeveel keren dat ik het gegeten heb die 100g? 2.. is dat dan 200g? ik weet het niet. |
