# Supplementary material for: Users’ thoughts and opinions about a self-regulation-based eHealth intervention targeting physical activity and the intake of fruit and vegetables: A qualitative study
Source: PLoS One. 2017 Dec 21;12(12):e0190020. doi: 10.1371/journal.pone.0190020 (PMC5739439; doi:10.1371/journal.pone.0190020)
Supplement: S3 File — This file contains the transcribed interviews. (ZIP) [file pone.0190020.s003.zip › type_2_diabetes/TA2BODE.docx]

**Code filmpjes:**

| Deel interventie | Minuten | Transcript |
| --- | --- | --- |
| DEEL 1  VRAGENLIJST | 0-10 | Eet u voldoende fruit per dag.. wat is voldoende? Ik probeer na het eetmaal een peer of appelsien te eten. Soms een appel ook. **Je moet doen alsof je hier alleen bent, ik ben er niet.**  *(vult gegevens in)* die kleine lettertjes zie ik niet goed.  Dessertschaaltjes, nul. Is dat de voorbije week? Neen he, nul. Nectarines nul. Mango’s. nul.  Eetlepels fruitmoes, nul. **Je mag luidop zeggen wat je erbij denkt**. Is mijn kans op het ontwikkelen van ziektes kleiner.. dat zal wel juist zijn. zal ik mij mentaal beter voelen, dat weet ik niet ze.. dat zal wel juist zijn. Zullen anderen mij steunen.. ik ben er zeker van dat ik meer fruit kan eten, ja. Kunnen ja. |
|  | 10-12 | Moeilijke omstandigheden.. telkens opnieuw tot het me lukt .. ja. Ook als ik problemen en zorgen heb.. moet je dan meer fruit eten? Dat heeft er niets mee te maken. Ook als mijn familie en vrienden dat niet doen, ahja he. Ik heb er moeite mee voor mezelf doelen te stellen.. waarschijnlijk niet. Ik heb er moeite mee plannen te maken, neen. Ik hou mijn voortgang bij.. voortgang? Baneen. Ik heb een duidelijk plan voor wanneer ik meer fruit ga eten.. ik heb geen plan hé. Neeje. Hoeveel, dat is toch weer hetzelfde? Ah hoeveel! Neje, geen plannen. Op dat punt heb ik niet teveel plannen ze. |
| DEEL 1 ADVIES | 12:30-13:00 | *(leest advies voor)* **het is de bedoeling dat je op ja klikt, en je mag ook altijd zeggen wat je van het advies vindt enzo.** |
| DEEL 1 OPSTELLEN ACTIEPLAN | 13:00 – 19:00 | Dat zou kunnen, fruit als tussendoortje te eten. Nieuwe soorten, ja dat hangt er van af.. druiven raden ze ons af als diabetespatiënt.. fruit bij de warme maaltijd dat doen we he, dan moet ik het niet aanstippen he? Fruit als aperitiefhapje, dat zie ik niet zitten. Fruit bij salades.. we hebben een schaal dus feitelijk .. ik denk dat dat alles is. het is weinig he. geeft het niet ? **neeneen het is goed!**  ….  Grotere porties .. twee porties! Waar.. thuis. Als tussendoortje in de voormiddag of namiddag. ’s Avonds is dat moeilijk.  Als-dan: …  Vanaf wanneer. **Je moet de datum van vandaag instellen en dan mag je op volgende klikken**. Ja. |
| DEEL 1 ACTIEPLAN | 19:00 – 25:00 | **Wat vindt u van het actieplan?** Dat is een beetje naar mijn gevoel, kunstmatig. Met fruit dat ik nu eet – mijn vrouw houdt in haar keuken heel goed rekening met mijn diabetes. En ik voel met de manier waarop ik nu leef en fruit gebruik voel ik mij goed. Ik heb geen behoefte om meer te doen. Als je zegt van ‘je moet’ dan ga ik het doen, maar vanuit mijzelf vind ik het niet noodzakelijk om te doen. Alles is goed geregeld, suiker zit goed. De laatste jaren heb ik geen probleem, dus dat systeem vind ik goed dat ik nu doe. Ik heb niet de gewoonte van de voormiddag én de namiddag, ik zit dikwijls in vergadering ik ben zeer actief, de omstandigheden laten het niet toe. En als je om 18:15 eet ga je om 17:00 geen fruit meer gaan eten. Dat is zo mijn reactie op heel dat plan. **Ja oke!** 1 zal genoeg zijn! Volgende. Tijd voor actie. Oke. |
