# Supplementary material for: Users’ thoughts and opinions about a self-regulation-based eHealth intervention targeting physical activity and the intake of fruit and vegetables: A qualitative study
Source: PLoS One. 2017 Dec 21;12(12):e0190020. doi: 10.1371/journal.pone.0190020 (PMC5739439; doi:10.1371/journal.pone.0190020)
Supplement: S3 File — This file contains the transcribed interviews. (ZIP) [file pone.0190020.s003.zip › type_2_diabetes/TA2BODI.docx]

**Code filmpjes:**

| Deel interventie | Minuten | Transcript |
| --- | --- | --- |
| DEEL 1  VRAGENLIJST | 0-9:50 | Het is een appartement dus ik heb geen tuin he. Zware fysieke activiteit.. geen. Matige fysieke activiteit.. neen. Ik geloof dat ik een vraag mis begrepen heb: er staat hier hoeveel dagen in de gewone week wandel je om ergens naartoe te gaan, dit heb je al vermeld die vraag.. **normaal gezien kan je op vorige klikken en dat aanpassen.** Oke. Dit zal het zijn hé. Ik ben nogal passief op dat gebied hoor. **Dat is geen probleem.** Matige fysieke activiteit .. geen. Geen. Dat is wel wat dubbelzinnig he, verplaatsing van en naar je werk hoort hier niets tegen, is dat dan ook vrijwilligerswerk of wat? **Ja het staat erbij he: betaald werk, vrijwilligers werk.** Ja ja maar, ‘van en naar je werk hoort er niet bij’ dus je moet dat nog meer specifiëren. Ben je van plan meer te bewegen de komende maand, zeker wel. Ik heb mezelf beloftes gedaan. Als ik meer beweeg dan is mijn kans op het ontwikkelen van ziektes.. dat is juist. Zal ik mij mentaal beter voelen.. joah. Ik ben er zeker van dat ik meer kan bewegen dan ik nu doe, juist. Ook in moeilijke situaties, ja. Ook als ik telkens opnieuw moet proberen .. fout. Juist. **Zou ik de radio misschien ietsje stiller mogen zetten ik ben bang dat ik anders je niet goed ga kunnen horen.** Ja natuurlijk. Voila! Ik ben er zeker van dat ik meer kan bewegen.. ik heb er moeite mee om doelen te stellen: zeker niet. Ik heb er moeite mee om plannen te maken.. zeker niet. Ik hou mijn voortgang bij: waarschijnlijk.. misschien wel misschien niet. Ik heb een duidelijk plan voor wanneer ik meer ga bewegen. Mee eens. Mee eens. Als er iets tussen mijn plannen komt. Mee eens. Mee eens. Het is allemaal planmatig.. je gaat van de mensen toch geen robots maken he. |
| DEEL 1 ADVIES | 9:50 - 12 | Je zou eens duidelijk stellen waar de mensen – je geeft hier een advies je kan meer actief zijn in je vrije tijd maar je kan ook kiezen voor een actievere levensstijl in de tuin, huishouden.. maar ik woon in een appartement.. dat is een groot verschil he. Vroeger kon ik dat gemakkelijk he in een villa nu niet meer.. dat geeft dan weeral een repercussie naar je gevolgtrekking hé. |
| DEEL 1 OPSTELLEN ACTIEPLAN | 12 | **Ik ga je wel vragen om op ja te klikken**. Ja allez vooruit.  Denk je dat je het moeilijk zal vinden meer te bewegen..  Weet je al waar.. dat weet ik niet hoor. Rond het appartement hier .. hoe moet je dat dan omschrijven? **‘in de omgeving van mijn huis’**. Ik heb wel een wandeling voorzien met vrienden, maar dat is niet de eerste activiteit he. Hoeveel dagen per week wil je je je eerste activiteit doen.. 3 dagen per week. Je weet dat toch niet op voorhand hoe kan ik nu plannen wanneer dat er vrij tijd gaat zijn. Ik heb een drukke agenda dus dat is onvoorspelbaar. Ik zal er dan maar een aantal dagen opzetten ofwat? **Ja dat kan je doen.**  Dat ga ik toch niet doen hoor, dat is niet op mij van toepassing. Als-dan. Als ik thuis kom- als ik op zondag namiddag.. dat ga ik niet invullen. **Je kan gewoon ‘xx’ invullen dan.** **Het is de bedoeling dat je de datum van vandaag neemt.** |
| DEEL 1 ACTIEPLAN |  | Je moet meer bewegen zeggen ze hier! *Dat wist je toch wel he*. Ja daarvoor moet je geen dinges hebben he. *Nu is hij terug beginnen stappen he.* Ik moet dat bespreken met mijn dokter .. ik ga dat doel bijhouden in mijn agenda dat zal het gemakkelijkste zijn. Tijd voor actie! |
| DEEL 2 VRAGENLIJST | *(2^e^ fragment)* | **Normaal krijgt u dit pas na een week, het is de bedoeling dat u nu doet alsof**. Ahja.  Die fysieke activiteiten op het werk he, die zul je toch ook meer moeten specifiëren volgens mij. Ik ben gepensioneerd en ik doe vrijwilligerswerk. Dus.. jullie zeggen dat dat ook bij werk hoort. Ik zie dat niet als werk. Bijvoorbeeld als je gaat winkelen? **Ja zoiets**. Ik ben niet de nieuwe man hoor. Wandelen zit hier niet inbegrepen? **Neen omdat dat geen matige fysieke activiteit is, omdat dat een lage fysieke activiteit is.**  Dat is hier voor de buitenwerkers jong.. zwaar tilwerk, djeezes. Dus ik mag ervan uitgaan dat – **ja inderdaad dat we een week verder zijn, of u het gehaald heeft of niet dat is u eigen keuze.** Wat is dat hier? **Dat is een fout. Vind je dat goed die antwoorden of mocht daar nog een ander antwoord staan?** De mogelijkheden waarom het niet behaald is.. neu, dat is volledig volgens mij.  Kan je ons hier vertellen door welke hindernissen je het doel niet hebt behaald, tijdsgebrek.. en nu zie ik hier juist hetzelfde staan in het tweede? Dat moet je dit toch niet aanhalen?  Dat zal dat tweede zijn he .. snowboarden dat zie ik mij nog niet doen hoor. |
