# Supplementary material for: Users’ thoughts and opinions about a self-regulation-based eHealth intervention targeting physical activity and the intake of fruit and vegetables: A qualitative study
Source: PLoS One. 2017 Dec 21;12(12):e0190020. doi: 10.1371/journal.pone.0190020 (PMC5739439; doi:10.1371/journal.pone.0190020)
Supplement: S3 File — This file contains the transcribed interviews. (ZIP) [file pone.0190020.s003.zip › type_2_diabetes/TA2BODO.docx]

**Code filmpjes:**

| Deel interventie | Minuten | Transcript |
| --- | --- | --- |
| DEEL 1  VRAGENLIJST | 0-11 | Moet ik alle drie die doen? Ah eentje aanduiden. Persoonlijk advies .. alleen de voornaam of? *(vult gegevens in)* ik fiets veel naar mijn werk, maar ik denk dat dat niet bedoeld wordt met hetgeen hier uitgesloten is, het is niet helemaal duidelijk.. op hoeveel dagen fiets je 10 minuten aan een stuk. 6 dagen. het is mij niet helemaal duidelijk of hier bedoeld wordt alle fietstrajecten of 1 fietstraject dat je gebruikelijk doet van heen én terug.. maar ik ga nu gewoon alle fietstrajecten nemen. dan is dat gemakkelijk 30 minuten. Welk tempo.. middelmatig.  Hoeveel wandel je gewoonlijk .. ik ga nogal veel gaan wandelen op zondag dus dan is dat gemakkelijk 2 uur .. vrij hoog tempo. Ik twijfel tussen hoog als middelmatig, ik ga het bij hoog houden.  Fysieke activiteiten in huis die minstens 10 minuten aan een stuk duren.. tgoh.. 10 minuten fysieke activiteit in mijn woning, dat lijkt mij.. laat ons zeggen op een drietal dagen. ik heb geen tuin dus – heb je niet de keuze om hier nul aan te duiden? **Als je naarboven gaat, deze vraag is in de tuin of in de moestuin.** Ah ja oei! Dat had ik niet gezien.. dat is dan geen. Dat is dan ook geen natuurlijk.  Lichte lasten dragen schrobben of vegen .. daar kom ik zeker geen 10 minuten aan maar het is mijn niet duidelijk of een beetje met gewichten trainen ofzo daar ook bijhoort. **Het is huishoudelijk werk dus dat hoort er niet bij.** Dus geen. Dus je moet toch wel strikt interpreteren. Oke.  Uitsluitend als recreatie .. fietsen aan een middelmatig tempo, zwemmen .. euhm .. het is mij – ik vermoed dat wat ik hier als eerste heb ingevuld gerelateerd aan het werk dat dat hier niet meetelt.. dus dan is het meer als hobby ofzo.. en goh, dan is dat toch op 4 dagen. hoeveel tijd .. 1 uur en 30 minuten. Dit is in de week, dit is algemeen, dit is specifiek in de week denk ik .. dat is dan op 3 dagen.  Hoeveel tijd besteed je gewoonlijk op zo een dag aan zware fysieke activiteit in je vrije tijd, is dat niet hetzelfde? Ah matig! Ah oeps. Dat is hier niet goed, ik moet dat veranderen. Zware fysieke activiteiten.. dat is op 4 dagen.. en dat was een uur en 40 minuten. Op hoeveel dagen wandel je 10 minuten aan een stuk in je vrije tijd, dat is 2 uur. Maar het verwondert mij dat ik daar nu weer op uitkom want ik heb dat al ingevuld. Dat is een hoog tempo. Dat verwondert mij dat ik dit nog eens moet invullen want ik heb het al ingevuld, het lijkt mij elkaar te overlappen.. maar misschien is dat niet helemaal juist. Werk je momenteel gratis of betaald.. ah oei neen ‘werk je momenteel’, ja. ik dacht eerst dat ze vroegen van werk je als vrijwilliger ofzo, maar nu zie ik dat ze gewoon vragen werk je en dan tussen haakjes vrijwillig of betaald.  Activiteiten als deel van mijn werk .. maar dat fietsen is daar niet bij inbegrepen? **Neen.** Mijn job is nu niet zo zwaar fysiek he, dus neen. Op hoeveel dagen in de gewone week wandel je 10 minuten aan een stuk als deel van je werk – dat is toch wel – ah neen maar het moet aan een stuk zijn! |
|  | 11- 19:40 | Aan een stuk wandelen euhm .. neen ik denk niet dat het 10 minuten er in zit. Hoeveel beweeg je denk je, ik denk dat ik toch wel vrij veel beweeg. Hier is het wel niet duidelijk of ze bedoelen in relatie tot je leeftijd ofzo. Moet ik mezelf hier vergelijken met leeftijdsgenoten, want dat is het heel veel. Maar los daarvan denk ik toch wel dat ik veel beweeg. Zou je graag nog meer bewegen.. ja dat is moeilijk, ik liep vroeger veel maar ik kreeg last aan mijn knie en ben geopereerd en ik mag niet meer lopen. Dat zal dus niet zo gemakkelijk zijn. als ik meer beweeg dan is mijn kans op het ontwikkelen van ziektes – ja maar als je het al hebt.. algemeen lijkt mij dat helemaal juist maar ik ga dan ook in het algemeen interpreteren. Want ik heb al diabetes he anders zou ik hier niet zitten. Anders zou deze versie niet bedoeld zijn voor diabetespatiënten. Zal ik mij mentaal beter voelen.. dat is voor mij tussen juist en helemaal juist. Ik ga daar helemaal juist van maken. En zullen anderen mijn steunen, wel als de mensen merken dat je iets aan de kwaliteit van je leven probeert toe te voegen op een gezonde manier dan – ja. volgende. Ik ben er zeker van dat ik meer kan bewegen dan dat ik nu doe. . ja. ik denk dat dat juist is. Meer kan bewegen in moeilijke situaties bv regen of vrije tijd. Die tijd is soms wel een probleem maar dat is eigenlijk gewoon een kwestie van organisatie, dus zal ik ook maar juist aanduiden. Ook als ik telkens opnieuw moet proberen: dat denk ik niet dat ik mij daar veel ga mee bezig houden met sporten waarvan ik merk dat het niet lukt – tenzij men hier bedoelt van ik neem het mij voor van te gaan bewegen maar het lukt niet altijd en ik blijf het toch proberen.  Ik ga het zo interpreteren in de tweede betekenis. Ik ga dat dan ook maar ‘juist’ aanduiden. Ook als ik problemen en zorgen heb, ja dat is zeker juist. Ook als mijn vrienden/familie dat niet doen. Dat is zeker juist. Ik ben onafhankelijk van mijn familie en zeker van mijn vrienden in hoeveelheid beweging dat ik doe.  Mezelf doelen stellen .. ja de doelen stellen vind ik niet moeilijk, maar ze realiseren is soms wat anders. Dus ik zal schrijven waarschijnlijk niet. Ik heb er moeite mee om plannen te maken die mij helpen - ik denk wel dat mensen soms nogal moeten nadenken over de antwoorden die ze geven want als je dat nu in negatief en positief zou verwoorden ‘ik heb er moeite mee’ is eigenlijk iets negatief en moet je daar dan nog iets negatief op antwoorden of wordt dat dan positief – ik denk denk dat dat helderder kan of dat veel mensen dat verkeerd zouden interpreteren. Iemand die dat zo invult voor eens te kijken wat hij uitkomt en die dat niet zo aandachtig doet zoals ik het nu probeer dan denk ik dat het voor interpretatie vatbaar is en tot verkeerde antwoorden kan leiden. Ik heb er moeite mee op plannen te maken mijn plannen om meer te bewegen te bereiken, misschien wel misschien niet. Dat moet nog blijken. Ik hou mijn voortgang bij, neen. Toen ik nog liep hield ik de tijd in de gaten om te kijken of ik er op vooruitging wat snelheid betreft, maar bijhouden of ik meer beweeg ofzo nee dat doe ik niet.. ik heb een duidelijk plan voor wanneer: juist. Voor hoeveel: dat plan heb ik wel .. dat is ook juist. Als er iets tussen mijn plannen komt: dat een beetje een moeilijke interpreteerbare vraag vind ik. Moet ik dat opvatten als je kan niet gaan sporten, probeer je die tijd dan te vullen met iets anders los van het werk of betekent dat dat je die specifieke vorm van beweging die dag niet kunt uitvoeren en of ik dan een duidelijk plan heb om iets anders te doen? Bv. ik ga gaan lopen maar het regent, dus ga ik gaan fitnissen? Ook dat is mij niet helemaal duidelijk. Maar of ik zo een plan heb, neen. In situaties waarin het moeilijk is om meer te bewegen, neen dat heb ik niet want ik zie niet in welke situatie waarin ik zou terechtkomen waarin het moeilijk is – dus neen. |
| DEEL 1 ADVIES | 19:40 – 21:20 | (*leest advies voor ‘meer actief te verplaatsen’)* ja maar ik verplaats me wel actief he. Ik zie niet direct dat dit besluit gelieerd is aan de antwoorden die ik daarnet heb ingevuld want ik verplaats mij actief met de fiets, en soms te voet. Actieplan ... ja dat zal wel lukken. |
| DEEL 1 OPSTELLEN ACTIEPLAN | 21:20 -32:50 | Denk je dat je het in bepaalde situaties moeilijk zal vinden om meer te bewegen? Die formulering komt regelmatig terug en ik weet niet hoe ik die moet interpreteren, letterlijk als meer bewegen dat dat ik nu doe, of eerder in de zin van te bewegen zoals ik het nu doe. Maar hier staat er dus meer.. dus als dat meer moet zijn dan denk ik dat ik in tijdsnood ga komen voor mijn werk dus ik denk ja, als het gaat over meer bewegen dat dat niet altijd eenvoudig zal zijn. welke opties kunnen hindernissen zijn.. een gebrek aan zelfdiscipline soms. Een blessure.. ja. de belangrijkste hindernis.. dus 1 belangrijkste hindernis? Ik ga dan een gebrek aan tijd nemen.  …  Het zou ook wel interessant zijn dat je feedback krijgt over hoe je de tijd die je reeds besteed aan bewegen hoe je die efficienter – hoe je er meer output van zou krijgen. Dat gaat misschien meer naar trainingstechnieken dus misschien te ver. Maar dat zou mij wel meer interesseren van hoe kun je de tijd die je gebruikt optimaal benutten om je conditie op pijl te houden of je gewicht. Volgende.  Hoe wil je vooral .. door zowel in je vrije tijd actief te zijn als actieve levensstijl ja.  **Je duidt best dit aan anders ga je veel extra werk hebben, anders moet je twee dingen kiezen.**  Ah oke, dat is wel niet zo duidelijk dan.  Op welke manier.. daar ben ik benieuwd naar hoe ik op het werk actiever kan zijn want actief verplaatsen dat doe ik al, en de tuin heb ik niet, en het huishouden helaas daar ben ik niet actief genoeg in. Om het werken zelf wat actiever te maken .. 1 keuze of? Kan ik er verschillende? Dan trap nemen denk ik .. ja je kan er dus verschillende .. als ik middagpauze heb eventueel .. dat zal misschien wat raar overkomen voor de persoon die dat leest want als je voortdurend tijdens het werk wilt sporten hoe kun je dan nog werken natuurlijk he. Waar ik bedoel ‘voortdurend’ als de gelegendheid zich voortdoet he. Zelf een als-dan plan op te stellen .. bij de ‘als’ geef je aan wanneer je nu precies wilt bewegen, bij de ‘dan’ .. waarom niet ‘wanneer’ in plaats van ‘als?’ .. beide. Met de informatie die ik tot hier heb zou ik daar eeder van maken ‘wanneer’-‘dan’ plan. Misschien is dat bedoelt als ‘als’ de gelegenheid zich voordoet ofzo.. ik hoop dat het geen verkeerd gebruik is van de term ‘als’ zoals ik ga eens ik kijken als ik post heb. Ik hoop dat het geen taalfout is van dat zou toch niet mogen dan he. Voor mij zou ‘wanneer’ duidelijker zijn.  Ik weet niet of er verder nog suggesties komen van als je een verdiep hoger moet neem de trap ofzo .. nee bon, we gaan volgende nemen. vanaf wanneer. **Het is de bedoeling dat u de dag van vandaag ingeeft voor de studie.** |
| DEEL 1 ACTIEPLAN | 32:50 | Ahja, dat is wat ik ingevuld heb. Te bespreken met je arts, ja. goh ja, ik ga dat naar mijn vrouw sturen he. Dat is moeilijk he om te bepalen in mijn geval of ik mijn doel bereikt heb en als ik het bereikt heb moet ik ze niet opschrijven he .. ik zie niet direct het nut in van te beginnen turven van ik heb zoveel keer de trap genomen.. ik wil liever niet elke dag bijhouden. Tijd voor actie; |
| DEEL 2 VRAGENLIJST | *(2^e^ fragment)*  0-10 | Dus weer hetzelfde he eigenlijk? Moet ik dat nu nog allemaal eens invullen? **Ja nu willen ze kijken of je meer beweegt tov de vorige keer.** Aaah ja! juist. Dezelfde bedenkingen bij de vragen.. laat ons 40 minuten zeggen. **Dan rekent hij wel 6 dagen 40 minuten he.** Ah ja. dan zet ik 5 dagen. **het is gewoon dat het juist is**. Het is op de dagen dat ik op mijn ander bureau zit fiets ik soms meer soms minder dan 40 minuten.  Wandel je 10 minuten aan een stuk ..  Misschien moet dat meer herhaald worden dat het over het werkgerelateerd vervoer gaat en niet vrije tijd want ik was het alweer vergeten..  Hoeveel wandel je op zo een dag .. 20 minuten. Welk tempo .. minstens 10 minuten in en rond het huis he. Geen. Nog steeds. Ook geen. We hebben een huis met veel trappen.. laat ons zeggen op 2 dagen dat ik veel trappen moet doen. Binnenshuis aan matige – is dat nu nog steeds aan het huishouden gelieerd? **Ja**. 10 minuten.. in je vrije tijd .. later komt er nog een vraag van zware he? **Ja.**  4 dagen .. hoeveel tijd besteed je.. een uur en 40 minuten.. in mijn vrije tijd.. 2 dagen. ja. dat is een beetje moeilijk want bij mij zit er veel verschil. Ik ga soms naar het stad op mijn vrije dag en dan stap ik wat rond, in mijn vrije tijd is dat meer gericht gaan stappen om te bewegen. Op zaterdag is dat misschien een halfuur, op zondag gemakkelijk 2 uur. Moet ik dan het gemiddelde daarvan nemen? **Ja.** laat ons dan zeggen.. eerder middelmatig he.  10 minuten aan een stuk .. geen. |
|  | 10 – 15 | Dat is ook aan een stuk? **Ja**. neen ja dus dat is dat. Nog niet volledig geslaagd.. aaah ik heb maar vier dagen, ja juist. Mijn doel was te hoog.. misschien – eigenlijk had ik het nu enkel op trappen nemen maar ik kan op alle dagen stretchen he op het werk, dus – ik zou niet in waarom ik zou moeten stretchen als ik geen activiteit heb.. je kan ook lichte turnoefeningen of je rug ofzo wat los maken.  Proficiat – je bent geslaagd in je eigen doel. 84 minuten per week .. Het is goed dat het in groot lettertype is, dat nodigt uit om het te lezen, als het kleiner zou zijn is het moeilijk te lezen. Maar het neemt best wel wat tijd in beslag en je moet toch wel aandachtig lezen ook. En nu hebben we geluk maar als je dat invult ’s avonds en de telefoon gaat of op kantoor en iemand komt binnen, het kan zijn dat je afgeleid wordt, misschien moet er kernachtiger geformuleerd worden.  Een nieuw doel stellen , een hoger doel stellen … je kan ook kiezen om beide te doen.. neen een nieuw actieplan ik denk niet dat er voor mij nog veel rek op zit. dan is het de bedoeling dat je regelmatig aan zelfcontrole doet? **Ja.** maar volgens mij zou het nuttiger kunnen zijn – maar dat is veel meer werk he – nu moet je zelf zeggen van daar kan het beter, dat je de inhoud van je job beschrijft, het aantal uren aan het bureau, en dat er van daaruit praktische tips komen vanuit de applicatie ofzo van ja, dat is uw situatie, je zou kunnen dit of dat doen. Nu moest ik zelf zeggen van ik zal de trap nemen, maar misschien zijn er nog andere mogelijkheden waar ik niet aan denk he. En dan feedback en suggesties geven .. maar dat kan heel veel werk zijn.. maar het zou het wel aantrekkelijker maken.  Wat ga ik doen .. ja dat is de samenvatting weer. Volgende. Versturen? **Ja**. |
