# Supplementary material for: Users’ thoughts and opinions about a self-regulation-based eHealth intervention targeting physical activity and the intake of fruit and vegetables: A qualitative study
Source: PLoS One. 2017 Dec 21;12(12):e0190020. doi: 10.1371/journal.pone.0190020 (PMC5739439; doi:10.1371/journal.pone.0190020)
Supplement: S3 File — This file contains the transcribed interviews. (ZIP) [file pone.0190020.s003.zip › type_2_diabetes/TA2BOEI.docx]

**Code filmpjes:**

| Deel interventie | Minuten | Transcript |
| --- | --- | --- |
| DEEL 1  VRAGENLIJST | 0-10 | Ik ga beweging pakken. *(vult persoonlijke gegevens in)*  Op hoeveel dagen fiets je 10 minuten aan een stuk om ergens heen te gaan.. eigenlijk geen, ik ga altijd met de auto.. wandelen, toch wel 3 dagen denk ik .. in het weekend doen wij lange wandelingen aan de zee. Ik zal een gemiddelde geven van 30min. Op mijn gemak, laag tempo.  … tuin of moestuin, niet veel. Ik ga een gemiddelde pakken op jaarbasis, 1 dag. Het is voornamelijk in de zomer.  …  Ik ging om de 2-3 dagen op mijn hometrainer maar dat is volledig stilgevallen. Ik zou dat opnieuw willen doen. Ik ga 2 dagen zetten. Meestal 40 minuten. Zwaar fysieke activiteit geen, ik probeer dat echt te vermijden omdat ik diabetes heb en een te hoge bloeddruk. De dokter heeft mij dat afgeraden. |
|  | 10-18 | Telt dat mee zo over en weer met mijn gerief? **Ja ik denk niet dat dat 10 minuten aan een stuk is**… ja inderdaad. Als je dat als leidraad pakt is dat inderdaad niet zo. Ik probeer wel altijd de lift te vermijden en de trap te nemen.  Hoeveel beweeg je denk je. Weinig. Ja eigenlijk wel. Ik ben 7 maand geleden van werk veranderd dus ik heb een heel drukke inloop periode gehad.. soms dagen van 14-15 uur. De laatste 7 maanden kan je niet vergelijken met daarvoor. Ik ben ook veel bijgekomen dus ook om het feit dat ik veel op restaurant ga voor het werk. Als ik meer beweeg is mijn kans op ziektes kleiner: is dat een gewone vraag hier? Dat klopt he. Zullen anderen mij steunen, ja zeker. Ook in moeilijke situaties.. ik denk het wel, waar een wil is is een weg. Ook als ik opnieuw moet proberen ja. Ook als ik problemen en zorgen heb, dat denk ik niet. Ik ben nogal een piekeraar. Ik probeer die problemen gewoon op te lossen. Ook als mijn familie/vrienden dat niet doen.. dat is een beetje moeilijk he! Want het is gemakkelijker als ze meedoen. Maar ik ben ben iemand die zich daarvoor kan inzetten. Ik heb er moeite mee doelen te stellen.. nee. Plannen te maken .. ook niet. Ik hou mijn voortgang bij, ja dat doe ik altijd op een zelfgemaakte file op mijn smartphone. Ik hou bij hoeveel minuten ik doe, calorieën noem maar op. Dat is wel een stimulans hoor. Dan zet ik dat in grafieken enzo.  Ik heb een duidelijk plan: hmm hmm .. ik ga altijd voor 45 minuten, dan voel ik mij goed. Langer gaat dat tegensteken en korter helpt niet. Dus 45.  Ook niet. |
| DEEL 1 ADVIES | 18-22 | Matige tot zware intensiteit, hoe kom je daar aan? Het is goed dat je beweegt … gezondere levensstijl .. dat is wat de dokter ook zegt tegen mij, maar je moet het kunnen doen he. Meer bewegen op het werk in de tuin, dat is moeilijk he. Ik kan moeilijk een blokje rond gaan doen he. In de tuin gaat wel. Ja ik zou wel een eigen actieplan willen opmaken! |
| DEEL 1 OPSTELLEN ACTIEPLAN | 22 – 32:15 | Denk je dat je het soms moeilijk zou vinden zeker wel.. zelfdiscipline ja, als je ’s avonds thuis komt en je bent moe .. het is niet gemakkelijk als je overgewicht hebt om op die fiets te kruipen en een half uur – drie kwartier bezig te zijn.. omdat je je soms ongemakkelijk voelt he. Niet moe, niet snel uitgeput. Wat is de belangrijkste hindernis.. is er maar een dat je kan aanduiden? **Ja.** Gebrek aan tijd. *(leest tips voor)*  **Het eerste is als je zegt met de fiets, want dat is de vrije tijd. Terwijl als je zegt in het huishouden of de vrije tijd is dat meer het tweede**.  Fietsen voor mij .. fietsen en wandelen eigenlijk. Ik wandel heel graag. Mag je eentje of nog meer? **Je kan nog eentje.** Het is enorm ontspannend, vooral aan de zee. Waar .. aan de zee heb ik een fiets maar hier nog niet. Tweede.. aan de zee. Van fietsen ga ik ‘thuis’ maken want dat is de hometrainer he.  Hoeveel dagen per week.. 1x in de week. En om de 14 dagen naar de zee. Wanneer.. ’s avonds. Als ik normaal gezien kijk naar tv, want ik heb ook een tablet waarop ik tv kijk, dus dat doe ik dan terwijl ik fiets. Meestal vlak na de middag. Als het namiddag is. 45 minuten. Gedurende enkele weken dat klopt inderdaad. Als ik aan de zee ben zal het langer zijn dan 60 minuten he. Als-dan: als ik naar het journaal wil kijken, dan doe ik dat met de tablet op de hometrainer.  Als ik aan de zee ben, dan maak ik superlange wandelingen op het strand. Dat is het liefste dat ik doe! **Hier zou je de datum van vandaag moeten zetten.**  Wat leuk zou zijn aan de site is als hij bijvoorbeeld met grafiekjes enzo, dus dat je kan laten zien maandag-woensdag-donderdag en dan met de minuten.. 90 op een week. Dat je ook tabelletjes kan invoeren. Dat je zelf kan aanvullen. Bijvoorbeeld dat je een tabel kan sturen naar je eigen email. Dat je dat zelf niet moet aanmaken, bijvoorbeeld een link dat je ergens zet dat je een kant en klare excel file hebt, dat die dagen er in komen dat je hebt aangeduid. En de minuten en de calorieën. |
| DEEL 1 ACTIEPLAN | 32:15 | *(leest plan voor)* ja 35 is veel te hoog he, ja en bespreken met je arts dat klopt, maar dat doe ik al. Ik heb een héle goede dokter. Overgewicht komt ook wel wat door mijn beroep, zitten zitten en gaan eten enzovoort. En dat begint zich een beetje te manifesteren kwaaltjes enzo. Suikerziekte, hoge bloeddruk. Ik ga het versturen naar mijn vrouw. Oeioei zoveel personen, ik ga het bij 1 houden. Ik ga mijn doel bijhouden op mijn computer, dat deed ik al. |
| DEEL 2 VRAGENLIJST | *(2^e^ fragment)*  0-9:40 | Dat is hetzelfde als vorige keer? **Ja je kan nu vrij snel gaan omdat je het al eens hebt ingevuld.**  ….  *(vult vragen opnieuw in)*  Proficiat, je hebt je doel behaald .. hier zou je bijvoorbeeld een trompet kunnen doen zoals ze doen bij ryanair als ze op tijd aankomen. Is dat komma? **Dat is blijkbaar niet goed afgerond.** Het is precies het getal pi. Een nieuw doel.. ik vind dat als je die 2 dingen doet dat je al heel wat doet. Wat bedoel je hier eigenlijk mee ‘ik wil graag een nieuw plan maken’? dat is hetzelfde doel ofzo dat je voor je eigen hebt gezet? **Ja.**  Dat zou ik doen zo meer visuele dingen op de website. Je zou bijvoorbeeld kunnen zeggen ‘wist je dat’ zo van die weetjes he, ‘wist je dat een uur lopen dat dat die cola neutraliseert’ ofzoiets. |
