# Supplementary material for: Users’ thoughts and opinions about a self-regulation-based eHealth intervention targeting physical activity and the intake of fruit and vegetables: A qualitative study
Source: PLoS One. 2017 Dec 21;12(12):e0190020. doi: 10.1371/journal.pone.0190020 (PMC5739439; doi:10.1371/journal.pone.0190020)
Supplement: S3 File — This file contains the transcribed interviews. (ZIP) [file pone.0190020.s003.zip › type_2_diabetes/TA2BOEK.docx]

**Code filmpjes:**

| Deel interventie | Minuten | Transcript |
| --- | --- | --- |
| DEEL 1  VRAGENLIJST | 0-10 | *(vult gegevens in)* |
|  | 10-26 | Op mijn werk? Ik werk niet meer he! Dat is moeilijk he fiets je 10 minuten aan een stuk. Als ik fiets is het nooit minder dan 40 kilometer.. **maar hier is het enkel transport dus niet als ontspanning.** Welk tempo.. dat is nog altijd over verplaatsing? Een laag tempo.  *(vult vragenlijst verder in)*  het hangt ook af van het weer he.  ….  Hoeveel beweeg je denk je. Dat is een goede vraag. Ik denk dat het niet veel is, dat het niet genoeg is.  Ook als ik telkens opnieuw moet proberen .. ik heb plannen voor als er iets tussen mijn plannen komt .. dit is chinees. Ze maken dat allemaal zo moeilijk.  Ik stel mij geen doelen .. |
| DEEL 1 ADVIES | 26 - 28 | Dat is een gemiddelde natuurlijk he. Dat is goed. Dat hangt allemaal af van het weer. Zwemmen.. ik kan niet zwemmen dus dat is levensgevaarlijk he. **Wil je een actieplan maken .. hier zal ik je moeten vragen om ‘ja’ aan te duiden..** |
| DEEL 1 OPSTELLEN ACTIEPLAN |  |  |
| DEEL 1 ACTIEPLAN |  | *2^e^ fragment defect!* |
| DEEL 1 REST |  |  |
| DEEL 2 VRAGENLIJST | *Volledig 3^e^ fragment* | **Nu zijn we zogezegd een weekje later. …**  **…**  **het is meer om te weten wat u van de website vindt dan wat u antwoordt eigenlijk.**  **…**  **u mag eigenlijk zeggen wat u vindt van de vragen**  **ze maken er een groot verschil tussen he..** dat hangt af van u leeftijd. werken in de tuin dat doe je niet als je ouder bent omdat je rug daar niet meer tegen kan he. Als je jong bent ga je ook veel meer werken in het huishouden, schilderen enzo of verbouwen, maar als dat gedaan is dan is dat ook gedaan.  …  wat moet ik daar nu op zetten? **Je mag kiezen.** Ik heb altijd 3 dagen gezegd dus nu wat hoger he. Maar deze week heb ik het niet gedaan. **Maar dat is niet erg, je mag vooral positieve/negatieve dingen zeggen van de site.**  …  Dat is door omstandigheden he .. dat vind ik niet goed geformuleerd. Dat is te algemeen. Regen, voila. Ik zou graag zelfdiscipline aanduiden maar dat gaat niet, je kan maar de ene of de andere doen he.. het is weer van dat.. het is het een of het ander he. Het is vooral die dat ik absoluut te wienig doen. Dat is die oefening thuis he. Dat is een beetje verwarrend vind ik. **Waarom juist?** De vragen worden verschillende keren herhaald maar het is niet duidelijk van over welke activiteit ben je dan bezig.  Wat is dat hier. Waar fietsen.. waarom komt dat nu weer terug? Buiten he .. mensen moeten daar toch nog opmerkingen over gegeven hebben dat het altijd dezelfde vragen zijn? **ja dat is zeker goed dat je dat zegt**. Dat is ook weer dezelfde vraag als de eerste keer, is dat om te zien of je je eigen tegenspreekt. Het is een psychologische test of je niet teveel liegt. **Het is gewoon een pre en een post meting.**  Wéér hetzelfde he. Na de middag he .. het is altijd ’s morgens. 60 minuten .. 20 minuten .. als-dan..  Mijn actieplan. Ik doe natuurlijk meer dan dat maar wandelen staat daar niet in .. dat is te laat he.  **Je mag op versturen klikken.** |
| DEEL 2 AANPASSEN ACTIEPLAN |  |  |
| DEEL 2 REST |  |  |
