# Supplementary material for: Users’ thoughts and opinions about a self-regulation-based eHealth intervention targeting physical activity and the intake of fruit and vegetables: A qualitative study
Source: PLoS One. 2017 Dec 21;12(12):e0190020. doi: 10.1371/journal.pone.0190020 (PMC5739439; doi:10.1371/journal.pone.0190020)
Supplement: S3 File — This file contains the transcribed interviews. (ZIP) [file pone.0190020.s003.zip › type_2_diabetes/TA2BOER.docx]

**Code filmpjes:**

| Deel interventie | Minuten | Transcript |
| --- | --- | --- |
| DEEL 1  VRAGENLIJST | 0-11 | *(vult persoonlijke gegevens in) (leest inleiding voor)* ik ga eens naar fruit gaan he. En nu volgende. Naam invullen .. naam en voornaam? **Je mag kiezen. Voornaam is voldoende.**  ….  Dat heb ik toch al ingevuld? Als ik iets verkeerd doe moet je het zeggen he juffie. **Neeneen je bent goed bezig.**  *(vult verder gegevens in)*  Hoeveel dagen .. goh .. 3 dagen misschien. Oké. Hoeveel van deze fruitsoorten .. sinaalappel juist he. Appels ook, moet ik hierop klikken dan of moet ik dat invullen? **Je moet op de vakjes klikken en dan krijg je cijfertjes van 0 tot verder.** Appels mag ik niet eten het is allemaal suikers zeggen ze.. mango’s .. dat eet ik ook niet. Ja 6. Dessertschaaltjes neen, framboos neen. **Als je het niet hebt gegeten moet je nul aangeven.** Ahja? Hier ook dus?  Jah een bakje aardbeien … **hoeveel schaaltjes zouden dat ongeveer zijn?** Twee.. **dan mag je 2 aangeven**. Mandarijntjes.. dat zal wel eens gebeuren ja. **Het gaat over de voorbije zeven dagen hé.** Kersen of krieken, het is niet het moment é. |
|  | 11-18 | Mango’s ja hier staat het. Twee. Het schijnt dat daar ook veel suiker in zit. En nu? **Je mag ietsje naar beneden en terug op volgende drukken.** ahja. **je mag nog altijd spontaan zeggen wat in je opkomt.** Hoeveel fruit denk je dat je eet … als ik meer fruit eet dan.. ahja, is mijn kans op ziektes kleiner.. juist zeker he. Zal ik mij mentaal beter voelen.. dat weet ik niet. Ik denk het niet. Zullen anderen mij steunen .. **wat denkt u bij dat onderdeel, bij die vragen?** Ik zal mij mentaal beter voelen, dat dat er weinig mee te maken heeft, dat dat weinig effect heeft op mentale toestand. En zullen anderen mij steunen, ik denk het niet. Ik ben er zeker van dat ik meer fruit kan eten dan ik nu doe, ja. Meer fruit kan eten ook in moeilijke situaties.. ook als ik telkens opnieuw moet proberen tot het me lukt. Ook als ik problemen en zorgen heb .. **ik zie u kijken?** Ja oke ja .. ook als mijn familie/vrienden dat niet doen, ja dat is waar. Ik heb er moeite mee om voor mezelf doelen te stellen om meer fruit te eten .. ik heb daar geen moeite mee. Ik heb er moeite mee om plannen te maken.. plannen te maken .. ik heb daar geen moeite mee. Ik hou mijn voortgang bij.. ze bedoelen of ik dat ergens opvolg of opschrijf? **Ja.** Ik heb een duidelijk plan voor wanneer ik meer fruit ga eten. . nee daar ga ik niet aan beginnen. Ik wil dat wel opmaken zo een plan. |
| DEEL 1 ADVIES | 18 - 20 | *(leest advies voor)* wil je graag een actieplan maken om meer fruit te eten .. **wat denk je daarvan?** Dat dat positief overkomt in de zin van, dat ik er meer aandacht zou moeten aan besteden en meer op letten dat ik meer fruit moet eten, en dat ik dat moet doen en meer ga doen. |
| DEEL 1 OPSTELLEN ACTIEPLAN | 20 – 27:15 | Denk je dat je het in bepaalde situaties moeilijk zal vinden meer fruit te eten, neen, als je het echt wil eten moet je kunnen he .. een appel meenemen naar het werk. Ja fruit, desserts enzo en die yoghurt enal daar zit ook veel suiker in he. Bij de warme maaltijd neen he. Dessert staat dat daar ook op? Als dessert staat er precies niet op? **De tweede**. Aah ja ja. **Je mag nog altijd zeggen wat in je opkomt**. Ja neu, ik probeer me te concentreren op die vragen. Voila. Als-dan: moet ik hier wat aanklikken? **Neen**. Dat gaat niet lukken dat laatste. Wat moet ik hier dan invullen? Die twee hier**? Je mag voor jezelf eentje maken.** Ik ga eens kijken.. dat ik ook in de namiddag een stuk fruit zou eten.. ja ..  Vanaf wanneer.. vanaf morgen. **Vanaf vandaag aanduiden anders gaan we problemen hebben bij het verdere verloop.** |
| DEEL 1 ACTIEPLAN | 27:15 | **Wat vind u daarvan als u dat zo ziet staan?** Ik vind dat leerrijk en ik vind dat eigenlijk positief dat je er eens mee geconfronteerd wordt en dat ze er de aandacht op vestigen eigenlijk. Dat dat een keer goed is dat je serieus ingeprent wordt.  Ah ja ja.. ja we gaan dat bijhouden he. Wil je het actieplan aan iemand tonen, baja, waarom niet. Email adres, dat zou ik niet direct kunnen zeggen. **Dan kan je ook op ‘nee’ klikken**. |
| DEEL 2 VRAGENLIJST | (2^e^ fragment)  0-10 | Heb je de vorige keer een actieplan gemaakt, ja he. Hier moet je eigenlijk een week verder in de situatie plaatsen? **Ja.** Op hoeveel dagen heb je de voorbije week fruit gegeten, normaal ga ik dat iedere dag gedaan hebben he. 7 dagen. Hoeveel van onderstaande fruitsoorten .. in stuks dus.  Het is niet het moment he van nectarines**. Wat komt er bij u op als dit leest?** Awel ja dat er veel zaken tussenstaan dat ik niet mag eten he.. trosjes druiven ze zeggen dat daar heel veel suiker inzit. Er staan daar veel fruitsoorten op .. het schijnt dat kiwi’s goed zijn, dat je dat mag eten, maar aardbeien zijn niet goed, trosjes druiven.. in ieder geval, die siroop dat is allemaal suiker. **Het is daarom dat het advies dat je krijgt je best sowieso bespreekt met je diëtiste dat je dat niet zomaar opvolgt, zeker in jou geval.**  Je bent geslaagd in je eigen doel … ik ga daar meer aandacht aan besteden he, ik ga daar werk van maken van dat fruit eten. Wat wil ik.. mijn eigen doel nastreven. Je hebt ervoor gekozen om een hoger doel te stellen .. denk je daar nog steeds zo over, ja. Op hoeveel dagen wil je de komende week fruit eten, dat is toch nog altijd 7 dagen? **Ja het enige dat je nu nog kan doen is hogere porties.** Waar moet ik dat invullen? **Dat komt later, nu moet je daar gewoon terug 7 invullen.** Wanneer wil je vooral fruit eten … voila.  Waar.. hier moet ik een tekst maken? **Ja zoals daarnet.** |
|  | 10 – 15:30 | We zitten in de toekomst eigenlijk he.. **je mag vandaag ook nemen he.** We zijn een week verder zeker he? **Ja klopt.**  Hier zie je jouw nieuw actieplan.. ja dat is goed he. **Wat bedoel je daarmee?** Dat het beantwoordt aan het voornemen dat ik mij gegeven heb. **Versturen mag je doen**. Ja. |
| DEEL 2 REST |  | **Zaten er in dat actieplan dingen waarvan je zegt ik vond dat echt goed?**  Ik vond het heel positief he eigenlijk, dat werkt stimulerend dat je aandacht daar op getrokken wordt, dat je daar een dagelijkse gewoonte moet van maken he, echt ermee bezig zijn en zeggen ola ik heb vandaag nog geen fruit gegeten, ik ben het uit het oog verloren en in je agenda bijhouden he. Ja dat vind ik positief aan heel die enquete eigenlijk.  **Zijn er zaken die je slecht vond?**  Neen, bijlange niet.  **En hebt u punten ter verbetering, misschien ook in het licht van uw diabetes?**  Wel ja, dat fruit he, dat het moeilijk is dat je zegt van oké fruit eten maar welk fruit, banaan bijvoorbeeld dat kan niet, druiven dat kan niet.. je moet daarmee voorzichtig zijn. In mango zit er ook veel suiker. Fruit in het algemeen. Ik zou mij meer concentreren op een appel en een peer, dingen waarvan ik zeker ben, ik moet dat dan met de diëtiste overlopen. |
