# Supplementary material for: Users’ thoughts and opinions about a self-regulation-based eHealth intervention targeting physical activity and the intake of fruit and vegetables: A qualitative study
Source: PLoS One. 2017 Dec 21;12(12):e0190020. doi: 10.1371/journal.pone.0190020 (PMC5739439; doi:10.1371/journal.pone.0190020)
Supplement: S3 File — This file contains the transcribed interviews. (ZIP) [file pone.0190020.s003.zip › type_2_diabetes/TA2BOET.docx]

**Code filmpjes:**

| Deel interventie | Minuten | Transcript |
| --- | --- | --- |
| DEEL 1  VRAGENLIJST | 0-10 | *(vult persoonlijke gegevens in)* **u mag luidop nadenken**. Niet he.. **dan moet je nul ingeven**. Mijn vrouw zegt wel dat het fruit is dat het minste suiker bevat. Als die lichtjes zuur zijn niet, maar als ze goed rijp zijn.. dusja*. (praat redelijk onverstaanbaar)* hoeveel soorten, geen. Dessertschaaltjes.. **ja zo kommetjes**. Kiwi’s .. appel soms .. allemaal veel te zoet. Ja ik eet af en toe eens een peer, maar in stukjes he … zeker niet. Fruitmoes, ja appelmoes. 2 keer in de week. Ja als het seizoen komt .. twijfelachtig .. dat wel .. |
|  | 10-13:30 | **je mag luidop nadenken, alles wat er in je opkomt mag je zeggen.** Dat doet me denken aan die vragenlijst van op mijn werk, zo rond de pot draaien. 10 vragen stellen als je het in 5 vragen kan stellen. Dat vind ik toch. |
| DEEL 1 ADVIES | 13:30-14:00 | **Je kan dat aanklikken waarover je meer wilt weten.** |
| DEEL 1 OPSTELLEN ACTIEPLAN | 14:00 – 23:00 | Mandarijntjes ofzo.. maar je moet er ook niet mee overdrijven he. Alles heeft een gradiënt. Het zijn natuurlijk heel kleine hoeveelheden die al giftig zijn en – dat is niet te schatten. Het hangt ook af van persoon tot persoon he. Ik heb gemakkelijk zuur in mijn maag dus citroen of sinaasappel geef nog meer zuur he. En banaan is zeer zoet. Ananas at ik ook regelmatig he. Het is het enige dat ik nog veel eet is aardbeien, maar in de winter is da moeilijker. Ik zou zeker meer willen druiven eten.  Is dat niet hetzelfde?  **Dit is de belangrijkste hindernis, dus van de drie die je hebt aangeduid moet je hier de belangrijkste kiezen.** Aah ja.  **Wat denkt u hierbij?** *(onverstaanbaar)*  Niet van toepassing. Ik denk dat dat het best is.  Als-dan… **ja hier kan u iets schrijven dat op u van toepassing is, zoals u daarnet had opgegeven bijvoorbeeld als ik een tussendoortje eet dan eet ik een stuk fruit.** En dat hier? **U moet juist nog de ‘dan’ invullen.** Dan wat? **Dan eet ik een stuk fruit bijvoorbeeld.** |
| DEEL 1 ACTIEPLAN | 23-25:27 | Wat moet ik hier doen? **Niets dat is gewoon om te tonen wat u allemaal heeft ingevuld.** Ik ben niet zoals mijn vrouw, ik schrijf niet alles op .. **de laatste optie ‘ik wil niet elke dag bijhouden’ is dan misschien het meest passend.** |
| DEEL 1 REST |  |  |
| DEEL 2 VRAGENLIJST |  |  |
| DEEL 2 AANPASSEN ACTIEPLAN |  |  |
| DEEL 2 REST |  |  |
