# Supplementary material for: Users’ thoughts and opinions about a self-regulation-based eHealth intervention targeting physical activity and the intake of fruit and vegetables: A qualitative study
Source: PLoS One. 2017 Dec 21;12(12):e0190020. doi: 10.1371/journal.pone.0190020 (PMC5739439; doi:10.1371/journal.pone.0190020)
Supplement: S3 File — This file contains the transcribed interviews. (ZIP) [file pone.0190020.s003.zip › type_2_diabetes/TA2BOGI.docx]

**Code filmpjes:**

| Deel interventie | Minuten | Transcript |
| --- | --- | --- |
| DEEL 1  VRAGENLIJST | 0-10 | Ik ga beweging pakken. |
|  | 10-20 | Ik werk veel in de tuin. Wandelen en sport.. jah. Fietsen is dat sport? Ja fietsen. In de tuin werken is ook fysische oefening. Huishoudwerk op het ogenblik niet want ik mag niet. Zwaar doe ik niet. Omdat ik overal nogal ver van woon maar als ik kan dan ga ik met de fiets.  Ik ben geopereerd, ik kan nu niet fietsen, maar anders toch wel 5 dagen in de week. Als is het maar naar de bakker, het is toch 2km heen en 2km terug. Ja dat is ook.. als het om te gaan fietsen is kan ik zo wel 2 uur fietsen. **Maar nu is het enkel transport.** Ja.. 20 minuten.  Ik zit toch gemiddeld tussen de 19 en 21, is dat een gemiddeld tempo? Ik heb wel een elektrische fiets he. De zondag ga ik altijd gaan wandelen met een vriendin. Alleszins drie dagen in de week dat we gaan wandelen. Ik ga zetten gemiddeld een uur. Huiselijk werk ja .. bijvoorbeeld voor het gezin tuinieren ..  Ik zit toch wel 4 dagen in de week in de tuin. Dat doe ik graag. Ik mag niet langer van de dokter voor het moment. Zware fysieke activiteiten in de tuin.. spitten doe ik wel. Gras rijden, dat is geen zwaar werk he? Zwaar werk .. spitten is ook niet alle dagen.. ik ga 1 dag geven. Doe je matige fysieke activiteit binnenshuis.. nee want ik mag niet. Voor het moment niet, anders wel. Maarja nu moet ik een beetje serieus zijn want het gaat nog wel 3-4 maand zijn tot ik terug in orde ben. Voor het moment ook niet maar anders ga ik 2x per week zwemmen, mag ik dat er op zetten? Want nu ben ik geopereerd dus gaat het niet he. Dan zwem ik 30 minuten. Op hoeveel dagen wandel je 10min in je vrije tijd.. dat is.. alle dagen he. Op hoeveel dagen wandel je minstens 10 minuten aan een stuk .. ah ja, ik ben mis?? Ik ga zetten 5 en niet 7. Dat is een kilometer of twee. |
|  | 20 – 28 | Werk je momenteel, neen he. Ik beweeg niet genoeg maarja ik kan niet. Niet veel he. Ik moet eerlijk zijn. Vanaf ik de toestemming krijg doe ik het wel maar ik krijg ze niet. Ik mag 6 weken niet met de auto rijden, dan ga ik met de fiets ook niet rijden he..  Als ik goed ga wandelen gaat mijn suiker naar beneden, dus helemaal juist. Kans op ziektes kleiner, ja he. Juist. Ik ga niet zeggen helemaal juist. Ik ben er zeker van dat ik meer kan bewegen dan ik nu doe .. ja niet op dit moment. Ook in moeilijke situaties, ja dat is juist maarja .. ik ga een beetje zeuren. Ook als ik telkens opnieuw moet proberen .. juist. **Wat vind je van die vragen?** Soms moet je eens doordenken, ik ga niet zeggen moeilijk maar toch wel .. moeilijker. Niet dat ik ze niet begrijp maarja. Er zijn vragen bij waarvan je zegt het komt op hetzelfde neer, maar het komt niet op hetzelfde neer.  Ook als ik problemen en zorgen heb, dat is zeer juist. Als familie en vrienden dat niet doen. Ja juist. Ik heb er moeite mee doelen te stellen. Dat vind ik moeilijk om op te antwoorden, ik wil wel maar ik kan niet. Ik zou wel willen een groot toer doen met mijn fiets, maar ik kan gewoon niet. Ik weet het voor mezelf niet.. ik ben opgelegd dat ik niet mag. En moeilijk om te uiten, je wilt je uiten van het gaat niet voor mij.. ik hou mijn voortgang bij.. ik maak daar geen studie van van beweeg ik meer. Ik heb een duidelijk plan voor wanneer ik meer ga bewegen.. ik heb geen plan voor het moment. Er zijn veel plannen die je maakt vind ik en die in het water vallen. Ik heb geen plan om te zeggen van ik moet dat nu doen, dat heb ik niet. Ik heb een plan voor als er iets tussen mijn plan komt. Ik heb een duidelijk plan, neen.. dat doe ik niet. Mijn verstand zegt dat ik het moet doen maar ik doe het niet. |
| DEEL 1 ADVIES | 28:10 – 32 | Ja.. het is precies nog redelijk goed. Heb ik dat ingevuld dat ik zware dingen doe? Matig he.. in de tuin.. ja. Stofzuigen mag ik niet. Dat is niet voor mij. Maar vroeger ging ik altijd naar de bakker met mijn auto, en nu doe ik dat al 2 jaar met de fiets. Maar grote boodschappen wel met de auto. **Hier moet je wel op ‘ja’ klikken voor het actieplan.** |
| DEEL 1 OPSTELLEN ACTIEPLAN | 32 – 36  *2^e^ fragment*.  0-22 | Er zijn veel vragen die terugkomen, dat ik niet goed evenwichtig kan antwoorden, niet omdat het moeilijke vragen maar het zijn precies strikvragen. Dat je zo denkt van ik denk dat maar misschien is het dadde.. soms vind ik het moeilijk. In je gedachten zeg je, ik ga zoveel gaan fietsen en dan vragen ze ‘kun je meer fietsen?’ maar op dit moment kan ik niet, maar ik heb wel ingevuld dat ik kan fietsen, dat vind ik moeilijk. Ofwel ben ik het niet gewoon van zo vragen te beantwoorden, misschien als ik dat een paar keer doe dan ga ik de vragen anders bekijken en anders antwoorden.  Want ze maken bij dat advies een beeld op en dan denk je ik heb toch niet goed geantwoord, doordat ik misschien de vragen niet goed genoeg begrepen heb ik weet het niet.  Ik heb geen gebrek aan interesse. Gebrek aan tijd dat heb ik niet neen. Het is niet dat ik het niet wil, de belangrijkste reden is omdat mijn gezondheid het niet toelaat.  Welke optie is de belangrijkste om meer te bewegen.. kies 1 van de volgende.. blessure.. ja dat is mijn rug he. Maar de dokter zegt dat dat nog niet genezen is.  Dat is het enige dat ik moet doen voor t moment, de dokter volgen.  Ik mag niet gaan zwemmen, ik wacht tot dat de specialist zegt dat ik meer mag doen.  **Je hebt en vrije tijd en actieve levensstijl. Zie!** Dat zijn zo van die momenten dat ik niet weet wat ik moet antwoorden! **Misschien moet je nu eens kijken naar iets dat je mag doen van de dokter?** Fietsen, dat ga ik toch mogen doen he in de toekomst.. maar ik ga nog eens voort kijken. Ik ga bij mijn fietsen blijven.  Wandelen he, dat gaat zowel hier zijn als aan de zee. Waar zal je jou eerste activiteit uitvoer? Op de straat zeker? Waar moet je anders gaan rijden met je fiets.. ja.. bos? Gewoon in de straat hé ja. Anders doe ik dat toch 4-5 dagen in de week.  …  Ik ga beginnen zeuren he. Dat vind ik wel moeilijk hé, omdat ik niet weet of je een toertje gaat doen met je fiets of boodschappen met de fiets dat is wel een groot verschil hé. Moeilijk op antwoorden.. **het is moeilijk inderdaad**. Ik ga 20 minuten nemen, ik ga de wandeling met men hond nemen.  Als-dan: en moet ik hierop antwoorden of zo? Ik vind dat echt moeilijk. Je kan dat plan maken, maar voor mij is het nu moeilijk. Want vandaag kan ik, en morgen kan ik dan niet. Ik ga men gedacht zeggen he. Moet ik daar nu nog iets bijzetten? **Juist nog de ‘dan’.**  Voornemens zijn er, maar op het moment gaat het niet..  Als-dan voor mijn tweede activiteit.. dat is weer hetzelfde he! Het is zo moeilijk hé. Nee ik maak het niet meer dat als-dan! Ik ga naar het volgende. |
| DEEL 1 ACTIEPLAN | 22:30 – 28:30 | Ja fietsen is op de straat he.. dat ga ik niet op de piste gaan doen. BMI is te hoog dat weet ik ! ze hebben gelijk he.  …  Ik wil men doel wel bijhouden op de computer waarom niet. Dat is wel eens goed. Dat wil ik eens wel doen! Dat je kan kijken – en krijg ik daar iets van? **Neen**. Ah het is voor jezelf hier? **Ja.** |
| DEEL 2 VRAGENLIJST | *3^e^ fragment* | **Nu zijn we zogezegd een weekje verder hé.** Dat is mijn plan? **Ja.**  Let op het gaat hier niet over wandelen en fietsen - het gaat om verplaatsen minstens 10 minuten aan een stuk. Ja.. naar de bakker, k weet het niet just. Altijd dezelfde vragen.  Snel fietsen is dat echt? Is dat dan snel? **Ja.** Ik ga zwemmen een half uur maar het is niet van ja ik moet zoveel rondjes..  **Je moet doen alsof je een week verder bent.**  Ja, ik ga dat aanduiden. Deze week niet want ik kon niet. Ik heb een beetje gelogen maar mijn doel is er hé. Ik ben content met mijn doelen. Ik moet ook niet stoeffen hé. **Voila!** **Je mag op versturen klikken.** |
