# Supplementary material for: Users’ thoughts and opinions about a self-regulation-based eHealth intervention targeting physical activity and the intake of fruit and vegetables: A qualitative study
Source: PLoS One. 2017 Dec 21;12(12):e0190020. doi: 10.1371/journal.pone.0190020 (PMC5739439; doi:10.1371/journal.pone.0190020)
Supplement: S3 File — This file contains the transcribed interviews. (ZIP) [file pone.0190020.s003.zip › type_2_diabetes/TA2BOJE.docx]

**Code filmpjes:**

| Deel interventie | Minuten | Transcript |
| --- | --- | --- |
| DEEL 1  VRAGENLIJST | 0-10  6:40 | *(vult gegevens in)* Ik kies fruit. *(vult persoonlijke gegevens in)* ik vind het wel plezant!  Hoeveel dagen heb je de voorbije week fruit gegeten.. 7 dagen .. sinaasappels ja. 2. Stekelbessen ook 1 per dag. Dessertschaaltjes neen. Dat is met het seizoen he! Ja. Oke. Dus wat moet ik dan doen? Dus 250g ik zou zeggen 300g .. ja. Moet ik hier alles invullen? **Ja als je het niet hebt gegeten moet je het op nul zetten**. Dat hangt af van het seizoen he. . kiwi’s eet ik niet veel, daar zit niet veel in he. Hoeveel fruit denk je dat je eet.. genoeg. |
|  | 10-13 | Als ik meer fruit eet dan is mijn kans op ziektes kleiner, goh .. in mijn geval he? Joat. Ik eet genoeg volgens mij. Zal ik mij mentaal beter voelen, fout zeker? Zullen anderen mij steunen?? Ik moet mijn plan trekken.  Ouh neen ! dat is niet juist! Meer fruit kan eten ook als ik problemen en zorgen heb, neen hoor. Meer fruit kan eten ook als mijn vrienden/familie dat niet doen? Huh? Dat is fout. Ik heb er moeite mee doelen te stellen: zeker niet. Ik heb er moeite mee plannen te maken, neu. Ik hou mijn voortgang bij, neen. Ik pak zeker 2 stukken per dag. **De site is gemaakt om mensen meer- maar als het niet nodig is.** Ik heb een duidelijk plan, neen ik heb geen plan. Helemaal mee oneens. Neen. Ik ben geen goede patiënt hoor. **Wat je invult maakt niet uit! Het is vooral wat je erbij zegt.** |
| DEEL 1 ADVIES | 13 – 17 | *(leest advies voor)* **nu is de site nog niet aangepast aan mensen met diabetes, je mag altijd zeggen of je vindt dat het niet klopt of niet voldoende is aangepast ofzo.** voor diabetespatiënten mag je gerust 3 stukken fruit eten, maar het is belangrijk dat je beweegt, dat is heel heel belangrijk. *(vertelt over zwemles)* ik zag als ik zwom dat het veel beter was de uitslag van mijn diabetes.  Dus fruit heeft minder belang, wat je moet doen is bewegen bewegen bewegen. |
| DEEL 1 OPSTELLEN ACTIEPLAN | 17 – 31 | Ik eet graag fruit. Meerder antwoorden mogelijk .. door fruit op mijn boterham te eten?? Ik eet geen brood, dat is slecht voor diabetes. Fruitsalade; neen. Door fruit als tussendoortje te eten, neen. Want als diabetes is het beter om fruit samen te eten met een maaltijd omdat de pieken dan hoog zijn.  Fruit bij de warme maaltijd.. courgette en paprika is dat fruit? **Neen**. Als aperitiefhapje, olijven eet ik! Druiven mag ik niet. Bosbessen die heb ik in de diepvries! Voorgesneden, dat doe ik niet, dat verliest zijn vitamines.  Door fruit op voorhand te bereiden, neen dat doe ik niet hoor.  Laat ons zeggen als ik weg ga. Allee 3, voor u een plezier te doen. Waar? Thuis. Wanneer .. Als-dan: ik weet niet hoe ik het – dat is voor domme mensen precies gemaakt .. met dat handje vanboven.. bij mij moeten ze dat niet doen. **Ja dat is goed om te weten.** Denk jij dat de mensen dat gaan doen omdat dat er op staat? **Dat is vooral wat we willen onderzoeken**. Na twee dagen zijn de mensen vergeten wat ze ingevuld hebben.. en gaan ze dat doen? Dat weet ik niet.. als ik aan de Côte d’azur zit in het zonnetje, dan zal ik meer fruit eten. |
| DEEL 1 ACTIEPLAN | 31 – 35 | Te bespreken met je huisarts ja. Bananen mag je als ze niet rijp zijn, dan zijn ze heel goed. Maar als ze rijp zijn wordt de suiker gevormd. **Ah ja oke.** Ik ga het niet versturen. |
| DEEL 2 VRAGENLIJST | *(2^e^ fragment)*  0-9 | Hebben wij de vorige keer een actieplan gemaakt? **Ja.** Het is nu een alsof het een week later is? **Ja.** Ik ga drie dagen zeggen dan. Je kan niet overal nul invullen, je moet het altijd 1 per 1 doen .. ik eet soms 3 keer per dag fruit. Ik eet feitelijk al regelmatig 3 keer. U bent op de goeie weg.. kijk eens hier, ik ben goed geweest. Je hebt aangegeven dat je onvoldoende gemotiveerd bent heh ?? dat is verkeerd. Je doel was te hoog, neen. Ik heb hier iets mis geantwoord denk ik. Op alle 7 dagen he, ahja zo, ik had het slecht begrepen.  Je bent niet in je doel geslaagd, je bent wel op de goeie weg. Hier klopt iets niet hoor. Ik heb iets verkeerd ingevuld. Ja. Niet van toepassing .. wat wil je zelf graag verder doen .. mag ik weg gaan zonder iets te antwoorden? **Wat je invult maakt nu niet zoveel uit ..**  Je hebt ervoor gekozen hetzelfde doel na te streven, je kan hier nogmaals je actieplan zien. Denk je dat dat genoeg is om de mensen te motiveren zo een vragenlijst? **Dat is een beetje waar we nu onderzoek naar doen van wat de mensen vinden van de website.** Ik vind het – niet voor mij want ik heb tijd he – ik vind dat het te lang duurt en te veel rond te pot draait. Het is te lang, ik zou het vergeten zijn! Als dat tezamen gaat met een diëtist of een dokter of in een ziekenhuis, dan kan ik me inbeelden dat het gevolgd wordt, maar iemand op zijn eigen, die vult dat in en is dat vergeten – neen dat mag ik ook niet zeggen. Er zal wel iets blijven hangen, en dat is ook al iets dat er iets blijft hangen. En het is niet genoeg concreet, het is zo goeie bedoelingen enzo maar mensen hebben meer nodig om het vol te houden.  Maar ik vind het goed he! Dat zal heel goed zijn voor veel mensen. |
