# Supplementary material for: Users’ thoughts and opinions about a self-regulation-based eHealth intervention targeting physical activity and the intake of fruit and vegetables: A qualitative study
Source: PLoS One. 2017 Dec 21;12(12):e0190020. doi: 10.1371/journal.pone.0190020 (PMC5739439; doi:10.1371/journal.pone.0190020)
Supplement: S3 File — This file contains the transcribed interviews. (ZIP) [file pone.0190020.s003.zip › type_2_diabetes/TA2BOKE.docx]

**Code filmpjes:**

| Deel interventie | Minuten | Transcript |
| --- | --- | --- |
| DEEL 1  VRAGENLIJST | 0-10 | *(invullen persoonlijke gegevens)* 300 gram groenten per dag.. ik heb daarover nagedacht, het is te zien welke groenten, want er zijn groenten die gemakkelijker oplopen in gewicht. Maar ik maak vaak verse soep, dat telt ook mee he? En fruit eten, ik pers elke ochtend 2 appelsienen. Maar gisteren heb ik daar een appel bij gegeten en mijn suiker was al veel te hoog. Dan moet ik zeker een aardappel minder nemen. Dus het is moeilijk. Bewegen, wat is bewegen. En lopen dat doe ik niet meer hé. Ik ben altijd bezig, het is niet dat ik in de zetel zit, ik doe nog alles zelf. Poetsen, schrobben, ik voel mij geen 83. ik ben 20 jaar internationaal scheidsrechter geweest bij de atletiek en nooit dik geweest, en nu toch diabetes .. **ja je kan dat niet weten he..**  …  Ik wil voor bewegen wel doen hoor. **Oke.**  Hoe heet je ..  ….. |
|  | 10-20 | 10 minuten aan een stuk .. dat zal niet zijn he. Elke dag in de week .. **het gaat over het gedrag dat je deze week hebt gedaan**. Deze week heb ik nog niet gefietst, dus geen.  Toevallig ben ik naar het stad geweest en dan neem ik tram 1, maar in het stad kom je rap aan 10 minuten he. Dus 1 dag. **U mag altijd zeggen wat er in u opkomt.** Als er beter weer is dan neem ik de fiets tot aan de tram.  Je bent rap aan een half uur he.  Welk tempo.. middelmatig hé. Ik kan niet koersen he.  Dus geen schrijf ik hier he. |
|  | 20-30 | Vloeren schrobben kuisen véél, en wat is dat hier op 1 dag op 2 dagen? Op elke dag he.. wat moet ik dan opschrijven? Op 7 dagen? **Ja!**  .. ik ben daar een half uur aan bezig en ik vind dat zwaar werk he .. volgende. Sport ontspanning en vrije tijd. Dat is gedaan he. 10 minuten aan een stuk.. ik ben niet meer in een sport, dat is gedaan. Dat is verleden tijd nu. Tijdens je werk, ik doe nu geen werk meer he. Werk je momenteel .. neen. Hoeveel beweeg je denk je, is dat nog altijd voor dat werk? **Neen dat is algemeen.** Ik vind dat ik veel beweeg omdat ik altijd rondloop he. Ben je van plan.. neen. Als ik vrij ben dan lees ik graag, ik speel ook piano. Dat doe ik liever dan – tot een jaar of twee terug gingen wij de zondag gaan fietsen maar mijn man is niet meer zo zeker op zijn fiets. Dus ben je van plan.. waarschijnlijk niet. Maar als ze zouden zeggen dat is goed voor je diabetes, dan zou ik dat wel inbouwen. Als ik meer beweeg dat is mijn kans op het ontwikkelen van ziektes kleiner.. ik heb zoveel bewogen vroeger.. dat heeft allemaal geen zin gehad. Dus ik zet fout. Zal ik mij mentaal beter voelen.. ik voel mij goed. Zullen anderen mij steunen, wat bedoelen ze daarmee? **Of anderen je gaan steunen als je meer beweegt. Of mensen daar achter gaan staan.** Ah ja, voor mij is dat niet nodig. Ik ben er zeker van dat ik meer kan bewegen dat ik nu doe, ja op dat tijdstip van een week he, en als ik mijn fiets neem automatisch beweeg je meer he .. ja. Ik vind dat niet. Ook in moeilijke situaties.. ja. Voor mij is dat omdat ik al ouder ben dat daar niet .. ook als ik opnieuw moet proberen tot het me lukt.. fout. Ik vind in mijn situatie .. oke. Problemen en zorgen heb, dat zet ik mij nog meer aan mijn piano maar ik ga niet mijn fiets nemen dan. Ik heb er moeite mee om voor mezelf doelen te stellen om meer te bewegen, mensen die door hun lichaamsgewicht, |
|  | 30-35 | dat vind ik dat zelfs al zouden ze meer moeten bewegen, dat heb ik dus niet he ik weeg 49 kg als ik meer zou bewegen dan zou je mij niet meer zien lopen! Ja dat is .. omdat mijn situatie dat niet vereist.. en ik moet hier iets antwoorden? Dat zou je in het rood moeten zetten dat je iets moet antwoorden.  Ik heb er moeite mee plannen te maken, ja moest het nodig zijn maar het is niet nodig. Dat is slecht opgesteld he. Ik hou mijn voortgang bij.. **en wat vind u slecht opgesteld?** Dat mijn situatie daar niet bij staat, en ik moet toch iets invullen.. dus schrijf maar voor magere personen dat dat ook moet hu – ja ik heb hier dus nu iets ingevuld dat geen zin heeft. Ik heb een duidelijk plan voor wanneer ik meer ga bewegen.. misschien ben ik verkeerd he dat, ik wel meer moet bewegen.. maar de dag dat ik hier 3 uur in het huishouden werk dan verminder ik mijn insuline of eet ik meer, maar dat is hier allemaal niet voorzien he. Meer ga bewegen en hier is het hoeveel .. als er bij mij iets tussenkomt dan heb ik ’s middags een heel hoog cijfer.. dusja, als er iets tussenkomt voor meer te bewegen dat zou moeten voorzien zijn in een vragenlijst, voor diabetes patiënten is dat niet goed he, op voorhand weet je al ik ga een zware inspanning doen dus minder insuline spuiten, en als er dan daar iets tussenkomt dan heb je ’s middags een heel hoog cijfer. **En heb je dan een plan voor wat je moet doen in zo een geval?** In dat geval niet wat als je dan meer eet heb je nog méér ! dan heb je een slechte planning gemaakt he, maar het is niet uw fout want er komt iemand bellen ofzo.. mee oneens. Dus je begrijpt het he? **Ja ik begrijp wat u bedoelt.** Het is de persoon die die lijst opmaakt die zal nog meer vragen moeten stellen. |
| DEEL 1 ADVIES | 35-38 | Dat is niet juist. **Ze geven een gemiddelde van wat u hebt aangegeven**. Ja maar dat is niet juist. Dat tweede deel is wel juist. **En vindt u dat het meer of minder is?** Ik denk dat het meer is. Op het werk.. niets want ik werk niet. Er staat hier niets op? **Dat klopt niet inderdaad want u huishoudelijk werk was wel veel.** Ja dat kan niet he dat is niet juist.  **Hier ga ik je moeten vragen toch een actieplan op te stellen in het doel van ons programma**. Ik ga ‘ja’ aanduiden. |
| DEEL 1 OPSTELLEN ACTIEPLAN | 38 – 47:30 | Oei oei. Dat is zo een beetje toekomst voorstellen. Als het moet kan je altijd tijd vinden hé. Omdat er hier staat hindernis.. ik ga neen schrijven. Actiever in het huishouden.. er moet dus een fout zijn he in de vragenlijst wat er stond 0 minuten. **Ja inderdaad.**  Hoho .. ik ga niet meer sporten hoor.. als die vindt dat ik niet genoeg doe zal ik dat hier ernaast schrijven hé. Tussen die twee is het niet gemakkelijk hé**. In u vrije tijd is dat inderdaad sporten en naar een club gaan gaat hij dan aanraden..**  Te voet of met de fiets naar de winkel ja als het goed weer is doe ik dat. Hier kijk, met de fiets en te voet. Op hoeveel dagen per week wilt u zich actief verplaatsen .. naar de winkel. Dat is maar 2 dagen voila. Als-dan: ah ja. Ja. **Je mag er zelf een maken of als er een van de voorbeeldjes staat die je wilt mag je die ook overtypen.** Als ik naar de tram ga, dan ga ik met de fiets. **Nu moet je eigenlijk op vandaag klikken anders werkt het programma niet meer.** |
| DEEL 1 ACTIEPLAN | 47:30 | Ja in mijn achterhoofd zit er zo van ik moet dat niet doen. **En wat vind je daar nu van?** Ja dat vind ik – mijn diabetoloog zegt ook van je bent te streng voor jezelf. Als er iets staat wil ik dat ook doen he. Moet ik dat doorsturen? **Neen dat is gewoon een optie dat je hebt.** Ik ga het toch doen.  **Je mag op versturen klikken.** |
| DEEL 1 REST |  |  |
| DEEL 2 VRAGENLIJST |  |  |
| DEEL 2 AANPASSEN ACTIEPLAN |  |  |
| DEEL 2 REST |  |  |
