# Supplementary material for: Users’ thoughts and opinions about a self-regulation-based eHealth intervention targeting physical activity and the intake of fruit and vegetables: A qualitative study
Source: PLoS One. 2017 Dec 21;12(12):e0190020. doi: 10.1371/journal.pone.0190020 (PMC5739439; doi:10.1371/journal.pone.0190020)
Supplement: S3 File — This file contains the transcribed interviews. (ZIP) [file pone.0190020.s003.zip › type_2_diabetes/TA2BOLE.docx]

**Code filmpjes:**

| Deel interventie | Minuten | Transcript |
| --- | --- | --- |
| DEEL 1  VRAGENLIJST | 0-10 | *(vult gegevens in)* die verhoudingen worden aanbevolen in elk boek of studie die zijn juist. Leeftijd 74. Dus beweging hé. **Je mag zelf kiezen.** Is een beroerte ook een infarct? **Dat weet ik niet**.. want infarct is héél breed he.. ik ga ja invullen.  Ik kan gewoon niet fietsen, daar heb ik nog altijd spijt van.  Toch een 3-tal keer in de week. **Je mag altijd zeggen wat er in je opkomt.**  Veel langer dan 20 minuten gaat niet door het feit dat mijn conditie achteruit is gegaan maar dat moet dringend veranderen. Wel eerder een laag tempo.  Neen want ik heb geen tuin dus dat gaat niet. Dat kan ik ook niet meer. Ruiten wassen, schrobben vegen stofzuigen.. want ik ben geopereerd aan mijn schouders. Sport .. jammer genoeg niet. Ik ben geen sportief mens. Dat is ook neen. En wandelen dat doe ik wel gelukkig, nog iets. Wandelen kan ook zijn in het stad rondlopen he? **Ja inderdaad.**  Tijdens je werk, ja ik werk niet meer. Ik denk dat ik weinig beweeg momenteel. En ik ga proberen om de volgende maand dat beter te doen. Ik denk inderdaad er bepaalde dagen zullen zijn dat het niet moeilijk is en andere wel. |
|  | 10-14:15 | **En wat denk je bij die vragen?** Ze zijn logisch, en het geeft wel een idee van wat een bepaalde doelstelling kan zijn om het beter te doen.  Ik denk als je veel beweegt dat je psychische gesteldheid beter is. Zullen anderen mijn steunen .. dat weet ik niet goed of dat echt belangrijk is. Daar twijfel ik**. Het gaat over je eigen mening.** Ik kan inderdaad veel meer doen dan nu. Dat klopt. Inderdaad we moeten een beetje doorzetten he. Dat lijkt mij ook juist. En dat is ook juist, je moet niet kijken naar andere mensen maar je eigen idee volgen. Euh.. die doelen stellen dat is niet moeilijk. Dat plannen maken dat zal- dat gaat wel. Of ik dat nu echt ga opschrijven .. dat denk ik niet. Ik denk dat ik dat automatisch wel zal voelen of mijn conditie beter wordt of niet.  Daar ben ik het mee eens, want ik had dat plan al. Daar ben ik het ook mee eens. Dat weet ik niet of ik een alternatief heb. Daar weet ik ook niet goed.. |
| DEEL 1 ADVIES | 14:15-15:50 | **Wat vind je van dat onderdeel**? Het is goed, het is juist wat er staat. Maar het is zeker niet op mij van toepassing want de voorbeelden die ze hier geven zijn juist twee voorbeelden die ik niet kan.. ik kan niet fietsen en ik kan niet zwemmen. Dat is gevolg van mijn opvoeding*. (praat over de oorlog)* **Maar we kunnen dan een persoonlijk actieplan maken dat wel op u van toepassing is he.** |
| DEEL 1 OPSTELLEN ACTIEPLAN | 15:50 – 26:35 | Ja ik denk dat ik problemen ga hebben. Ik heb alleszins een probleem met mijn schouders en ik ben momenteel rap moe als ik stap. Ik heb ook rugproblemen. Dat is hier in Gent het probleem dat we hier zeer slechte voetpaden hebben, zeer slechte straten. En als je dan al pijn hebt en je hebt constant die oneffenheden. Je moet constant naar de grond kijken he. Die rugpijn en schouder is het belangrijkste.  Sport zie ik niet zitten op mijn leeftijd.. een tuin enzo heb ik niet. Dan zal ik proberen actief te zijn op een andere manier. Voorlopig zie ik geen optie om nog aan een andere sport ofzo te doen. Ik ga beginnen met meer te stappen in plaats van een tram te nemen. En ik wil toch proberen om alle dagen iets te doen. **Wat denkt u daarbij?** Ik vind dat wel een goede vraag als je een bepaalde sportactiviteit doet ofzo, maar in mijn geval.. ik moet daar geen afspraak voor maken, ik moet daar niet echt iets voor plannen dus dat kan op gelijk welke dag. **Dat is vooral voor mensen die zeggen ‘drie dagen in de week’ maar jij hebt zeven dagen ingevuld dus logischerwijs moet je dan alles aanvinken.** Ik denk dat ik het meest boodschappen doe en dus kan stappen in de voormiddag.. en in de namiddag ga ik ook regelmatig wel eens buiten. Zeker niet ’s avonds, dan ga ik niet meer alleen buiten. Ik ga starten met toch zeker 30 minuten.  Als-dan: als ik ergens naartoe ga, ga ik stappen in plaats van de tram te nemen. **Wat vind u daarvan?** Dat dat zeker haalbaar is. Het is enkel bij de verplaatsing dat ik nog de mogelijkheid heb om meer actief te zijn..  Hier te voet .. dat vind ik wel wat een herhaling van het voorgaande. Ik ga regelmatig mijn kleinkinderen brengen en halen, mag ik dat ook aanduiden? **Zeker.** **Hier zou je vandaag moeten invullen anders klopt het niet meer voor ons vervolg.** |
| DEEL 1 ACTIEPLAN | 26:35 – 28:08 | Dat klopt allemaal. Neen niemand is daarin geïnteresseerd. Ik ga dat niet noteren, ik ga dat wel voelen aan mijn conditie. **En normaal gezien zou je na een week dan een mail krijgen om te kijken hoever je zit.** **Maar we gaan dat nu doorlopen.** |
| DEEL 2 VRAGENLIJST | *(2^e^ fragment)* | In principe alle dagen .. stappen in plaats van tram dat klopt allemaal. Ze vragen hier terug die fiets, terwijl ik dat niet had gezet in mijn actieplan.. dat heb ik nu alle dagen geprobeerd. Die 20 minuten kan ik zeker volhouden. **wat vind je van dat onderdeel?** Euhm.. het verwondert mij dat ze die vragen stellen aangezien dat in mijn plan niet voorkomt. Dat is terug neen .. want ik kan niet fietsen en zwemmen .. dat doe ik wel alle dagen.. en ik ben van mijn 10 minuten naar 20 gegaan, weliswaar nog altijd aan een laag tempo. Het is wel de bedoeling dat dat op termijn gaat verbeteren. Dat kan misschien veranderen.. als dat bedoeld is voor jonge mensen is dat wel logisch. Ik heb mijn activiteit op die 7 dagen gedaan.. heeft dat een bepaalde betekenis? **Neen dat is een kleine fout in het programma.** Als ik van 10 naar 20 minuten ga heb ik mij inderdaad meer verplaatst … **je mag versturen dan.** |
| DEEL 3 | *(3^e^ fragment)* | **Dit is nu na een maand zogezegd**. Het zal hier waarschijnlijk een herhaling zijn van veel zaken veronderstel ik; dat vind ik dan wel jammer dat de vragen in dat vervolgdeel niet meer typisch naar je eigen planning gaan.. niet dat dat nu te lastig is van dat eens aan te vinken, maar euh.. dan zou ik nu toch minstens aan 40 minuten moeten zitten he om goed te zijn. Ik vind na een maand dat ik minstens aan 40 .. dat ik zonder probleem aan een stuk kan – dat is wel mijn doelstelling. Het is dus wel zo als je enkel kookt en je afwas doet heb je niet zoveel beweging he. En dan zou ik toch moeten komen aan een middelmatig tempo, dat vind ik wel dat dat nodig is. Ik werk nog altijd niet.. ik hoop dat ik inderdaad – ahja het is al gedaan? **U schrikt ervan?** Ja ik had nog een paar vragen verwacht zo van wat het resultaat was van mijn inspanning, of ik mij inderdaad beter voel of het vlotter ging of .. ik vind dat dat wel nodig zou zijn dat dat erbij komt. **En zijn er dingen die je zegt van dat vond ik echt goed?** Vooral bij het begin dat men duidelijk de nadruk erop legt dat het belangrijk is te bewegen. Want ik denk dat dat wel nodig is ook naar kinderen toe enzo want er is toch wel een tendens dat we allemaal te weinig bewegen he. Want als wij ’s morgens naar school gaan dat mijn kleinkind vraagt ‘oma zou je niet eens eindelijk leren van met de auto te rijden’, er staat daar altijd een auto in reserve dus moest ik het kunnen zou ik ermee kunnen rijden, maar dat ik dan tegen haar zeg van het is goed dat je eens stapt he want anders ga je nooit sterke benen krijgen. Wat ik dan wel probeer en dat verwondert mij dat dat er niet in staat, ik zal zelden de lift nemen. Ik woon natuurlijk maar op het 1^e^, maar mijn buurman neemt veel de lift, ik neem enkel de lift als ik boodschappen bij heb.  **En zaken dat je echt slecht vond?**  Iets echt slecht kan ik niet vernoemen, die herhalingen dat ik niet nodig vond.. dat bepaalde dingen iets meer individueel gericht mogen zijn zeker naar die derde sessie toe.  Het is een beetje onpersoonlijk, het is meer zoals het van een firma uitkomt ofzo, terwijl als het echt een vorm van medische begeleiding zou worden mag er wel iets individueler.  **En heb je tips ter verbetering?**  Het voornaamste heb ik al gezegd. |
