# Supplementary material for: Users’ thoughts and opinions about a self-regulation-based eHealth intervention targeting physical activity and the intake of fruit and vegetables: A qualitative study
Source: PLoS One. 2017 Dec 21;12(12):e0190020. doi: 10.1371/journal.pone.0190020 (PMC5739439; doi:10.1371/journal.pone.0190020)
Supplement: S3 File — This file contains the transcribed interviews. (ZIP) [file pone.0190020.s003.zip › type_2_diabetes/TA2BOLT.docx]

**Code filmpjes:**

| Deel interventie | Minuten | Transcript |
| --- | --- | --- |
| DEEL 1  VRAGENLIJST | 0-10  *(Fragment 1-2-3)* | Hoe oud ben je.. dat heb ik daarnet ingevuld .. nu gaan we beginnen he? Oke. *(vult persoonlijke gegevens in)* nog een keer wat is je leeftijd??  Schaaltjes fruitsla, nooit.. appels peren, dan moet ik daar het aantal opzetten? **Ja.** Peer, 7, bessen 0. Ik vond ze nog te duur deze week. Aardbeien zijn niet het seizoen. Mandarijnen of pruimen ma seg.. het is niet het seizoen. Kiwi’s 2. Gisteren. Mango neen.. meloen lychee.. lychee is mégaveel suiker. Allemaal nul. Natuurlijk, dat gaat wisselen als het seizoen verandert he. Nooit uit blik of glas. Fruitmoes nooit. Ik ben nogal into peren.  Hoeveel fruit denk je dat je eet, niet veel niet weinig. Elke dag 1 stuk. Meer fruit.. waarschijnlijk wel. Hoe moeilijk of makkelijk denk je dat het is .. tja.. ik pak s avonds geen dessert dus ook geen fruit, maar ’s middags zou ik dat in principe kunnen doen. Zal ik me mentaal beter voelen.. pfff joa. Ik vind dat tussen de twee. Zullen anderen mij steunen? Omdat ik meer fruit eet? Baneen, dat is iets voor mijzelf. Dat ik meer fruit kan eten dan ik nu doe, ja. Ook in moeilijke situatie .. hmm. Ja dat is juist. Ook als ik problemen en zorgen heb, hmm, ik vrees dat ik dan niet naar fruit ga grijpen. Ook als mijn vrienden dat niet doen, dat trek ik mij niet aan. Wat mijn vrienden doen of vinden. Ik heb er moeite mee doelen te stellen. Doel op zich is niet moeilijk om te stellen, om te realiseren dat is wel moeilijk. Moeite om plannen te maken, joaa.. ’s morgens lukt mij dat nu om dat te eten, ’s middags .. ik hou dat niet bij. Dat lukt. Maar ik hou het niet op papier ofzo bij. Neen. Daar begin ik niet aan. Als er iets tussen mijn plannen komt: neen. In situaties waarin het moeilijk is om meer fruit te eten: heb ik niet. |
| DEEL 1 ADVIES | *(4^e^ fragment)* | Deel 3, het gezondheidsprogramma*. (leest advies voor)* actieplan maken. Eerst nog wat informatie lezen over fruit, neen. |
| DEEL 1 OPSTELLEN ACTIEPLAN | *(4^e^ fragment)* | Hoe, wanneer en met wie. Met wie?? Met mijzelf! Moeilijk om meer fruit te eten.. soms. Welke opties zijn hindernissen: ik vergeet af en toe.. ‘vaak’ is iets te sterk uitgedrukt. Wat is de belangrijkste hindernis.. dus ik moet er 1 invullen? Oe maar dat zijn allemaal dezelfde? **Ja dus van degene die je hebt ingevuld de sterkste**. Ja ik vergeet ‘vaak’ mijn 2^e^ stuk te eten.  …  ’s ochtends wil ik geen fruit eten om 10uur gaan mijn waarden veel te hoog om 11:30 en dat geeft geen correct beeld. Vandaar dat ik om 10u niets eet. Ik wil mijn curve niet verpesten om dan ’s avonds een vertekend beeld te hebben. In de namiddag is dan misschien een mogelijkheid. Als-dan: als ik thuis kom, dan eet ik een portie fruit. Dat vind ik al vrij behoorlijk. Vanaf wanneer.. **je zou vandaag moeten ingeven anders blokkeert het systeem**. Oke. Fruit klaarlegen, ja dat helpt wel. ’s Morgens mijn peer klaarleggen, dan zie ik hem al liggen. Ik zal het in mijn diabetes boekje schrijven. Wil je dat tonen? Pffff.. echt niet. Wat anderen denken interesseert mij niet. Tijd voor actie. En nu versturen. |
| DEEL 2 | *Fragment 5* | Wat is je e-mail adres.. ja dat zal ik wel weer 35 keer weer moeten bevestigen zeker .. dus dat is nog altijd nu? **Stel je hebt een week gedaan hebt, wat zou je dan gedaan hebben.** Ah ja oke! Ik ga 5 dagen nemen, pakt dat het 2 keer niet lukt. Ja, daar gaan we weer! Seg. Het gaat allemaal weer hetzelfde zijn he, 10 stuks. Kersen en krieken, dat is allemaal nul. Als dat nu 2 maand later zou zijn. Mango, dat is superveel suiker ook. Mango’s en meloenen, dat is echt.. man man man. Druiven ook hoor. Fruitmoes, is dat appelmoes ofzo? **Ja.** Dat moet ik niet hebben, dat is kindereten.  Je bent geslaagd in je eigen doel.. het is wel grappig dat je dat op voorhand moet invullen maar allez.  Mijn actieplan .. volgende.. versturen, ga ik nu weeral een mail krijgen? **Ja.** |
| DEEL 3 |  | Derde module we zijn een maand verder! Hupla! Ik ga eens doen alsof het niet gelukt is, 3 dagen. 2 porties per dag. Geen kiwi’s gegeten. Vind je dat je zelf geslaagd bent, neen! Of ja, gedeeltelijk. Waarom? Ja als de studie zo is, dan kan je je afvragen wat is de haalbaarheid van de studie he. Te druk, geen fruit in huis, geen zin in, … joah. Dat is genoeg. Voila. |
| DEEL 2 REST |  | **Hoe vond je de website in het algemeen? (pos/neg).**  Tja. Ik vind hem eerder beknopt. Ik denk dat je echt al de intentie moet hebben om het te willen doen, want ik weet niet, als je niet op voorhand beslist hebt want ik wil meer fruit of groenten, denk ik niet dat die website gaat aanzetten tot meer groenten of bewegen.. het moment dat je verplicht bent door dokters of uit jezelf wilt, en dan heb je een site enzo maarja.. ik vind het relatief vrijblijvend. Ik zou doen dat je dagelijks- dat je dingen moet doorgeven. Ik zou korter op de bal spelen met mensen die het actieplan doen. Al is het dat je zegt van ‘hoe ga je dat bijhouden?’ zou je kunnen zeggen je krijgt automatisch een mailtje van ‘heb je je 2 stukken fruit gegeten ja of nee?’ en invullen welke. Anders als het een week duurt, dat je 1. Vergeet of je het gedaan hebt of niet, welke stukken, hoeveel en dat het dus een gok wordt, en dat de gegevens gewoon onjuist zijn. In veel grotere foutenmarge dan als je elke dag digitaal doorgeeft.. hoeveel heb je er gegeten en dewelke. En dat je zegt na een week, 2, 3, 4 krijg je een overzicht van alles wat je hebt doorgestuurd. Ik denk dat dat interessant is het onderzoek van mensen die gevarieerd eten of mensen die enkel banaan eten.. je hebt veel meer informatie, je kan dat in een statistiekje gieten. Dat je weet bijvoorbeeld in de week lukt het mij wel maar in het weekend niet, ik denk dat je daar veel relevantie kunt uithalen. Nu vind ik het heel vrijblijvend. |
