# Supplementary material for: Users’ thoughts and opinions about a self-regulation-based eHealth intervention targeting physical activity and the intake of fruit and vegetables: A qualitative study
Source: PLoS One. 2017 Dec 21;12(12):e0190020. doi: 10.1371/journal.pone.0190020 (PMC5739439; doi:10.1371/journal.pone.0190020)
Supplement: S3 File — This file contains the transcribed interviews. (ZIP) [file pone.0190020.s003.zip › type_2_diabetes/TA2BONA.docx]

**Code filmpjes:**

| Deel interventie | Minuten | Transcript |
| --- | --- | --- |
| DEEL 1  VRAGENLIJST | 0-10 | **Je mag nu kiezen wel van de drie je wilt kijken**. Fruit! **Awel voila, we kunnen dan voor fruit kijken.** Volgende? **Ja**. *(vult persoonlijke gegevens in)*  Hoe moet ik dat hier invullen? **Dus de vraag is hoeveel je van deze soorten je de voorbije week gegeten hebt**. Ah ja zo.  Moet dat hier ook ingevuld worden? **Hier staat er nog wat in het rood aangeduid.** Ik moet die ook allemaal invullen en daar nul in zetten .. |
|  | 10-17 | Hoeveel fruit denk je dat je eet.. heel weinig. Ben je van plan.. ja die meloen enzo. Ik eet liever zomerfruit. Gemakkelijk. Is mijn kans op ziektes kleiner .. daar weet ik geen antwoord op zo direct. Ik denk het wel ja. Ja. Ik zou me beter voelen ook. Zullen anderen mij steunen ja. Ik kan zeker meer fruit eten, dat is just. Dat mag je niet zeggen te weinig tijd, daar moet je tijd voor maken. Dat is mis he. **Er is geen juist of fout alles wat je zegt is jou mening.** Ook als ik telkens opnieuw moet proberen.. dat is juist. Ik heb er moeite mee doelen te stellen.. ja.  Ik heb een duidelijk plan .. hmmm. Joa. Ja ik heb een plan. Voor wat ik ga doen als er iets tussen mijn plannen komt.. joa. |
| DEEL 1 ADVIES | 17-18:30 | **Het advies dat gegeven wordt is natuurlijk niet aangepast aan diabetes..** mijn arts heeft gezegd dat ik meer fruit moet eten! **Oke**. **En wat vind je van dat stukje?** Joa ..  Mijn gedacht is meer 1 portie fruit alle dagen.. ik zie het niet zitten om 2x per dag fruit te eten. **Ik stel voor dat we een actieplan maken..** **dan kan je een maken dat meer aangepast is aan u.**  **…** |
| DEEL 1 OPSTELLEN ACTIEPLAN | 18:30 - 27 | Banaan eet ik niet want ze zeggen dat dat te veel suiker is voor iemand met diabetes.  Fruit als tussendoortje. Fruitsalades .. mijn huisarts heeft dat ook gezegd van de dag voordien van die diepvriesfruitjes .. dat neem ik niet. Fruit mee te nemen naar mijn werk.. ik ga niet meer werken. Als-dan. Hier moet ik niets invullen? Dat is als je er zelf 1 wilt maken. 5 dagen per week. Thuis. Ik heb eens gelezen na je maaltijd fruit eten dat dat niet gezond is van de gisting enzo .. ik ga praktisch nooit achter mijn eten dat eten. Als tussendoortje in de voormiddag ja. **En wat vind je van dat onderdeel?** Goed! **Wat komt er zo bij je op?** Dat dat wel een stimulans is om gezonder te leven. **Hier moet je best vandaag invullen.** |
| DEEL 1 ACTIEPLAN | 27 - 31 | Ik weet dat ik te zwaar ben .. ik weet het. **De BMI klopt wel niet.. 316000 ..** ah ik dacht dat het 31 was. **Neen dat getal is fout.** Bananen en druiven dat weet ik dat ik dat niet mag nemen.. mango lychee passievrucht ook niet? Dat wist ik niet. **Het advies dat gegeven wordt, je moet luisteren naar de dokter he**. Ja. |
| DEEL 2 VRAGENLIJST | *(2^e^ fragment)* | Ja ik heb een actieplan gemaakt. We gaan doen 5 dagen per week. Moet ik hier iets invullen**? Neen.** Een peer eet ik wel graag. Een halve banaan zou ik wel durven eten. Kersen ga ik nul zetten he, dat is er nog niet.  ….  **Wat komt er zo bij je op als je dat zo ziet**? Is dat nog altijd hetzelfde dat ik moet doen, fruit eten? **Ja.** Versturen? **Ja.** |
| DEEL 2 REST |  | **Dat was de tweede module. Ik heb wel nog een aantal vraagjes. Wat vond je echt goed van het programma?**  Wat er allemaal stond van fruit enzo, dan heb je meer een idee van fruit. **Dat het voorgezegd wordt?** Ja dat vind ik wel goed. En (boets?) om fruit te eten. Ja ik heb echt een boets nodig. *(volgens mij bedoelt ze boost)* en dat je op de kalender zet dus dat je ziet ik moet nog een stuk fruit eten. **En zat er iets in dat je** zegt van dat vond ik slecht van het programma? Eigenlijk niet neen. Ik vond het wel heel goed. **En dingen die verbeterd mogen worden?** Neen daar kan ik niet direct iets van zeggen.. juist als diabeet banaan en druiven… dat mag niet. Dat weet ik. Dat mag je niet eten. Anders zouden mensen misschien denken ik ga elke dag een banaan eten. **Dat mensen dat zouden volgen dan en denken dat dat wel mag?** Ja. Dat vind ik persoonlijk minder aan. |
