# Supplementary material for: Users’ thoughts and opinions about a self-regulation-based eHealth intervention targeting physical activity and the intake of fruit and vegetables: A qualitative study
Source: PLoS One. 2017 Dec 21;12(12):e0190020. doi: 10.1371/journal.pone.0190020 (PMC5739439; doi:10.1371/journal.pone.0190020)
Supplement: S3 File — This file contains the transcribed interviews. (ZIP) [file pone.0190020.s003.zip › type_2_diabetes/TA2BOND.docx]

**Code filmpjes:**

| Deel interventie | Minuten | Transcript |
| --- | --- | --- |
| DEEL 1  VRAGENLIJST | 0-10 | *(vult gegevens in)* ik eet graag fruit maar ik heb teveel ijzer. Ik moet opletten met groene groenten, omdat dat veel ijzer bevat. |
|  | 10-32  18:00 | **Je mag een van de drie kiezen waarvoor je een actieplan wilt maken.** Ik ga groenten kiezen. *(vult persoonlijke gegevens in)* wat is je leeftijd.. ze zullen het op den duur wel weten he. Dat heb ik daarnet ook al ingevuld lengte? **Ja.**  Volgende..  Op hoeveel dagen heb je de voorbije 7 dagen groenten gegeten.. alle dagen he. Hoeveel heb je gegeten van deze soorten.. een dessertbordje sla, dat eet ik nooit op. Wat moet ik dan doen? **Nul aanduiden.** Volledig rauwe tomaten. Hier zouden ze al kunnen zien van dat probleem met mijn ijzer.. van waar dat komt he, alleja als ze de link leggen. Ik lust geen tomaat. Schijfjes tomaat dat kan er nog in. Allez, 2. Rauwe wortel ja. Geraspt is dat direct veel he. Dat staat hier weeral? **Het bovenste is geraspt en dan ander is rauw.** Ik ga er 3 op zetten he. Komkommer dat lust ik wel. Eetlepels.. ik meen dat het schijfjes was. Rauw witloof gekookte maïs .. **wat denk je?** Geen. Ook nul. Wel gestoofd. Ik weet niet of dat nog komt? **Ja.** Radijzen. Als ik aan het koken ben eet ik altijd een potje radijsjes als aperitief. Eetlepels andere groenten, dat zijn geen eetlepels he.. **ik denk dat een radijs toch wel 2 eetlepels is en dan maal het aantal dagen**. Hoezo**? Het is per week he.** Aaah zo! Dan ga ik 4 opschrijven he. En dan komkommer .. eetlepels gekookte wortelen courgette bloemkool broccoli.. dat eet ik regelmatig he. Als de kinderen broccoli eten eet ik bloemkool hé. Meestal schep ik mijn portie op maar ik kan nooit bijnemen.  Niet veel niet weinig. Ben je van plan meer groenten te gaan eten de komende maand .. hmm .. misschien wel misschien niet. Waarschijnlijk wel. Hoe moeilijk of makkelijk denk je dat het is. Niet gemakkelijk. als ik meer groenten eet dan.. is mijn kans op ziektes kleiner.. dat zal wel juist zijn zeker. Zal ik mij mentaal beter voelen. Dat zal ook juist zijn. Zullen anderen mij steunen. Hmm .. neen. Ik ben niet te overtuigen. En ik ben geen grote eter. Fout.  Ik heb een duidelijk plan.. helemaal mee eens.  Ja het is nu met die diabetes he, dat is niet zo gelukkig voor de groenten he. **Ja ..**  Waarschijnlijk heb ik er geen moeite mee om plannen te maken.  Dat is soms zo dubbelzinnig he. **Ja je moet de zinnen goed lezen inderdaad.**  Ik hou mijn voortgang bij. Waarschijnlijk niet. |
| DEEL 1 ADVIES | 32-35:48 | Ik heb niet veel keuze he.. ik mag niet veel groenten eten he. Want er zijn veel groene groenten hoor. **Als je geen actieplan wilt maken zou ik het middenste aanduiden. En dan versturen.** |
