# Supplementary material for: Users’ thoughts and opinions about a self-regulation-based eHealth intervention targeting physical activity and the intake of fruit and vegetables: A qualitative study
Source: PLoS One. 2017 Dec 21;12(12):e0190020. doi: 10.1371/journal.pone.0190020 (PMC5739439; doi:10.1371/journal.pone.0190020)
Supplement: S3 File — This file contains the transcribed interviews. (ZIP) [file pone.0190020.s003.zip › type_2_diabetes/TA2BONE.docx]

**Code filmpjes:**

| Deel interventie | Minuten | Transcript |
| --- | --- | --- |
| DEEL 1  VRAGENLIJST | 0-10 | *(leest inleiding voor)* **je mag een van de drie kiezen**. Beweging. |
|  | 10-23:40 | Ik ga wel buiten met de hond.. naar de winkel ga ik eigenlijk maar 1 keer in de week. Rond het huis .. oei oei. Euuh .. **dat is ook het huishouden enzo hé.** Ahja! Dat doe ik volledig zelf. Moet ik hier iets doen? **Neen je mag naar beneden scrollen, dat is puur informatief.** Zware fysieke activiteit .. nee ze zoeteke! Oei oei. Fietsen doe ik niet meer.  Het gaat enkel over ergens naartoe gaan.. met de hond wandelen hé. Hoeveel dagen wandel je 10 aan een stuk om ergens heen te gaan.. euh.. gohja. Alle dagen hé. Dat zal een 45-60min zijn. Fysieke activiteit ivm huishoudelijk werk.. in de tuin … ik zit niet in de tuin dus .. zware activiteit in de tuin .. geen. Matig.. vloeren schrobben, vegen ..  valt dat daaronder? **Vanaf dat je sneler ademt eigenlijk.** Ja dat is als ik trappen doe, dat doe ik elke dag als ik naar boven ga maar dat is echt heel moeilijk voor mij. Maar ik doe het wel. Een uurke of een half uurke.. eigenlijk zit ik geen half uur op de trap he, ik zit boven dan. **En hoeveel keer per dag doe je trap?** 1 of 2 keer maar. **Dan zou ik 10 minuten nemen ofzo.**  Sport.. doe ik niet. Hoeveel dagen doe je minstens 10 min aan een stuk.. 7 dagen. **Maar gaat dat terug over met je hond wandelen?** Ja. **Dat heb je al ingevuld he**. Ahja oke. **Dan duid je best ‘geen’ aan anders wordt het dubbel geteld.** Ja oke.  Op je werk.. maar ik heb geen werk. Huishouden hoort daar niet bij? **Neen.**  Hoeveel beweeg je denk je. Oei oei. Tussen de twee. Wandelen doe ik wel. Niet veel niet weinig. Ben je van plan meer te bewegen, ja zeker! Ja ja, want er is 12 kilo bij dus ik heb mijn best gedaan van februari tot nu!  Ga jij mij steunen? Nee zeker**! Haha**. Wat heb ik gedaan? **Je moet de laatste nog invullen.** Ik ben er zeker van dat ik meer kan bewegen dan ik nu doe – ja zeker. Ook als ik telkens opnieuw moet proberen.. ook als ik problemen en zorgen heb.. oei oei. Ik heb er moeite mee om voor mezelf doelen te stellen om meer te bewegen.. wat moet ik daar nu op antwoorden.. ik ben van goede wil.. **misschien het middelste?** Ja. Ik heb er moeite mee plannen te maken. Ja dat wel. Ik hou mijn voortgang bij: nee. Ik heb een duidelijk plan wanneer ik meer ga bewegen.. ja een plan heb ik wel, maar het uitvoeren .. wat moet ik dan zetten? **Als je een plan hebt dan mag je dat zetten hé.** |
| DEEL 1 ADVIES | 23:40- 27:30 | Je beweegt gemiddeld 3,4 minuten per dag.. *(leest advies voor)* voila. Je hebt gekozen geen actieplan te maken. **Het is wel de bedoeling dat je eentje maakt.** **En dan volgende.** |
| DEEL 1 OPSTELLEN ACTIEPLAN | 27:30 – 42 | Ik kan echt weinig met mijn knieën.. kies voor activiteiten waarvan je voelt dat ze haalbaar zijn. Wil je proberen om meer te bewegen.. duid een voorstel aan. Hoe wil je meer bewegen..  ….  Ik deed zoiets als dansen. **Zumba eigenlijk?** Nee het is meer turnen. Niet zo zwaar als zumba. Dat heb ik een hele tijd gedaan hoor.  Alles samen is ongeveer 13 minuten. Ik zal 15 pakken. We hebben het eens getimed. Normaal zou het een kwartier moeten zijn.  Als-dan: mag ik die als overslaan? **Je mag zelf eentje maken.**  Als ik opsta, dan pff .. **dan begin ik er aan ofzo?** Ja oke. Nog een als-dan, dat moet ik niet hebben..  Te voet .. moet ik er op schrijven te voet? **Zoals je zelf wilt**. Als je nog een extra plan wilt maken kan dat hier.. neen neen..  **Hier zou je vandaag moeten ingeven voor het systeem**. |
| DEEL 1 ACTIEPLAN | 42 – 44:42 | Ah ja hier moet ik niets invullen? **Neen dat is het overzicht.**  Versturen, neen hoor, ze lachen hier al genoeg, het zal gaan!  In een boekje bijhouden want op de computer zit ik niet graag. Tijd voor actie. |
| DEEL 2 VRAGENLIJST | *2^e^ fragment*  0-10 | Heb je de vorige keer een actieplan gemaakt om meer te bewegen, wat moet ik hier zeggen? **Ja dat hebben we gemaakt**.  Op hoeveel dagen wandel je tenminste 10 minuten.. huishoudwerk, klussen, gezinstaken.. in de tuin.. dat doe ik niet hé.  Heb je deze activiteiten de voorbije week uitgevoerd.. moet ik ja zeggen? **Ja. Stel dat we een week verder zijn he**. Ahja. 2 dan hé. Moet ik ja zeggen? **Ja.**  Je bent niet volledig geslaagd in je doel .. euhm. Nul minuten.. **dat is wel raar, dat zal fout zijn want je hebt ingevuld dat je gedanst hebt..**  Versturen? **Ja.** |
| DEEL 2 REST |  | **Wat vond je in het algemeen van de website?** Jah .. er staan er paar rare vragen op. Zo verschillende keer dat ze terugkomen op dezelfde vragen.. **en wat vond je positief?** Dat ze u wel bewuster maken. Misschien is dat het idee. Maar die vragen! |
