# Supplementary material for: Users’ thoughts and opinions about a self-regulation-based eHealth intervention targeting physical activity and the intake of fruit and vegetables: A qualitative study
Source: PLoS One. 2017 Dec 21;12(12):e0190020. doi: 10.1371/journal.pone.0190020 (PMC5739439; doi:10.1371/journal.pone.0190020)
Supplement: S3 File — This file contains the transcribed interviews. (ZIP) [file pone.0190020.s003.zip › type_2_diabetes/TA2BONI.docx]

**Code filmpjes:**

| Deel interventie | Minuten | Transcript |
| --- | --- | --- |
| DEEL 1  VRAGENLIJST | 0-10 | Telefoon nr invullen vind ik verschrikkelijk.. dan word je zo lastig gevallen te pas en te onpas .. beweging kies ik.  Neen, neen. Amai vreselijk he als je daar zo met je neus op gedrukt wordt.  **Het is fysiek actief zijn hé.** Ah, kan ik terug gaan? **ja.** ik ben eigenlijk de hele dag actief bezig, ruiten wassen, stofzuigen .. **dus elke dag 2 uur dan?** Het is per dag ja .. nee, pakt dan, 1:30.  Dju dat is weinig .. vreselijk.  Matige fysieke activiteiten .. in een week.. 5. Tijdens de werkuren .. pff .. alle dagen. ik ben toch wel een paar uur bezig. Elke dag. Dat gaat een uur een half zijn. zwaar.. neen. |
|  | 10-16 | Als deel van je werk .. bedoel je daarmee als ik training geef loop ik een hele dag door, mag dat er bij? **Als dat 10 minuten aan een stuk is.** Dan gaat dat maar 2 dagen zijn. Als je wandelt als deel van je werk in welk tempo is dat dan.. laag.  Hoeveel beweeg je denk je pffff als ik dit lees.. niet veel! Ben je van plan om meer te bewegen, waarschijnlijk wel als het zonneke meer gaat schijnen. Als de zon schijnt ga ik heel veel fietsen. Ik vind dat een moeilijke vraag, het is afhankelijk van het weer. Ik denk dat dat klopt ja, dat in ieder geval. Mij beter voelen ja. anderen mij steunen.. neen dat doe ik zelf. Meer kan bewegen dan ik nu doe.. hmmm.. joa. Ook in moeilijke situaties, neen.. als het regent kom ik niet buiten. Daar ben ik nu geen doorzetter se. Ik geef dat rap op als het voor mijn eigen is. Ik ben een opgever. Ik doe altijd iets voor een ander, of er moet een doel aan hangen om ergens te geraken. Ja. plannen doe ik maar uitvoeren is iets anders. Als ik een score heb dan zou ik het wel doen maar zomaar.. ik moet een doel hebben. Het zomaar bijhouden, neen .. ik heb een duidelijk plan voor wanneer ik meer zal bewegen: neen.. neen. Ik heb geen plan.  Dat vind ik een moeilijke vraag. Neen. Het komt zoals het komt. |
| DEEL 1 ADVIES | 16-17:30 | **Wat vind je hiervan?** Dat gaat me niet meer motiveren. Het is leuk om te weten maar wat heb ik daaraan, wat brengt mij dat bij.. **En wat zou voor u dan, als daar een grafiek ofzo zou bijstaan?** Ja! ik heb het nogal voor grafieken! Een stappenteller is voor mij fantastisch, dat ik kan zien van ik heb het gehaald of niet gehaald, maar dat zegt mij niet zo veel. Ik moet meer gepusht worden, ik heb die duw meer nodig. Dat is voor mij hier al te lang om te lezen.  Dat is niet haalbaar voor mij. Dat zijn dingen waar ik persoonlijk op zou afhaken, bewegingsdoelen van wat anderen hebben gekozen, dat interesseert mij niet, ik kan dat niet maken, ik vind dat heel tof dat die mensen dat doen maar ik kan dat niet halen, en dat zou mij negatief beïnvloeden. |
| DEEL 1 OPSTELLEN ACTIEPLAN | 17:30 -30 | Dat vind ik wel interessant dat actieplan, want dan kan je zeggen van, daar heb ik tijd voor of daar heb ik geen tijd voor.  Als het mij niet interesseert ga ik geen zelfdiscipline hebben..  De belangrijkste hindernis .. zelfdiscipline, is dat dan interesse? Ik weet het niet ..  Ik wil niet naar een sportclub, ik heb geen zin me nog eens te verplaatsen ’s avonds ..  Wat bedoelen ze daar nu mee… nog eens bij dat fietsen dan? Ja misschien als het mij interesseert, je hebt hier weinig keuze he. Ja of nee. Tjah .. hoeveel tijd .. hoeveel dagen zou mij beter passen, dat is zo moeilijk .. eigenlijk elke dag maar .. 3. Dat zijn de gemakkelijkste dagen. als het weekend is dan 6. Tja. Oe dat is lang hoor. Dan fiets ik toch wel een hele namiddag. Zelf een als-dan plan .. dat komt er bij mij niet van.. als ik dat doe dan komt het er niet van. Als ik een als-dan op stel.. dan is het alles of niets. Dat zit in mijn hoofd dus .. dan doe ik toch wel.. 30 kilometer aan de zee. Maar als ik dat ga zeggen tegen mijn eigen ‘als ik op maandagavond thuis kom dan’ dan gaat bv zoals nu een telefoon en dan kan ik niet meer gaan fietsen of joggen wat ik heb vooropgesteld had en dan wordt ik boos op mijzelf en gooi ik alles overboord. Indien je geen als-dan wilt maken .. dus ze vragen om het toch te doen? **Als je het niet wilt doen kan je gewoon schuine streep zetten.** Ik wil dat wel doen maar ik weet dat ik het niet ga doen. Ik ga het overslaan. Dan zet ik een schuine streep, en hier ook. Ja.  Op welke manier .. door mij meer actief te verplaatsen .. met de fiets.. op welke manier ..  Is er een reden dat dat nog eens terugkomt die als-dan? **Dat is omdat dat een andere activiteit is.** **Ik ga ik moeten vragen om de datum van vandaag in te vullen.** |
| DEEL 1 ACTIEPLAN | 30 | Ik wist zelfs niet dat ik dat geantwoord heb in het huishouden? Ik vind dat ik daar al genoeg in doe. Eigenlijk zegt mij dit niet veel. En nu verwacht ik zoiets van wat brengt mij dat op..  Dat wist ik niet dat mijn BMI zo hoog was.. dat heb ik wel geleerd. Ik dacht dat hij op 26 zat maar het is 27.  Dat steunt mij niet, ik moet dat zelf doen. Ik moet dat voor mijn eigen doen. |
| DEEL 2 VRAGENLIJST | *(2^e^ fragment)* | Op hoeveel dagen in de week goh.. om ergens heen te gaan.. pff. Oei. Hoelang wandel je gewoonlijk .. wandelen gaat niet snel met mijn been. De vragen zijn wel langdradig gesteld. Hoeveel wandel je in je vrije tijd. .. tempo laag.  Tijdens het werk. Hoeveel dagen .. matig … als deel van je werk ..  ….  Als ik dat niet bereikt heb dan ben ik niet gemotiveerd. Dit interesseert mij niet, ik wil gewoon weten wat mij dit heeft opgebracht, bijvoorbeeld ben ik goed bezig, behoor ik tot de midden categorie, kan ik beter..  als ze meer zouden vragen ‘wat wil je graag zelf verder doen’ maar dat is geen goede vraag voor mij, ik heb een korte vraag nodig ‘wat ga je doen’ ‘wat is de reden’ en dan zou ik er wel op antwoorden voor mezelf. Ik hou niet van lange vragen. |
