# Supplementary material for: Users’ thoughts and opinions about a self-regulation-based eHealth intervention targeting physical activity and the intake of fruit and vegetables: A qualitative study
Source: PLoS One. 2017 Dec 21;12(12):e0190020. doi: 10.1371/journal.pone.0190020 (PMC5739439; doi:10.1371/journal.pone.0190020)
Supplement: S3 File — This file contains the transcribed interviews. (ZIP) [file pone.0190020.s003.zip › type_2_diabetes/TA2BONO.docx]

**Code filmpjes:**

| Deel interventie | Minuten | Transcript |
| --- | --- | --- |
| DEEL 1  VRAGENLIJST | 0-10 | *(inleiding)* fysieke activiteit.. in en rond het huis, ik werk als vrijwilliger hier beneden dus dan werk ik nog 2 of 3 dagen in de week. Dat is maaltijden geven en met de oudjes bezig zijn.. moet ik dat hier ergens opschrijven? **Straks kan je dingen invullen**. Hoeveel dagen fiets je 10 minuten aan een stuk.. bijna alle dagen. 10 dat weet ik niet, het zal wel 10 minuten zijn. Ik ga hier opschrijven 7 dagen. Hoelang fiets je gewoonlijk op zo een dag, 10 of 20 minuten. |
|  | 10-20 | Het is te zien waar ik naartoe moet gaan he. Ik zal 20 opschrijven. Op hoeveel dagen wandel je 10 minuten om ergens heen te gaan, ja alle dagen he met mijn hond. Meestal is het alle dagen he. Hoelang wandel je op zo een dag. Oei nog een keer? Heb ik iets verkeerd gedaan. **Neen nu is het ‘hoe lang’.** Aah oke.20 minuten. De ene keer langer dan de andere keer.  Bijna alle dagen he met mijn hond. En je bent altijd in beweging he. En ik doe ook beweging als ik opsta he. Dan zet ik mij op mijn bed en doe ik een paar bewegingen, niet voor mijn suikerwaarden maar omdat ik zou willen vermageren. Hoeveel tijd besteed je in de tuin.. ik heb geen tuin! **De vorige vraag ging ook over de tuin, misschien moet je eens terug gaan**. Aaaah oke. Ik heb niet goed gelezen.  Tilwerk .. ik ga niet vragen aan de tuinieren of ik moet helpen met een boom om te zagen! Matige fysieke activiteit binnenshuis, dat is bijna alle dagen he. Pakt een uurtje. In verband met sport ontspanning tijd, dat zal meer ontspanning zijn, ik geef dansles dusja. En ik ga de vrijdag en zaterdag soms dansen. Ik ga ook gaan fietsen. Dat is ook een fysieke activiteit he. Wandelen .. volgende maand ga ik in de bergen wandelen he. Op hoeveel dagen in een gewone week aan matige fysieke activiteit.. fietsen aan een matig tempo.. soms is dat niet aan een matig tempo. Zwemmen kan ik niet. Boodschappen, met de hond wandelen dat is ook een matig tempo. Hoeveel tijd op zo een dag.. goh, een uur of 2 uur. Dat schommelt. Ik zal 2 opschrijven. Dat is nu wel niet alle dagen 2 uur he. Ongeveer, gemiddeld. **Ja**. Zware fysieke.. oei oei. Salsa heb ik nog gedaan, maar ik mag niet meer met mijn heupen. Soms doe ik ook fitness turnen en dat is ’s morgens, dat is moeilijk. Maar ik durf het af en toe wel eens doen. En dansen is ook een zware activiteit! Dan sta ik van 8:30 – 13 op de dansvloer zaterdag! Hoeveel dagen .. jah .. meestal 1 dag in de week zumba en fitness, 2 keer in de maand probeer ik te gaan. Dansles is dinsdag. **Ja schrijf maar wat je zelf denkt ongeveer.** Hoeveel tijd.. pakt een half uur of drie kwartier. Hoeveel dagen in een gewone week wandel je 10 minuten aan een stuk, met mijn hond ben ik zeker al 30 minuten onderweg. Dag is ook al alle dagen. In de zomer is dat wat meer. Middelmatig tempo, mijn vriendinnen zeggen altijd dat ik te rap ga. **Het is hoe je het zelf aanvoelt.** Werk je betaald of onbetaald. Het is vrijwilligerswerk. |
|  | 20 - 30 | **Als je het wilt meerekenen kan je ja invullen.** Als ik werkte dee ik dat ook maar niet alle dagen, nu doe ik dat wel alle dagen. Dragen van lichte lasten .. ik zou niet weten of een bord in de afwas steken een lichte last is haha ! **gohja..** dat is 5 dagen per week. Hoeveel tijd.. dat zal toch 2 uur zijn.. opdienen, afwassen .. een uur en twee uur. Hoeveel dagen in een gewone week doe je aan spitten enz.. dat doe ik niet! **Dat geeft niet, je moet niet bij alles iets invullen.** 10 minuten wandelen als deel van je werk, dat gebeurt niet dagelijks.. dat is misschien 1 of 2 keer per jaar, dus ik zal geen invullen. Hoeveel beweeg je denk je.. euhm .. niet te veel en niet te weinig, dat is er zo een beetje tussen haha! Ben je van plan meer te bewegen de komende maand, dat weet ik nog niet. Misschien wel misschien niet. Hoe moeilijk of makkelijk denk je dat het is .. moeilijk is dat nu wel niet. Je moet natuurlijk de tijd vinden hé. Als ik meer beweeg is mijn kans op ziektes kleiner, ja dat is just! Ik ben er zeker van dat ik meer kan bewegen dan dat ik doe .. euhm, daar ben ik niet zo zeker van. In feite kan je altijd meer bewegen als je met pensioen bent hé. Maar met dat vrijwilligerswerk heb ik niet altijd tijd, ik loop ook wel altijd rond, ik weet niet of dat meetelt.  Ook in moeilijke situaties.. ook als ik telkens opnieuw moet proberen, haha. Dat zal wel juist zijn. Ook als ik problemen en zorgen heb, ja dat is soms moeilijk hé. Moet ik dan fout aantikken? Ja als jij zegt van dan is het inderdaad moeilijker. Ook als mijn familie en vrienden dat niet doen, dat is niet juist he. Maar ik ben niet goed mee wat ze vragen. Dat is wel voor interpretatie vatbaar, je kan 2 dingen opvatten met die vraag. Alhoewel ik dat niet juist vind!  Ik heb er moeite mee om voor mezelf doelen te stellen, misschien wel misschien niet, het is te zien. Met de gezondheid is dat wel moeilijk. Ik heb er moeite mee plannen te maken, neen daar heb ik geen moeite mee. Ik hou mijn vooruitgang bij.. dat doe ik nu wel niet. Wat boelen ze daarmee, dat je het opschrijft ofzo? **Ja**. Dat doe ik niet. Ik heb een duidelijk plan voor wanneer ik meer ga bewegen.. mee eens. Ik heb een duidelijk plan voor hoeveel ik ga bewegen. Als je een plan hebt voor wanneer dan heb je ook een plan voor hoeveel he. Als er iets tussen mijn plannen komt.. juist he. Ik heb een duidelijk plan voor wat ik ga doen in situaties waarin het moeilijk is om meer te bewegen .. niet juist. |
| DEEL 1 ADVIES | 30 – 32 | Haha! **Je lacht zo?** Ja. Wil je graag een persoonlijk actieplan maken.. **voor het onderzoek zou je ja moeten aanduiden.** |
| DEEL 1 OPSTELLEN ACTIEPLAN | 32 – 38:30 | De volgende vraag .. dansen he! Haha! **Snel beslist.** Wil je graag een tweede activiteit … welke tweede, alfabetische lijst .. euhm. Tiens. Staat dat daar niet tussen.. fitness turnen staat er niet tussen? Aerobic ga ik nemen dan. Weet je al waar, ja hier beneden he. Waar zal je je eerste uitvoeren, ah ja.. euhm.. dat doe ik op mijn eigen he. Wat moet ik daar zeggen? **Waar je het gaat doen**.. parochiezaal he! **Ja dan mag je dat typen.** En het tweede, in het OCMW centrum? **Ja dat mag zeker!** Hoeveel dagen wil je dansen, dat is lesgeven, maar de vrijdag ga ik zelf dansen. Les volgen. Dan is dat 2 dagen in de week he. 2^e^ activiteit.. dat zal wel genoeg zijn, aerobic is intens he. Aerobic is maandag. Dat is ’s avonds he. Euhm. Als het avond is. En dan de tweede dat zal ’s morgens zijn. Hoe lang, oei oei, langer dan 60 minuten dan. 2^e^ activiteit is een uur. Als-dan plan: dus wacht hoor.. voor mijn eerste activiteit. Als ik op dinsdagavond thuis kom, dan ga ik 2 uur dansen? **Ja dat kan!** Is dat zo goed? **Ja dat doet er niet zoveel toe hoor.** **Hier mag je de datum van vandaag invullen.** |
| DEEL 1 ACTIEPLAN | 38:30 | Een boekske zeker.. ik doe dat wel niet maar allee! Ik zou het wel kunnen doen. **Je mag het versturen**. |
| DEEL 2 VRAGENLIJST | *(2^e^ fragment)* | **Nu moet je doen alsof je na 1 week terugkeert.** Aah ja. oe? Dat zijn dezelfde vragen als daarnet? Maar er is geen week voorbij he, dat vind ik wel raar. In feite is het altijd hetzelfde he dat je doet.. ik zal dat weer invullen. Dat zal toch hetzelfde zijn want het is altijd hetzelfde dat je doet. En op mijn leeftijd ga je niet meer gaan beginnen doen.  Het zijn allemaal dezelfde vragen weer precies..  Trappen lopen .. dat doe ik wel. In plaats van de lift durf ik de trappen doen. Alhoewel naar het 4^e^ is wel nen eind he. |
