# Supplementary material for: Users’ thoughts and opinions about a self-regulation-based eHealth intervention targeting physical activity and the intake of fruit and vegetables: A qualitative study
Source: PLoS One. 2017 Dec 21;12(12):e0190020. doi: 10.1371/journal.pone.0190020 (PMC5739439; doi:10.1371/journal.pone.0190020)
Supplement: S3 File — This file contains the transcribed interviews. (ZIP) [file pone.0190020.s003.zip › type_2_diabetes/TA2BOOI.docx]

**Code filmpjes:**

| Deel interventie | Minuten | Transcript |
| --- | --- | --- |
| DEEL 1  VRAGENLIJST | 0-10 | **Vanaf nu mag je beginnen lezen**. *(invullen gegevens)* **het is het beste dat je kiest waar je zelf nog moeite mee hebt.** Ik eet niet veel fruit omdat er veel fruit eet dat ik niet mag eten. Aardbeien zijn niet goed. Peren hebben ook veel suiker. Alle fruit heeft zijn eigen suikers he. En het heeft als uitgewezen als ik veel aardbeien eet is mijn suiker hoger dan anders. Ik eet fruit maar niet in grote mate. *(vult gegevens verder in)* **je mag zeggen wat je van de website vindt.** Op hoeveel dagen fiets je 10 minuten aan een stuk om ergens heen te gaan.. **waarom kijk je zo raar bij die vraag?** Ja het weer speelt een rol he.. **deze week nemen gewoon**. Ja, twee dagen ongeveer. Op hoeveel dagen… dagelijks he. Als ik van hier naar de markt ga en terugkeer ben ik 20 minuten goed he, ik ga 20 zetten. Het is te zien hoe goed ik me voel he, ik ga middelmatig pakken. Dat ook niet. Ik werk niet in de tuin he.. |
|  | 10-20 | Dat is niet aan mij besteed. Op hoeveel dagen, welja dagelijks he. Doe je aan matige activiteit binnenshuis. Ik stofzuig alle dagen, ik ga 7 zeggen, is dat goed voor jou? **Ja.** Bedden maken enal ja .. we gaan er een uur van maken, dat is eigenlijk wat veel maar dat schommelt he. De ene keer is dat iets minder en de andere keer is dat veel.. fietsen aan een matig tempo .. ik ga 2 dagen pakken. Want het is 4 keer een halve dag he. Wandel je 10min aan een stuk in je vrije tijd, dat moet ik niet invullen he, want als ik dat doe is het voor de boodschappen he. **Je moet wel ‘geen’ vullen.** Dat is een verplichte vraag .. voor mijn plezier ga ik niet wandelen ik zie dat niet zitten. Ik zou dat wel een keer doen, misschien 2 keer op een jaar als ik anders niet ben buiten geweest.  Werk je momenteel.. neen. Volgende. Hoeveel beweeg je denk je. Jah. Nu vraag je me wat. Heel veel, neen. Niet veel niet weinig.. dat ga ik pakken. Ben je van plan om meer te bewegen.. het is goed weer hé. Niet gemakkelijk niet moeilijk het is te zien hoe ik me voel, of men rug mee wilt. Als ik meer beweeg is mijn kans op ziektes kleiner.. kheb het al. Dus.. zal ik mij mentaal beter vullen, dat is weer zoiets waar ik niet veel weet op te zeggen. Dat weet ik niet. Ik zal maar zetten juist. Zullen anderen mij steunen, mijne vent trekt hem dat niet aan. Is mijn kans op het ontwikkelen van ziektes zoals diabetes kleiner, ja he.. als je meer beweegt. Als je niet beweegt dat is niet goed. Meer kan bewegen dan ik nu doe.. ja. Dat is planning he. Ook in moeilijke situaties.. als het regent ga ik k…. **wat vind je van die vragen?** Pff. Ben je er meer kan bewegen dan je nu doet, dat ligt gewoon aan je planning he, als er iemand komt koffie drinken ga je dat niet meer doen. Of je zegt ik ga vandaag mijn kasten uitkuisen, awel ja als je dat kiest dat ga je niet meer bewegen he. Het is ook bewegen maar het is niet hetzelfde he.  Regen en te weinig tijd, als het regent ga ik niet buiten lopen. Fout.  Ook als ik moet proberen tot het me lukt. Ja dat moet je doen he. Niet opgeven. |
|  | 20 – 24 | Ook als ik problemen zorgen heb. Helemaal juist. Ook als mijn familie en vrienden dat niet doen. Dat moet je zelf uitmaken he. Ik heb er moeite mee om voor mezelf doelen te stellen om meer te bewegen. Zeker niet. Soms heb ik er moeite mee, soms niet. De ene dag is de andere niet. Ik hou mijn voortgang bij.. zeker niet. Daar begin ik niet aan. Mee eens. Je moet een planning kunnen maken als je wilt. Ik heb nooit geen plan daarvoor. Als het niet gaat gaat het niet hé. Volgende. Heel matige intensiteit he .. ja soms naar de cardio fitness, maar op mijn tempo he. Ik zweet niet, of tis maar een klein beetje, en het is niet daar – **ahja ja. Het is rustig.** |
| DEEL 1 ADVIES | 24 – 25 | **Hier is het bedoeling dat je klikt op ‘ja’.** |
| DEEL 1 OPSTELLEN ACTIEPLAN | 25 - 36 | Denk je dat het moeilijk zal zijn meer te bewegen.. jah … dat is nu hmm.. **vind je het een vreemde vraag?** Voor mij persoonlijk is het moeilijk erop te antwoorden.. ik zit met een slechte rug he, dus als ik veel last heb kan ik niet veel doen, ik ga het nodige doen maar dat is alles. Ik beweeg wel maar ik hou mij rustig. Ik zal dan dit pakken. Ik ben rap moe he. Ik ga al 5 jaar wekelijks naar de kinesist. Dat is weer hetzelfde als daarvoor? Dat is twee keer hetzelfde. Onvoldoende gezondheid. Hmm. Ik weet niet wat ik moet invullen, en ik moet het invullen he. Gehandicapt ben ik niet! Onvoldoende gezondheid.. ik ben niet ziek, maar niet gezond ook.  Pffff …  **Wat vind je van die vraag?** Voor iemand die nog werkt die heeft meer keuze om in te vullen he .. Moest ik nog gaan werken zou ik dat pakken.. dan zou ik dat pakken. Door een sport uit te oefenen in mijn vrije tijd ga ik pakken. Paardrijden, haha ! **waarom lach je?** Omdat dat voor mij geen activiteit is..  In de fitnessclub op de rolband! Ik heb geantwoord he. Als ik daar ga is het 1 keer per week. Op welke dagen? Jah mensen! Als het past he. . 15 minuten op de loopband is al een eind he. Als-dan: ja.. een streepje. **Waarom ga je het zo laten?** Omdat ik geen vaste dagen heb. Ik heb wel ingevuld woensdag als het mij dinsdag uitkomt om te fitnessen ga ik dinsdag. Als het past dan past het. Het moet een beetje ingekaderd zitten in je week. Volgende. Vanaf wanneer? Wat moet ik hier invullen? **De datum van vandaag**. Volgende. |
| DEEL 1 ACTIEPLAN | 36 – 38 | Ik ga dat niet doorsturen, dat is voor mijzelf. Dagelijks bijhouden .. op de kalender zetten als ik geweest ben hé. Volgende. Wat moet ik nu nog doen? **Versturen gewoon**. cava. |
| DEEL 2 VRAGENLIJST | (*2^e^ fragment)* | Heb je de vorig keer een actieplan gemaakt om meer te bewegen.. ja. Volgende. 2 dagen. een traag tempo dat is heel traag he? **Ja**. oke dan matig tempo. Heen en terug .. als het goed gaat middelmatig als het slecht gaat is het een laag tempo .. geen. Hoeveel dagen.. in feite loop je alle dagen met je stofzuiger rond. Ruiten wassen vloeren schrobben of vegen.. hmm .. hoeveel tijd.. een uurke. Op hoeveel dagen in een gewone week doe je aan matige fysieke activiteit, dat is nog eens apart? Geen he. Het grootste gedeelte pak ik nog mijn auto. Werk je momenteel.. neen. Volgende. **De vraag is of je effectief op de loopband hebt gelopen**. Jah. Op hoeveel dagen. één dag. **Dat moet 15 minuten zijn dat is een foutje in de website.** Dat is twee keer hetzelfde. Als-dan. We gaan ons best doen he. Versturen? |
