# Supplementary material for: Users’ thoughts and opinions about a self-regulation-based eHealth intervention targeting physical activity and the intake of fruit and vegetables: A qualitative study
Source: PLoS One. 2017 Dec 21;12(12):e0190020. doi: 10.1371/journal.pone.0190020 (PMC5739439; doi:10.1371/journal.pone.0190020)
Supplement: S3 File — This file contains the transcribed interviews. (ZIP) [file pone.0190020.s003.zip › type_2_diabetes/TA2BOON.docx]

**Code filmpjes:**

| Deel interventie | Minuten | Transcript |
| --- | --- | --- |
| DEEL 1  VRAGENLIJST | 0-10 | *(vult gegevens in)* waarom hebben ze mijn naam nodig? Is die vraag relevant, ik snap het niet, of je een andere module hebt ingevuld? **Ja het is inderdaad misschien een vreemde vraag.** Waarom moeten ze dat allemaal weten? **Er was een actie als je meedeed met de studie.**  Is fruit niet gewoon fruit? Maakt dat veel verschil? **Dat is afhankelijk van het gewicht, als je 2 druiven hebt gegeten is dat anders dan dat je 2 kiwi’s hebt gegeten.** Deze vraag is ook wel raar geformuleerd.. hoe moeilijk of makkelijk het is ligt toch aan jezelf?  Deze is ook wel wat raar.. zullen anderen mij steunen.. deze is ook een moeilijke.. dat is een rare vraag .. **de derde vraag?** Ja.  Deze vind ik een niet zo nuttige vraag..  Deze is ook een beetje raar geformuleerd..  Haha, een fruitdoel.. ik vind dat raar.  .. |
| DEEL 1 OPSTELLEN ACTIEPLAN | 10:00 – 15:35 | Wat wordt er bedoeld met bepaalde situaties? **Daar moet je zeggen wat jij denkt dat het is**.  **Vind je dat een vreemde vraag?** Ja een beetje overbodig. |
| DEEL 2 VRAGENLIJST | *(2^e^ fragment)* | **Nu mag je doen alsof je een week verder bent.** Aah oke.  …  **Ja dat was alles!** |
| DEEL 2 AANPASSEN ACTIEPLAN |  |  |
| DEEL 2 REST |  |  |
