# Supplementary material for: Users’ thoughts and opinions about a self-regulation-based eHealth intervention targeting physical activity and the intake of fruit and vegetables: A qualitative study
Source: PLoS One. 2017 Dec 21;12(12):e0190020. doi: 10.1371/journal.pone.0190020 (PMC5739439; doi:10.1371/journal.pone.0190020)
Supplement: Supplementary file 44 [file pone.0190020.s003.zip › general_population/TA1BENO.odt]

De vetgedrukte tekst impliceert dat Louise aan het woord is. 

| Deel interventie | Minuten | Transcript |
| --- | --- | --- |
| DEEL 1 VRAGENLIJST | 0-10 (1e filmfragment) | Man die vragenlijst invult: Hoe oud ben je .. 24 jaar. Oké. Naar beneden scrollen? Groenten.. fruit .. Oké. Moet ik nu een ervan kiezen? Ja. Fruit. Oké. Welkom, bedankt dat je meedoet, 3 onderdelen... Wordt anoniem behandeld, oké wordt geapprecieerd. Volgende. Enkel voornaam? Zoals je wilt. Heb je al een andere module volledig ingevuld? … Neen. Niet dat ik weet. (vult zijn email adres in) Waarom lach je? Omdat het nu niet meer anoniem is, maar goed. Aah dus dat gaat verstuurd worden naar mijn e-mail adres? Ja! Oh, dat is leuk. GSM nummer.. is dat verplicht? Ja. Normaal gezien krijg je geen ongewenste berichten. Wat is je vaste lijn.. dat hoeft niet? Oké. Volgende. Wat is je leeftijd? Ja, 24. geslacht man. I hope so. Wat is je hoogste diploma, hmm ja momenteel algemeen secundair onderwijs. Volgende. Hoeveel weeg je? Goh, dat is een goede vraag. Het zal wel 93 zijn ofzo. Want ik ben 1m90. Dan schrijf je 190. oke. Ben je in behandeling voor diabetes? Neen. Voeg je een speciale voedingsrichtlijn of dieet? Neen. Hoeveel dagen heb je de voorbije week fruit gegeten? Hmm .. Tiens. Confronterende vraag eigenlijk. De voorbije week, ik denk echt geen enkel. Ik zou het liever anders hebben maarja, studentenleven. Hoeveel fruit denk je dat je eet? Weinig. Ben je van plan om meer fruit te gaan eten de komende maand? Misschien wel. ja. Misschien dat het actieplan me er ook wel toe aanzet om dat te doen. Dat is wel leuk. Hoe moeilijk of makkelijk denk je dat het is om in de komende maand meer fruit te eten? Ik denk dat het moeilijk is omdat het gaat over mijn levensstijl dus .. ja. Ik zal eens een peer komen in Delhaize. Dat moet me wel lukken. Als ik meer fruit eet dan is mijn kans op ziektes kleiner. Ik denk dat dat wel ergens juist is dat fruit gezond is. Zal ik mij mentaal beter voelen? Dat denk ik echt wel! Dat is puur pscyhologisch, maar dat denk ik wel. Zouden anderen mij steunen? Neen, dat denk ik niet. Anderen weten dat niet per se, dus. Alle ja ik weet het niet. Ik ben er zeker van dat ik meer fruit kan eten dan ik nu doe. Ja, helemaal juist! Ik ben er zeker van dat ik fruit ook in moeilijke situaties waarin minder fruit beschikbaar is. Dat is eerder fout, want dat is net de reden waarom ik niet veel fruit eet. Weinig tijd. Meer fruit kan eten ook als ik telkens opnieuw moet proberen tot het me lukt? Ja dat is misschien wel meer juist. Denk je dat anderen je zullen steunen? Jah, wat heeft dat er mee te maken zelfs. Ik vind dat dat volledig los van elkaar staat. Ik vind dat ik meer fruit kan eten ook als mijn familie of vrienden dat niet doen? ja. Natuurlijk. Dat is een eigen beslissing dat je neemt he. Dat kan wel. Oke. Ik heb er moeite mee om voor mezelf doelen te stellen om meer fruit te eten... ja. Ja zeker. Ik heb er moeite mee om plannen te maken die mij helpen om plannen om meer fruit te eten te bereiken. Ja, waarschijnlijk wel. Ik hou mijn doelen bij om na te gaan of ik meer fruit eet. Is dat over mijn situatie nu? Ja! Ah ja nee, dan zeker niet. Dat is iets waar ik totaal niet van wakker lig momenteel, dus. Volgende. Ik heb een duidelijk plan voor wanneer ik meer fruit ga eten. Momenteel nog niet, misschien na dit actieplan wel. Ik heb een plan voor hoeveel fruit ik ga eten? Misschien wel. Een peer ofzo of wat druiven. Per dag ofzo. Ik heb een plan voor wat ik ga doen als er iets tussen mijn plan komt. Neen, helemaal niet. Ik heb een plan voor in situaties waarin het moeilijk is om meer fruit te eten. Neen, mee oneens. Volgende. Het gezondheidsprogramma (fluistert... onverstaanbaar). Persoonlijk fruitddoel stellen. Waarom moet je lachen? Gewoon, dat is een woord dat ik nog nooit gelezen heb! Het is een mooi woord. |
| DEEL 1 ADVIES | 10-20 (1e filmfragment) | Kan je hieronder een voorbeeld zien van wat de anderen hebben gekozen.. Eric en Eline. zijn dat fictieve personen? Ja ja. Eric koos ervoor om 2 dagen per week 2 stukken fruit te eten. Dat is concreet. Wil je graag een persoonlijk actieplan maken om meer fruit te eten? Mobeja! Kom. We gaan de koe bij de horens vatten. Dat is een actieplan dat perfect past bij mijn profiel? Uhu. ja. |
| DEEL 1 OPSTELLEN ACTIEPLAN | 0-10 (2e fragment) vanaf 4:29 | Denk je dat je het in bepaalde situaties moeilijk zal hebben om meer fruit te eten? Tuurlijk! Bijvoorbeeld als ik geen tijd heb ofzo door lessen of hobbys. Dat is wel een goede vraag eigenlijk. Mag ik meerdere dingen kiezen? Ja. Oke. Door meer fruit bij mijn boterham te eten, ja, een yoghurtje. Door fruit bij de warme maaltijd, goh.. ja.. maar.. wat denk je? Appelmoes is toch echt niet hetzelfde als een verse appel die je eet. Neen, dat is waar. Dus dan denk ik dat dat het effect van gezondheid of gezond zijn eigenlijk verliest. Ik ga het aanduiden omdat ik dat wel wil doen, maar ik denk dat de twee eerste al gezonder zijn. Aperitief.. daar heb ik geen tijd voor. Fruit in salade? Neen, ik eet geen salade. Haha. Voorgesneden fruit.. oke. Bestaat dat ook al. Fruit op voorhand te bereiden .. dat zou zo leuk zijn als ik daar tijd voor had, maar nee nee. Voldoende fruit mee te nemen naar mijn werk. Neen. Door fruit op centrale plaatsen te plaatsen. Pff. Neen. Dat zou ik wel doen, dat ik fier ben op mezelf dat ik groenten en fruit eet. Hoeveel dagen per week wil je fruit eten? Eens denken. Ik denk tussen 3 of 4. ik zal 4 pakken. Als ik dat zou bereiken zou het al veel zijn, en dan kan ik dat erna nog opdrijven. Hoeveel porties fruit wil je eten op die dagen? Ik denk 1. ja. Waar? Goh.. euhm, thuis. En daarmee bedoel ik op mijn kot. Thuis. Bij familie en vrienden .. daar ben ik aan het denken: ik heb ooit wel een periode gehad dat ik gewoon een peer uit de hand at terwijl ik onderweg was met vrienden na de les ofzo.. mag ik dat dan hier invullen? ja. Dat wil ik wel opnieuw doen. Wanneer wil je vooral fruit eten? Hmm .. vooral tussendoortjes. In de voor of namiddag tussen de lessen een appel of peer eten. 's avonds .. neen. Zeker in de namiddag. Als het zo goed weer is. Dat heeft zijn charme. Als je zelf een als-dan plan wil opstellen .. dat snap ik ook niet goed. Amai dat is heel concreet. Geef aan wanneer je iets zal doen en wat. Euh .. Als ik .. hoeveel moet ik er zo invullen, of is het 1 voorbeeld? |
| OPSTELLEN ACTIEPLAN | 10 – 11 | Je mag kiezen, als je er meerdere wilt maken kun je er nog twee maken maar dat is niet verplicht. Neen, ik ga 1 goeie invullen. Zo in de namiddag he. Als ik in de namiddag een tussendoortje eet, tijdens de pauze, ik zal zeggen de lespauze. Dan zou dat een peer zijn. Is dat goed? Ja! Ik heb het wel voor peren. Als je nog extra zou .. neen, volgende. Dat lijkt mij gewoon ook echt heel haalbaar. Ja dat is belangrijk. Vanaf wanneer wil je meer fruit eten? Je moet vandaag ingeven anders gaat het systeem blokkeren. Oke, de 19e. Verplicht enal. Haha een beetje pressure mag. |
| DEEL 1 ACTIEPLAN | 11:28 – 14:29 | Amai dat is echt cool! Dat is zo concreet. Dan zal dat een peer zijn, haha. 25 tot 8, dat is er net over he? ja. Oei. Mijn huisarts?? dat staat er standaard bij als je er net iets over bent. Ah ja. Dat is dat je niets zou kunnen riskeren. Ik snap het wel. We raden je alvast aan om per dag niet meer dan 2 porties fruit te eten. Niet? Als ze suikerrijk zijn. Aah. Achzo. Oke. Check. … je je actieplan al dan niet volgt... kalender .. of een app, dat zou cool zijn. ja. Mijn GSM hou ik ook altijd bij tijdens de lessen, ik heb ook geen agenda ofzo. Een GSM gewoon dus. Wil je jou actieplan aan een familielid, vriend, collega tonen. Goh, waarom niet. Dan zal jou actieplan worden verstuurd naar die persoon. Dus ja aanduiden als ik dat wil? ja. Ik ga dat naar mijn ma sturen, haha, die gaat blij zijn! Wat was dat nu weeral, dat moet wel juist zijn. Ik hoop dat het juist is. Dat zijn allemaal personen naar wie ik kan sturen? Ja maar je bent niet verplicht die allemaal in te vullen! Oké. Versturen. |
| DEEL 2 VRAGENLIJST | (3e filmfragment) 0 – 2:51 | Tweede module. Zijn dat dezelfde onderdelen als de vorige? Je hebt nummer 1 en 2 al gedaan en nu doen we nummer 3. oké, ja dat klopt. ja. Je hebt de vorige keer een actieplan gemaakt om meer fruit te eten, ja. Dat klopt. Hier kan je nogmaals de doelen zien die je voor jezelf hebt gesteld.. dat mag je zelf bedenken van stel ik heb dat nu een week gedaan, denk je dat het zou gelukt zijn ofniet? Of in welke mate het zou gelukt zijn. Minder. 3 dan. Hoeveel heb je in de voorbije week gegeten? Een stuk of 3 peren gegeten. Dessertenschaaltjes .. jongens toch wat is dat hier allemaal.  Nul. Neen. Handen vol kersen. Neen. Kiwi's iew neen. Nectarines nul. T staan wel lekkere dingen tussen, maar nog niet gegeten. Lychees, neen. Ah neen. Ik heb wel een gekocht. Neen. Neen. Neen. Volgende. Ja. Versturen. |
| DEEL 2 AANPASSEN ACTIEPLAN | (4e filmfragment) 0 – 2:18 | Heb je de vorige keer een actieplan gemaakt? ja. Volgende. Dat is opnieuw. Na een maand .. misschien wel 2. dat gaat weer enkel over 4 peren ofzo. Dat is wel meer per dag. Het is allemaal hetzelfde als de vorige keer. Tijdsbeheer.. is dat voldoende? ja. |
| DEEL 2 REST | 2:18 – 3:15 | Ik ga juist nog een paar vragen stellen. Wat vond je van de website? Gebruiksvriendelijk wel. En goh, het is niet dat er zo heel veel herhaling zit in de vragen, want dat gebeurt soms in enquetes dat je zo constant dezelfde vragen krijgt, of dat je denkt dat het dezelfde vragen zijn! ja. ja. Nu is dat niet, het is duidelijk en concreet allemaal. En zijn er nog positieve zaken of negatieve zaken waar je aan moet denken? Over de website? Euhm .. neen, niet specifiek. Oke! |
