# Supplementary material for: Users’ thoughts and opinions about a self-regulation-based eHealth intervention targeting physical activity and the intake of fruit and vegetables: A qualitative study
Source: PLoS One. 2017 Dec 21;12(12):e0190020. doi: 10.1371/journal.pone.0190020 (PMC5739439; doi:10.1371/journal.pone.0190020)
Supplement: Supplementary file 44 [file pone.0190020.s003.zip › general_population/TA1BENT.odt]

| Deel interventie | Minuten | Transcript |
| --- | --- | --- |
| DEEL 1 VRAGENLIJST | 0-8 | Enkel voornaam? Voornaam is voldoende. Als er iets is dat je zegt van dat is positief of negatief je mag dat zeker zeggen. Je mag ook luidop lezen als je het moeilijk vindt om luidop te zeggen wat je denkt. ja. Aantal porties .. 3 tomaten. Wortelen .. dat staat regelmatig op de menu. Ik heb dat nu toevallig dit weekend gegeten. Rauwe groenten .. Er staan regelmatig groenten op de menu. Wat denk je bij dat onderdeel, omdat ik je zo heel veel zie fronsen. Ik eet liever rauwe groenten dan gekookte groenten, gekookte groenten, ik ga niet zeggen dat ik dat niet eet, maar liever rauw. Een kwestie van mind-setting he, als je denkt ik ga dat doen dan probeer ik dat wel. De laatste tijd meer dan vroeger. We weten allemaal dat groenten eten gezond is he. Je mag ook negatieve zaken verwoorden als je dat vindt. ja. De plannen, dat is niet voor mij he. Ik heb daar geen plan voor mijn vrouw bepaalt wat ik eet. Dat is omdat ik de planning niet maak.... Dat vind ik niet. Neen. Ik ga dat niet bijhouden, ik ga dat doen maar ik ga dat niet tellen. Kga er geen statistieken van maken. Je kan niet van de ene module naar de andere gaan nee? Je moet éne kiezen? Neen het is eigenlijk eentje, tenzij je groenten niet zo tof vindt en liever naar een andere gaat? Neen neen tis goed, we gaan het actieplan opmaken. |
| DEEL 1 ADVIES | 8 – 10 | (advies wordt niet voorgelezen) En wat vond je van het onderdeel van de samenvatting? Ik ben me ervan bewust dat ik te weinig groenten eet. Ik kom uit een andere generatie.. groenten … snel snel, snel eten is belangrijker voor mij, maar dat is verbeterd sinds dat mijn vrouw de keuken doet. Vroeger at ik altijd dagschotel en dat is niet bevorderlijk voor groenten.. wanneer of hoe gaan we dat doen.. wat denk je nu als je dat leest? Een broodmaaltijd kan je eigenlijk eens vervangen door een groentenmaaltijd. Salade dan ofzo? ja. De salade, daar focus ik meer en meer op. |
| DEEL 1 OPSTELLEN ACTIEPLAN | 10 – 13:40 | Je mag ook meerdere antwoorden aanduiden als je wilt. Hoeveel dagen groenten eten.. 5 op 7 toch wel. ja. Het zou wel tof zijn dat we die 300 kunnen halen. Ik mag er twee aanduiden he ja. Uhu. Als-dan. Dat vind ik moeilijk om zelf een als-dan plan te maken... wanneer en wat .. dat is een moeilijke. Niet dat ik veel wil veranderen, ik wil het gewoon stipter toepassen. Wanneer.. Als .. bijvoorbeeld boterkoeken vervangen door een stuk fruit, oei maar dat is geen groente. Maar je kan die boterkoeken ook vervangen door iets anders? Ik ga 's ochtends geen sla eten he.. Maar wel 's middags? ja. Je mag ook gewoon een tijdstip zetten bij 'als', bijvoorbeeld als het middag is, als het avond is … als het avond is is het tijd voor een sla'tje. Is dat goed? Ja zeker, het is jouw plan he. Voor de datum zou je vandaag moeten aanklikken want anders klopt het programma niet meer. |
| DEEL 1 ACTIEPLAN | 13 : 40 – 15:29 | Wat vind je van het actieplan nu het op papier staat? Ik vind het leuk dat het afgebakend staat, dat je ziet van eigenlijk moet ik dat meer doen. Het vrijblijvend karakter gaat een beetje weg. Wat vind je van dat deeltje? Ja. Ik ben ondertussen ook 7,5 maand gestopt met roken dus ik ben ook 5 kilo bijgekomen .. ik weet van waar ze komen dus het is niet dat ik dat al jaren meesleep. Dat is iets dat ik de laatste 7 maand meepak maar dat is een ander verhaal. Een app zou beter zijn, ik heb ook zo een app van gestopt te zijn met roken. Door die app wordt je alle dagen herinnerd dat je iets aan het doen bent en dat er iets op volgt, dat je alle dagen bevestiging krijgt van goed bezig, hou vol, je hebt al zoveel levensminuten gewonnen door te stoppen met roken. Ik ga het niet versturen, ik ga het voor mezelf doen. Versturen. |
| DEEL 2 VRAGENLIJST | (2e fragment) 0 – 2:15 | Algemene vragenlijst.. gezondheidsprogramma .. je mag altijd positieve en negatieve kritiek geven. Euhm .. ja. Heb je de vorige keer een test gedaan om meer groenten te eten? Ja we hebben dat gedaan he. Euhm .. 29/3 .. beeld je in dat je het een week hebt gedaan. Het is een beetje afhankelijk van wat ik graag eet he. ow. Ik heb iets overgeslaan. |
| DEEL 2 AANPASSEN ACTIEPLAN | 02:15 – einde + 3e fragment | Ja die porties, dat is moeilijk he. Wat bedoel je daarmee? Een portie ja, wat is een portie .. als je een slaatje eet moet je eigenlijk 10 porties eten. Ik ga dat een beetje verhogen he. Het is moeilijk om die porties in te schatten, ik ga die porties een beetje moeten aanpassen anders ga ik er niet geraken denk ik. Ik kom in de buurt. Dat komt weer he die vraag. Die vraag als en dan .. vanaf vandaag terug he als het kan. Je mag nog altijd positieve en negatieve zaken verwoorden he als je ze ziet. Als dan vind ik een moeilijk.. je kan dat moeilijk benoemen, omdat je daar jah .. ik ben een economist maar je kan niet zeggen het is 3 of 5 of 7 maar 'als dan' heeft zoveel ruimte langs alle kanten. Ja ik snap wat je bedoelt. Je mag ook terug zeggen wat er in je op komt. Ik vind dat ik goed geslaagd ben. (onverstaanbaar) tomaten wat minder want ik ben niet zo een liefhebber, wortelen, komkommers ook zelden, witloof wel. Gekookt niet. Het moet wel wat kloppen he. 5 dagen .. die module is wel lichtjes anders he. Ik had verwacht dat 3 nog eens een check-up ging zijn, of je te veel of te weinig hebt gegeten maar het is meer subjectief van 'vind je dat je geslaagd bent?' er zit niet echt een meter achter, allee misschien wel maar je krijgt geen feedback over je aantal gram terwijl je in 1 en 2 wel die feedback krijgt. |
| DEEL 2 REST |  | Dan heb ik nog een paar algemene vragen: zijn er zaken dat je zegt van dat vond ik echt goed dat dat er in zat? Ik vind dat je probeert te meten hoeveel groenten dat je eet vind ik wel goed, meten is weten. Als je het meet dan kan je het weten. Als je met de natte vinger begint dingen te doen en opvolgen jah.. als je het goed wilt doen dan moet je het meten. Maar ik ben daarin niet zo goed dus daar is een app echt ideaal voor. Dat je ergens ja .. moet. Het probleem is in dat systeem dat je veel input moet geven, als je stopt met roken bijvoorbeeld is dat gewoon 'iets niet meer doen'. Dan moet je maar 1 ding doen, en dat spel houdt alles bij. Bij jullie zou je het alle dagen moeten doen, liefst 2x. Vond je het te veel input dan nu? Ik denk dat je met een app, het eenvoudig moet houden, als je 2 of 3 keer per dag een input moet gaan doen is dat nie evident. Ik denk dat je het kunt oplossen – ik krijg alle dagen ook een bericht van dat spel – we zijn veel er dag verder, weer zoveel gespaard enzovoort, dan kun je zo iedere keer een stand van zaken zien. In groenten zou je dat eigenlijk ook moeten doen maar gewoon vragen van, 'heb je vandaag je portie groenten gegeten?' en ja of nee indrukken. Zodat je een barometer krijgt van je bent goed bezig of niet goed bezig. Stel bijvoorbeeld een weekend. Je moet de vrijdag gaan eten, je moet de zaterdag weg.. in een restaurant is dat niet evident om een slaatje te vragen. Je kan evengoed naar een restaurant gaan waar geen slaatjes op het menu staan hé. Als je nu juist in zo een weekend zit, dan eet je veel te weinig en je moet de feedback geven dan weet je, 'ik ben niet goed bezig'. Als je niets ingeeft, dan weet dat spel ook niets hé. Input is delicaat. Het vraagt actie van mensen, en dat is niet altijd evident. Je kan het vragen, je kan een bericht sturen, maar ze moeten het wel doen he. Toch denk ik dat het zeker interessant is voor mensen die hun gezondheid of hun levensstijl willen verbeter of veranderen.. zijn er dingen die je zegt van dat vond ik echt slecht? Die vragen 'als-dan' dat vind ik moeilijk. Omdat je er dan te veel gaat (onverstaanbaar) als je zegt van als ik er morgen aan denk ga ik groenten eten, het geeft te veel mogelijkheid om niet to the point te komen. Heb je concrete  punten van verbetering die je zelf ziet? Ik denk dat je moet focussen op het meten. Die porties vind ik ook moeilijk in te schatten want als je dat nu voor de eerste keer ziet, een slaatje telt voor 5 of 10, ik denk dat je daar misschien nog een klein beetje duiding moet geven als je porties vraagt dat je daar een referentiemaat moet bijzetten. Als je het de eerste keer ziet schat je het heel verkeerd in. Ik ga een slaatje als 1 portie beschouwen terwijl het eigenlijk misschien 5 of 10 is. Het staat er ook wel bij in gram he. Of is dat niet goed? Nja, maar een slaatje, kan je dat inschatten in gewicht? Een biefsteak kan je inschatten he, 200 of 300 gram, maar een slaatje .. dat weet ik niet. Het is niet evident om in te schatten. |
